# Structural Variation and Uniformity among Tetraloop-Receptor Interactions and Other Loop-Helix Interactions in RNA Crystal Structures

**DOI:** 10.1371/journal.pone.0049225

**Published:** 2012-11-09

**Authors:** Li Wu, Dinggeng Chai, Marie E. Fraser, Steven Zimmerly

**Affiliations:** Department of Biological Sciences, University of Calgary, Calgary, Canada; University of British Columbia, Canada

## Abstract

Tetraloop-receptor interactions are prevalent structural units in RNAs, and include the GAAA/11-nt and GNRA-minor groove interactions. In this study, we have compiled a set of 78 nonredundant loop-helix interactions from X-ray crystal structures, and examined them for the extent of their sequence and structural variation. Of the 78 interactions in the set, only four were classical GAAA/11-nt motifs, while over half (48) were GNRA-minor groove interactions. The GNRA-minor groove interactions were not a homogeneous set, but were divided into five subclasses. The most predominant subclass is characterized by two triple base pair interactions in the minor groove, flanked by two ribose zipper contacts. This geometry may be considered the “standard” GNRA-minor groove interaction, while the other four subclasses are alternative ways to form interfaces between a minor groove and tetraloop. The remaining 26 structures in the set of 78 have loops interacting with mostly idiosyncratic receptors. Among the entire set, a number of sequence-structure correlations can be identified, which may be used as initial hypotheses in predicting three-dimensional structures from primary sequences. Conversely, other sequence patterns are not predictive; for example, GAAA loop sequences and GG/CC receptors bind to each other with three distinct geometries. Finally, we observe an example of structural evolution in group II introns, in which loop-receptor motifs are substituted for each other while maintaining the larger three-dimensional geometry. Overall, the study gives a more complete view of RNA loop-helix interactions that exist in nature.

## Introduction

**Figure 1:**
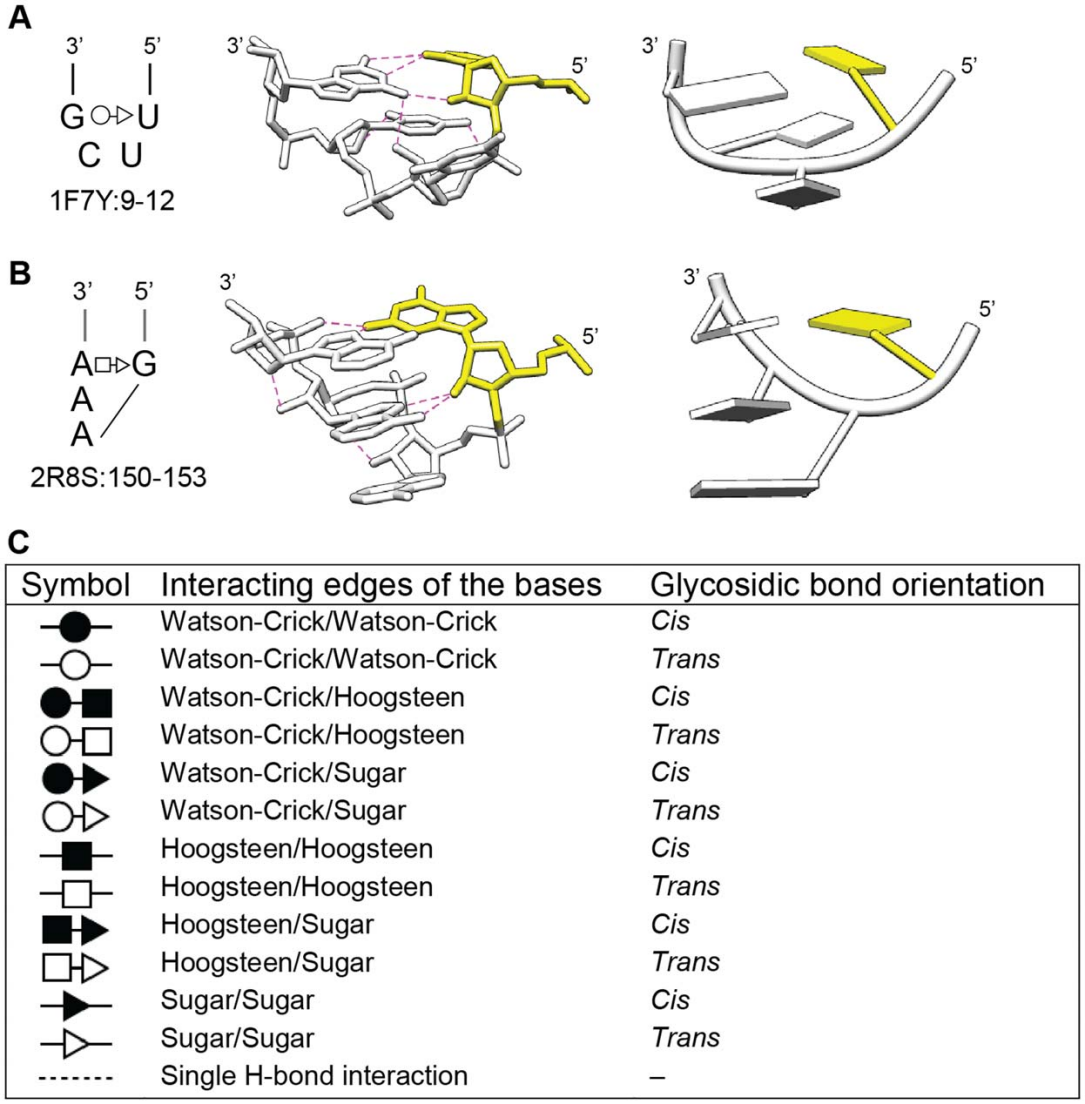
UNCG and GNRA tetraloops. A. The UNCG tetraloop. A secondary structure of an example tetraloop is shown to the left in the Leontis-Westhof notation. To the right are three-dimensional images in atoms/bonds and tube/slab formats. Dashed lines indicate hydrogen bonds, while yellow indicates the 5′ nucleotide. The figure is derived from PDB ID 1F7Y: 9–12. B. Secondary and tertiary structures of an example GNRA tetraloop, derived from PDB ID 2R8S: 150–153. C. Summary of the Leontis-Westhof notation for base-base interactions in RNA structures [26], [42].

Tetraloop-receptor interactions are common and well-studied organizers of RNA tertiary structure. It was noted long ago that the sequences of RNA tetraloops are not random, but fall into classes, of which two account for the majority of tetraloops in rRNAs (UNCG, GNRA) [1]. The UNCG tetraloop forms an especially stable structure [2], [3], with its closing nucleotides T1 and T4 forming a U-G base pair, and T2 and T3 extending on either side of the backbone (Figure 1A). (In this manuscript the four tetraloop positions are denoted T1, T2, T3 and T4). The GNRA tetraloop has a quite different geometry, with the bases of T2, T3 and T4 stacking on each other, and with T4 pairing with T1 through a non-Watson-Crick (non-WC) base pair [4] (Figure 1B). While GNRA tetraloops are less stable thermodynamically than UNCG tetraloops, they are more common, in part because of their propensity to form tertiary interactions [4], [5]. In addition, GNRA-type structures can be formed by longer loops (GNR[X_n_]A), and nonadjacent nucleotides can come together to form GNRA-like tetraloop geometries (GN/RA, where “/” indicates a sequence break) [6]. Other less common tetraloop motifs have been identified and studied, including CUUG, ANYA and AUCG [1], [7], [8], [9], but they do not typically form interactions with receptors.

GNRA tetraloops bind to at least three types of receptors, the 11-nt, IC3, and minor groove receptors. The 11-nt receptor is a specific structure first identified in the P4–P6 domain of the *Tetrahymena* group I intron, and it is characterized by an adenosine platform and multiple non-WC base pairs [10]. The receptor has specificity for the loop sequence GAAA, which stacks partially on the adenosine platform and forms multiple hydrogen bonds with the receptor. The GAAA/11-nt interaction is especially stable, and sometimes is introduced into RNA crystallization constructs to facilitate ordered molecular packing [11].

The IC3 receptor is found among group IC3 introns, and has a slightly different sequence compared to the 11-nt receptor. The IC3 receptor binds to GNRA sequences with lower affinity and lower sequence specificity [12]. Both the GAAA/11-nt and GNRA/IC3 interactions have been studied biochemically through mutagenesis and *in vitro* selection approaches, and these studies have identified functional sequence variants and provided insight into their binding selectivity [5], [12], [13], [14].

In contrast to the 11-nt and IC3 receptors, the minor groove receptor is a relatively simple motif, consisting of the minor groove surface of two helical base pairs. The GNRA tetraloop fits into the groove and forms two triple base pairs between T3, T4 and the two receptor base pairs; however, somewhat different conformations have been reported for GNRA-minor groove interactions [4], [15], which raises the issue of how many distinct structural variations exist for tetraloops binding in the minor groove.

Over the past decade, many studies have been undertaken to identify recurrent motifs in RNA structures. Recurring structural motifs can be considered the basic building blocks of RNA structure, and once identified, they can be used to predict three-dimensional geometry from primary sequence. A common approach is to search through a set of X-ray crystal structures for superposable substructures, with superposable nucleotides considered to be a recurrent motif. Structural similarity (i.e., superposability) has been evaluated based on a variety of criteria, such as RMSD (Root Mean Square Deviation) values, backbone geometries, and other parameters [6], [8], [9], [16], [17], [18]. Using such approaches, many RNA motifs have been identified, including the A-minor motif, the E-loop, the C-loop, the kink-turn, ribose zippers and others [19]–[24]. Recurrent motifs vary substantially in size and structural complexity. Some of the smaller motifs are components of larger motifs. For example, the A-minor motif consists of three nucleotides and is a component of both the GAAA/11-nt and GNRA/minor groove interactions [19].

In this study, we have taken a complementary approach to identify structural patterns in RNAs. Rather than searching for nearly identical substructures within a set of RNAs, we have first assembled a collection of tertiary structures composed of loop-helix interactions, and then asked a different set of questions: How many RNA sequences and structural conformations can form loop-receptor-type interactions? How much sequence and structural variation can occur for a given loop-helix pattern? Do the same sequences always fold into the same structures? Our compilation produced a set of 78 nonredundant loop-helix interactions, of which about half correspond to the already-characterized GAAA/11-nt and GNRA/minor groove interactions. The remaining interactions are either unique conformations, variants of the typical GNRA/minor groove receptors, or additional minor classes. The analysis provides a more complete picture of loop-helix motifs that exist in RNA structures.

## Results and Discussion

### Collecting a Nonredundant Set of Loop-helix Structures

To collect a set of structures containing loop-helix interactions, we compiled crystal structure files from the Protein Data Bank for all RNA and RNA-protein structures containing RNAs of >50 nts and resolutions of <4 Å (see Materials and Methods). The resulting set of 1348 PDB files was screened for redundancy to eliminate structures of virtually identical sequences, which left a set of 41 PDB structures (Table S1). Structures of less than 100 nts were examined visually for loop-helix interactions, while for larger structures, loops were located in published secondary structures and PDB files were examined visually for an interacting helix. For each identified loop-helix interaction, a PDB substructure file was extracted. This process assembled a set of 78 loop-helix interactions from 21 PDB files (Table S2). Thus, only about half of solved RNA structures >50 nts contain a loop-helix interaction.

### Generating Structure-based Groupings of Loop-helix Interactions

Loop-helix structures were categorized into nested groups using a combination of computational tools and visual inspection. Structures were initially subdivided into four groups based on secondary structure and gross tertiary structure characteristics. The groupings were refined by structure-based clustering functions, using RMSD values of superposed backbone atoms (see Materials and Methods for details). This process produced a set of nested subclasses (e.g., 1.1.1, 1.1.2, 2, 3; Table 1). Structures that did not form further levels of clusters were denoted “individual” (Indiv). For example, structures denoted 1.1(Indiv) are superposable with each other, but with larger RMSD values than pair-wise superpositions within the 1.1.1/1.1.2 subclasses. Loops having greater or fewer than four nucleotides were tagged NTL (non-tetraloop), and their conformations were compared with the tetraloop classes, using automated superposition and visual inspection (see Materials and Methods). NTL structures that superposed with tetraloop structures were grouped with the corresponding tetraloop classes (Table 1).

**Table 1:** GNRA tetraloop/helix interactions and GNRA tetraloop-like/helix interactions.

A. GAAA tetraloop/11-nt receptor							
Class	Subclass^1^	PDB ID	Loop nts	Receptor nts	ED^2^	RNA	Source
1		1NBS	205–208	145–150, 159–163	+	RNase P specificity domain (type B)	*Bacillus subtilis*
I		1U6B	24–27	146–151, 160–164	++	Group I ribozyme with both exons	*Azoarcus sp.*
I		1U6B	189–192	60–65, 80–84	++	Group I ribozyme with both exons	*Azoarcus sp.*
I		2R8S	150–153	222–227, 247–251	++	P4–P6 group I ribozyme domain	*Tetrahymena thermophila*
B. GNRA tetraloop/minor groove receptor (and GNRA-like structures having similar interactions)							
II	1.1.1	2Z75	114–117	10–11, 30–31	++	GlmS ribozyme RNA	*Thermoanaerobacter tengcongensis*
II	1.1.1	3IGI	90–93	272–273, 280–281	+	Group IIC ribozyme	*Oceanobacillus iheyensis*
II	1.1.1	3OK7	93–96	3–4, 340–341	+	RNase P holoenzyme, tRNA (type A)	*Thermotoga maritima*
II	1.1.1	3OK7	285–288	75–76, 84–85	+	RNase P holoenzyme, tRNA (type A)	*Thermotoga maritima*
II	1.1.1	3OFO	1077–1080	16–17, 918–919	++	16S rRNA	*Escherichia coli*
II	1.1.1	3OFO	1266–1269	1311–1312, 1325–1326	++	16S rRNA	*Escherichia coli*
II	1.1.1	3OFR	2857–2860	1708–1709, 1749–1750	++	23S rRNA	*Escherichia coli*
II	1.1.2	1U9S	205–208	80–81, 93–94	+	RNase P specificity domain (type A)	*Thermus thermophilus*
II	1.1.2	3MXH	32–35	59–60, 78–79	++	c-di-GMP riboswitch	*Vibrio cholerae*
II	1.1.2	1VQO	1629–1632	1553–1554, 1567–1568	++	23S rRNA	*Haloarcula marismortui*
II	1.1.2	1VQO	1863–1866	1467–1468, 1474–1475	++	23S rRNA	*Haloarcula marismortui*
II	1.1 (Indiv)	1Y0Q	22–25	170–171, 177–178	+	Group I ribozyme	*Staphylococcus* phage Twort
II	1.1 (Indiv)	1Y0Q	205–208	60–61, 78–79	+	Group I ribozyme	*Staphylococcus* phage Twort
II	1.1 (Indiv)	1VQO	469–472^3^	773–774, 887–888^3^	++	23S rRNA	*Haloarcula marismortui*
II	1.1 (Indiv)	1VQO	577–580	1110–1111, 1252–1253	++	23S rRNA	*Haloarcula marismortui*
II	1.1 (Indiv)	1VQO	1327–1330^3^	905–906, 1299–1300^3^	++	23S rRNA	*Haloarcula marismortui*
II	1.1 (Indiv)	1VQO	2630–2633^3^	2114–2115, 2470–2471^3^	++	23S rRNA	*Haloarcula marismortui*
II	1.1 (Indiv)	3OFO	1013–1016	987–988, 1217–1218	+	16S rRNA	*Escherichia coli*
II	1.1 (Indiv)	3OFR	1807–1810	1362–1363, 1368–1369	+	23S rRNA	*Escherichia coli*
II	1(Indiv)	1X8W	323–326	118–119, 202–203	+	Group I ribozyme	*Tetrahymena thermophila*
II	1(Indiv)	1VQO	734–737	2382–2383, 2405–2406	+	23S rRNA	*Haloarcula marismortui*
II	1(Indiv)	3OFO	898–901	769–770, 809–810	++	16S rRNA	*Escherichia coli*
II	1(Indiv)	3OFO	1516–1519	1404–1405, 1496–1497	++	16S rRNA	*Escherichia coli*
II	1 (NTL)	1MFQ	169–174	126–127, 223–224	+	7S RNA of SRP	*Homo sapiens*
II	1 (NTL)	1NBS	175–179	132, 234–235	+	RNase P specificity domain (type B)	*Bacillus subtilis*
II	1 (NTL)	1U9S	182–188	135–136, 162–163	+	RNase P specificity domain (type A)	*Thermus thermophilus*
II	1 (NTL)	2GDI	67–72	21–22, 37–38	++	TPP riboswitch	*Escherichia coli*
II	1 (NTL)	3D2V	55–59	13–14, 25–26	++	TPP-specific riboswitch	*Arabidopsis thaliana*
II	1 (NTL)	1VQO	119–121	50–51, 110–111	++	23S rRNA	*Haloarcula marismortui*
II	1 (NTL)	1VQO	873–877^3^	1832, 1844^3^	++	23S rRNA	*Haloarcula marismortui*
II	1 (NTL)	1VQO	1055–1059	2491–2492, 2529–2530	++	23S rRNA	*Haloarcula marismortui*
II	1 (NTL)	1VQO	1077–1082^3^	2067–2068, 2077–2078^3^	++	23S rRNA	*Haloarcula marismortui*
II	1 (NTL)	1VQO	1499–1506^3^	1420–1421, 1443–1444^3^	++	23S rRNA	*Haloarcula marismortui*
II	1 (NTL)	1VQO	1991–1997^3^	2583–2584, 2594–2595^3^	++	23S rRNA	*Haloarcula marismortui*
II	1 (NTL)	3OFO	1166–1170	1088–1089, 1096–1097	+	16S rRNA	*Escherichia coli*
II	1 (NTL)	3OFR	956–961	2456–2457, 2494–2495	++	23S rRNA	*Escherichia coli*
II	2	1VQO	691–694	2439–2440, 2452–2453	++	23S rRNA	*Haloarcula marismortui*
II	2	3OFR	630–633	2401–2403, 2414–2415	+	23S rRNA	*Escherichia coli*
II	2	3OFR	1364–1367	186–187, 209–210	++	23S rRNA	*Escherichia coli*
II	2 (NTL)	1VQO	1469–1473	156–157, 179–180	++	23S rRNA	*Haloarcula marismortui*
II	2 (NTL)	1VQO	2390–2398	915–916, 927–928	++	23S rRNA	*Haloarcula marismortui*
II	3	1LNG	163–166	208–209, 212–213	+	SRP19-7S.S SRP RNA complex	*Methanocaldococcus jannaschii*
II	3	1MFQ	147–150	197–198, 201–202	+	7S RNA of SRP	*Homo sapiens*
II	3	3KTW	164–167	209–210, 213–214	++	SRP19/S-domain SRP RNA complex	*Sulfolobus solfataricus*
II	3 (NTL)	1VQO	2564–2569^3^	2695–2696, 2699–2670^3^	++	23S rRNA	*Haloarcula marismortui*
II	4	3OFO	159–162	341–342, 347–348	+	16S rRNA	*Escherichia coli*
II	4 (NTL)	1VQO	1595–1599	1537–1538, 1647–1648	++	23S rRNA	*Haloarcula marismortui*
II	5	3IGI	369–372	128–129, 234–238	+	Group IIC intron	*Oceanobacillus iheyensis*
C. GNRA tetraloop-like structures interacting with a helix in other ways							
III	1 (NTL)	1VQO	2837–2843^3^	2087–2088, 2656–2657^3^	++	23S rRNA	*Haloarcula marismortui*
III	1 (NTL)	3OFR	642–646	2348–2349, 2368–2369	++	23S rRNA	*Escherichia coli*
III	Indiv (NTL)	3DIL	125–129	23–24, 68–69	++	Lysine riboswitch bound to lysine	*Thermotoga maritima*
III	Indiv (NTL)	1VQO	218–222^3^	164–165, 170–171^3^	++	23S rRNA	*Haloarcula marismortui*
III	Indiv (NTL)	1VQO	1706–1712^3^	790–791, 823–824^3^	++	23S rRNA	*Haloarcula marismortui*

1 “Indiv” indicates an individual structure that does not form an additional level of subgrouping based on superposition. “NTL” denotes a loop that does not consist of four nucleotides (non-tetraloop).

2 “++” indicates at most minor deviations between the electron density map and the specific modeled substructure (∼8–15 nts). “+” indicates a greater degree of unmodeled positive or negative electron density.

3 The *E. coli* ribosome substructures are nearly identical to *Haloarcula*, and are omitted from the table.

Up to this point, clustering was based on backbone superpositions, independently of base sequences or base conformations. To address base geometries, we compared Leontis-Westhof notations of base-base interactions for each structure (Figure 1C, Figure S1). The Leontis-Westhof notation simplifies base-base interactions as occurring between three possible hydrogen-bonding edges of bases (the Watson-Crick, Hoogsteen and sugar edges), and with two possible glycosidic bond orientations (*cis*- as in A form helices, or *trans*- with the base flipped 180°) [25], [26]. In general, the structural clusters were already consistent with base geometries; however, the additional information allowed minor refinements, so that each structural cluster is highly similar with respect to base geometry as well as backbone geometry.

Finally, we examined electron density maps of the crystal structures to evaluate the resolution of the specific substructures being analyzed. Since we were interested in the fit of individual residues to the electron density map, only local regions of the structures were evaluated. Substructures were categorized as “+” or “++” to indicate the degree of agreement between electron density and the atomic models for the ∼8–15 nt substructures. Structures marked “++” have at most minor discrepancies from the electron density map, while “+” structures have a greater degree of unmodeled positive or negative density (Tables 1, 2; see Materials and Methods). Interestingly, information about the structural resolution did not change the conclusions significantly. For example, motifs having “less standard” structures (below) did not correspond to the less resolved substructures (Tables 1, 2).

**Table 2:** Non-GNRA-like loop structures that interact with helices.

Class	Subclass^1^	PDB ID	Loop nts	Receptor nts	ED^2^	RNA	Source
A. Non-GNRA structures interacting with a helix							
IV	1 (NTL)	2A64	98–107	55–56, 392–393	+	RNase P RNA (type B)	*Bacillus stearothermophilus*
IV	1	3OFR	124–127	54–55, 115–116	++	23S rRNA	*Escherichia coli*
B. Single base inserted into receptor helix							
IV	Indiv (NTL)^2^	2QBZ	100–106	21, 167–168	+	M-Box riboswitch aptamer domain	*Bacillus subtilis*
IV	Indiv^2^	1VQO	2301–2306^3^	952, 1014–1015^3^	++	23S rRNA	*Haloarcula marismortui*
C. Surface formed by splayed nts							
IV	Indiv (NTL)^2^	1VQO	2069–2076^3^	2490, 2531^3^	++	23S rRNA	*Haloarcula marismortui*
IV	Indiv (NTL)^2^	3OFR	2210–2214	1359–1360, 1371–1372	++	23S rRNA	*Escherichia coli*
IV	Indiv (NTL)^2^	3OFR	1493–1497	1418–1421, 1577–1580	+	23S rRNA	*Escherichia coli*
D. Other unique interactions							
IV	Indiv (NTL)^2^	1VQO	196–200	415–416, 424–425	++	23S rRNA	*Haloarcula marismortui*
IV	Indiv 2	1VQO	1770–1773^3^	1829, 1885, 2017–2018^3^	++	23S rRNA	*Haloarcula marismortui*
IV	Indiv (NTL)^2^	1VQO	1834–1842^3^	2621–2622, 2642–2643^3^	++^4^	23S rRNA	*Haloarcula marismortui*
IV	Indiv (NTL)^2^	1VQO	1917–1922	418–419, 2448–2449	+	23S rRNA	*Haloarcula marismortui*
IV	Indiv (NTL)^2^	3OFO	461–470	202–203, 214–215	+	16S rRNA	*Escherichia coli*
IV	Indiv^2^	3OFO	523–526	11–12, 22–23	++	16S rRNA	*Escherichia coli*
IV	Indiv (NTL)^2^	3OFR	159–167	2206–2207, 2217–2218	++	23S rRNA	*Escherichia coli*
IV	Indiv^2^	3OFR	226–229	409–410, 417–418	++	23S rRNA	*Escherichia coli*
IV	Indiv (NTL)^2^	3OFR	2552–2556	2507, 2581–2582	++	23S rRNA	*Escherichia coli*
E. Single base makes almost all of the interaction, not a large interaction surface							
IV	Indiv (NTL)^2^	1VQO	391–398	2441–2442, 2450–2451	++	23S rRNA	*Haloarcula marismortui*
IV	Indiv (NTL)^2^	1VQO	671–675	36, 446	++	23S rRNA	*Haloarcula marismortui*
IV	Indiv (NTL)^2^	1VQO	838–845	1369–1371, 2054–2055	++	23S rRNA	*Haloarcula marismortui*
IV	Indiv (NTL)^2^	1VQO	2784–2788^3^	1153, 1213^3^	+	23S rRNA	*Haloarcula marismortui*
IV	Indiv (NTL)^2^	3OFR	1728–1732	1516	+	23S rRNA	*Escherichia coli*

1 “Indiv” indicates an individual structure that does not form an additional level of subgrouping based on superposition. “NTL” denotes a loop that does not consist of four nucleotides (non-tetraloop).

2 “++” indicates at most minor deviations between the electron density map and the specific modeled substructure (∼8–15 nts). “+” indicates a greater degree of unmodeled positive or negative electron density.

3 *Haloarcula* structural portions found in the *E. coli* ribosome with essentially identical structures. The *E. coli* structures are omitted from the table.

4 The electron density indicates that base 1835 should be flipped 180 degrees around the glycosidic bond.

### The Identified Classes and Subclasses of Loop-helix Interactions

In the end, our analysis resulted in four classes of loop-helix structures: I) the GAAA-tetraloop/11-nt receptor configuration; II) the GNRA-minor groove interaction in which a tetraloop or a tetraloop-like structure interacts at or near the minor groove of a helix; III) NTL loops having GNRA tetraloop-like structures and interacting with a helix in a manner different from Class II; and IV) non-GNRA-like loop structures that interact with helices in novel ways. All structures are displayed individually in Figure S1, and are available as PDB files in Structures S1.

**Figure 2:**
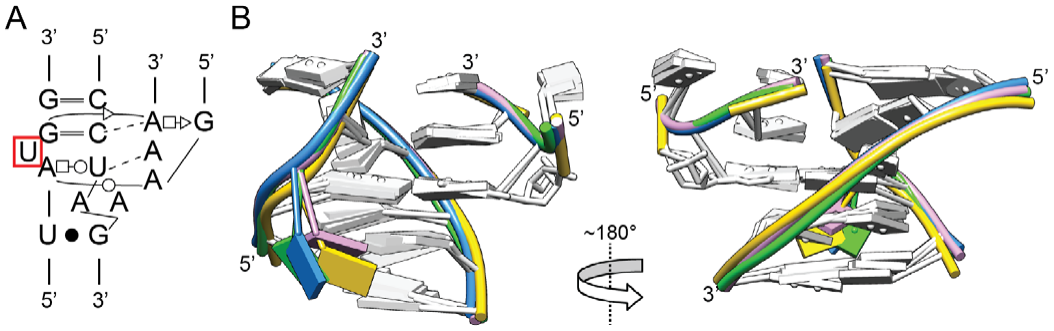
Class I loop-helix interaction: the GAAA tetraloop/11-nt receptor. (A) Secondary structure and (B) two rotational views of the three-dimensional structure of the GAAA tetraloop/11-nt interaction. Four structures are overlaid (1NBS:205–208, green; 1U6B:24–27, pink; 1U6B:189–192, yellow; and 2R8S:150–153, blue), which have nearly identical sequences, and overlay with pairwise RMSD’s of <0.92 Å. The colored bases shows the differences in position of the bulged U base (red box in Panel A).

Interestingly, Class I structures (GAAA/11-nt) are represented by only four examples in the set of 78 (∼5%), and are notably absent from rRNAs. Three GAAA/11-nt motifs have identical 15-nt sequences that agree perfectly with the motif consensus [13], while the fourth example has an A to C substitution for one adenosine platform residue. The four structures have virtually identical three-dimensional structures (<0.81 Å pair-wise RMSDs, based on all backbone atoms of T1, T4 and the 11-nt receptor), with the only significant difference being the position of the receptor’s bulged U base, which in one structure is angled to form hydrogen bonds with the adenosine platform (Figure 2).

**Figure 3:**
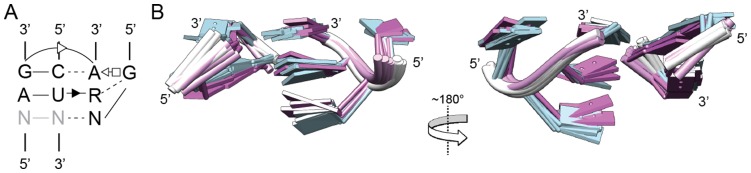
The Class II Subclass 1.1.1/1.1.2 loop-helix interaction. (A) Secondary structure of 1.1.1/1.1.2 interactions (consensus of 11 sequences). Gray nucleotides are not shown in the three-dimensional structure. (B) Two rotational views of an overlay of 11 members of Class II/Subclass 1.1.1 (blue; mostly GYGA loops) and 1.1.2 (purple; mostly GNAA loops).

Class II is the largest of the structure-based classes, and contains 48 of the 78 structures (62%). Its members fall into five distinct subclasses, which basically depict five ways that a GNRA-type tetraloop structure can interact with a helical surface. Of these, the most prevalent type is that of subclasses 1.1.1 and 1.1.2 (11 structures), and we consider this geometry to be the “standard” GNRA/minor groove motif structure. The 1.1.1 and 1.1.2 structures are virtually identical, although they were distinguishable by Chimera, and roughly correspond to GNGA and GNAA loops, respectively. The eleven structures superpose uniformly with low RMSD values based on the backbone atoms (<0.64 Å for 1.1.1; <0.82 Å for 1.1.2; <1.18 Å for 1.1.1/1.1.2 combined; Figure 3). In this configuration, the bases of the tetraloop and receptor are approximately coplanar, and T4 and T3 form two triple base pairs with receptor bases in the minor groove. In addition, the base of T2 forms hydrogen bonds with the receptor backbone, and there are two ribose zipper contacts between the backbones of the loop and the two receptor strands (not shown), which serve to stabilize the tetraloop with respect to the receptor. In this manuscript, we use the term ribose zipper to refer to ribose-ribose hydrogen bonds between backbones, regardless of the number of ribose moieties involved. Essentially all loop-helix interactions involve at least one such contact, but the numbers of ribose groups and hydrogen bonds are variable. Usually, at least one of the ribose zipper contacts occur outside of the tetraloop and receptor sequences, and so are not shown in the figures, but can be seen in the PDB files in Structures S1.

Other members of Class II Subclass 1 (i.e., Subclasses 1.1 (Indiv), 1 (Indiv) and 1 (NTL)) deviate to different degrees from the structure of Subclasses 1.1.1/1.1.2. In some of these, particularly for 1 (Indiv), the tetraloop bases are rotated substantially out of plane with the receptor bases, but hydrogen bonding still occurs in the central region of the minor groove (Figures S2, S3).

Structures of Class II/Subclasses 2, 3 and 4 deviate from Subclass 1 primarily because the hydrogen-bonding interface is shifted from the center of the minor groove. For Subclass 2, T4 forms hydrogen bonds with the backbone of the receptor, and T2 and T3 (rather than T3 and T4) interact with the bases in the minor groove (Figure S4A–D). Subclass 3 is a special case because the receptor is at the end of a stem-loop; the interaction might alternatively be described as occurring between two tetraloops. Here the “bottom” receptor base pair is the non-WC closing base pair of the tetraloop, and one of the receptor bases angles out of plane to form a coplanar base pair with T3 (Figure S4E–G). In Subclass 4, the tetraloop is shifted approximately 10 Å from the position in Subclass 2, so that the tetraloop interacts with mainly one strand of the duplex receptor (Figure S4H–J).

**Figure 4:**
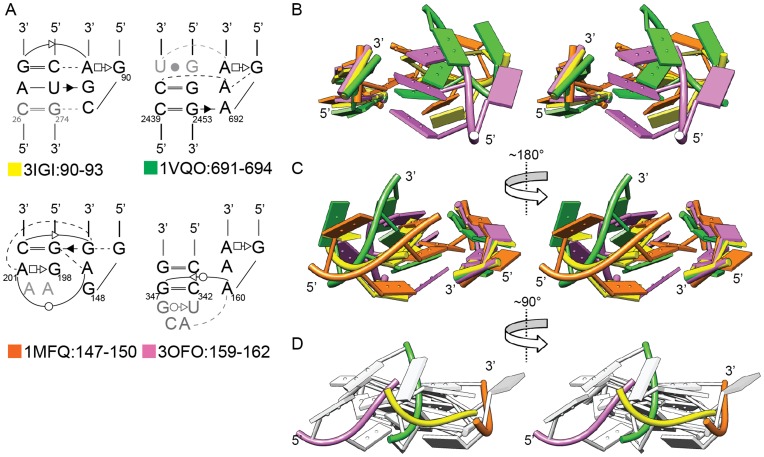
Comparison of Class II Subclasses 1, 2, 3, and 4, with one representative per subclass. A) Secondary structures of four representative interactions: Subclass 1, 3IGI:90–93 (yellow); Subclass 2, 1VQO:691–694 (green); Subclass 3, 1MFQ:147–150 (orange); Subclass 4, 3OFO:159–162 (pink). Gray nucleotides are not shown in tertiary structures. B, C, D) Stereoviews of the four-structure overlay, with superposition based on backbone atoms of the four receptor nucleotides (48 atoms). In Panel D, only the tetraloop backbones are colored in order to depict differences in the tetraloop orientations.

Figure 4 compares the structures of Class II Subclasses 1 to 4, with one example for each Subclass, and with superposition based on the 2-bp receptors in order to depict relative positions of the tetraloops. The standard subclass 1.1.1/1.1.2 structure is shown in yellow as a reference. For subclasses 2, 3 and 4 (orange, green and pink, respectively), the 3-base stacks of the tetraloop are rotated and/or shifted substantially relative to subclass 1 (Figure 4A, 4B). The displacement is best seen in the view in Figure 4D, in which the superposed receptors are hidden at the back of the image, while the colored backbones show both rotation and shifting of the tetraloops relative to the receptor surface. Interestingly, the loop backbone of Subclass 2 (green) is rotated ∼90° relative to Subclass 1 (yellow), and the loop positions of Subclasses 3 and 4 differ by approximately 15 Å. The figure illustrates the different configurations by which GNRA tetraloops can interact with minor grooves.

**Figure 5:**
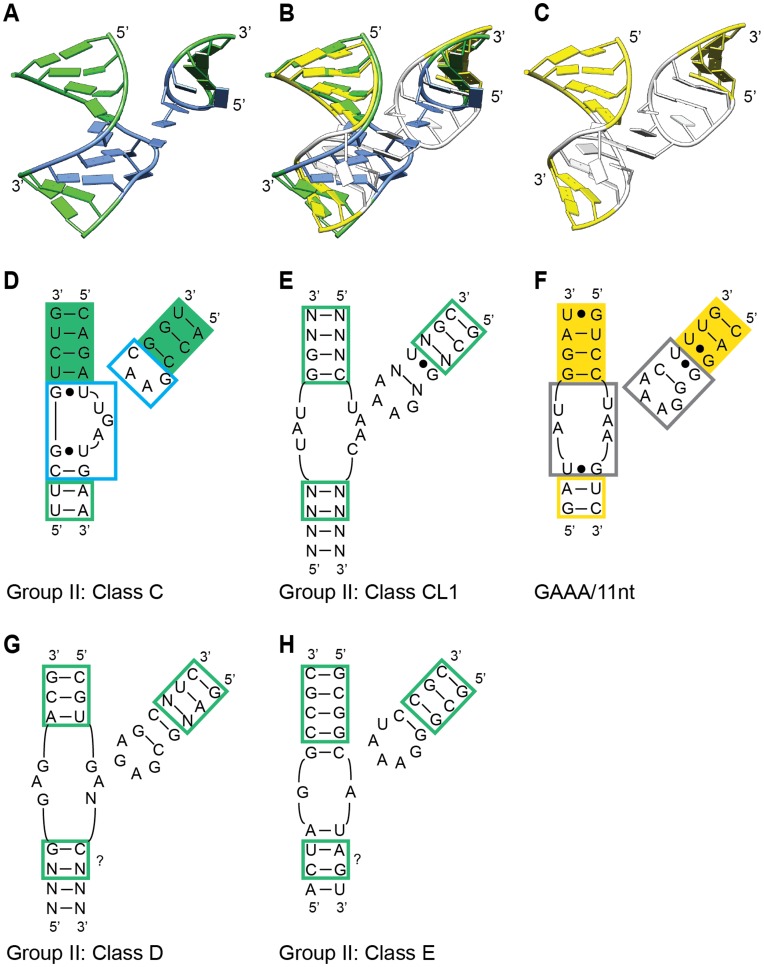
Comparison of ζ-ζ’ tetraloop-receptor structures in different lineages of group II introns. A) Class II Subclass 5 structure from 3IGI:369–372, which represents IIC introns (class C). Blue indicates receptor and tetraloop nucleotides. B) Overlay of structures in panels A and C, with superposition based on backbones of the nucleotides with solid green and yellow shading in Panels D and F. C) The GAAA/11 nt structure from 2R8S:150–153 (group I intron). White indicates receptor and tetraloop nucleotides. D-H) Secondary structures for ζ-ζ’ interactions in different lineages of group II introns (Panels D, E, G, H) or the GAAA/11 nt interaction in a group I intron (Panel F). The blue and black boxes in (D) and (F) correspond to the blue and white nucleotides in (A) and (C). The question marks in (G) and (H) indicate putative corresponding nucleotides to the green and yellow boxed nucleotides in (D) and (F), which superpose in (B).

Class II Subclass 5 consists of a single structure, but we consider it to be a class here, because its secondary structure motifs are shared among a lineage of group II introns, and the structure is expected to be shared as well [27] (and see below). The loop-helix interaction of Subclass 5 is unlike the other subclasses because there are no hydrogen bonds between the tetraloop bases and the “receptor.” Instead, the loop-helix interaction consists of a one-base stack of T2 on a flipped out base of the receptor, and a ribose zipper contact between the backbone of T4 and a receptor strand (Figure 5A). The consequence is that the tetraloop crosses the minor groove but is too far away to contact the minor groove bases. Similar to Figure 4, superposition of all five Class II structures based on tetraloops illustrates a dramatic range of geometries that receptors can assume relative to tetraloops (Figure S5).

Structures of Class III consist of non-tetraloop sequences having GNRA-like geometries, which interact with helices in arrangements distinct from Class II. Of its five members, only two share a common structure (Figure S6 A,B). As was the case for Class II, there is wide variation in the loop positions of Class III relative to the 2-bp receptors (Figure S7).

For Class IV structures, the loops do not form GNRA-like structures, and the interactions do not closely resemble GNRA-receptor interactions. Only two of the structures have a common structure, in which two stacked A’s fit into the minor groove and form interactions resembling GNRA interactions; however, the stacked A’s do not conform closely to the GNRA structure (Figure S6C, S6D).

**Figure 6:**
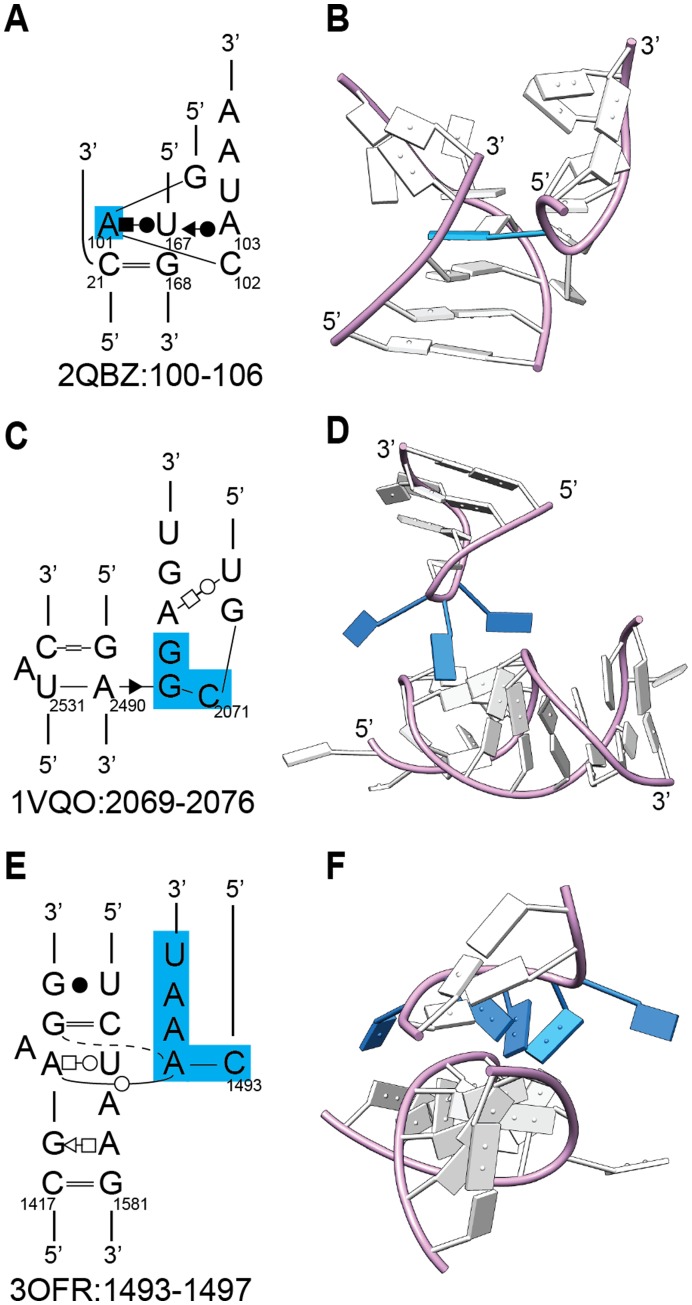
Three unique loop-helix interactions. A) Insertion of a loop nucleotide into a helix (2QBZ:100–106). B) Three splayed nucleotides interacting with a helix (1VQO:2069–2076). C) Three stacked nucleotides interacting in the minor groove, with two splayed nucleotides (3OFR:1493–1497). For all panels, blue indicates the distinctive features explained in the text.

The remaining Class IV structures are unique interactions, although some common themes can be observed. For example, in two structures (2QBZ:100–106, 1VQO:2301–2306), a flipped-out loop nucleotide inserts into the minor groove of the receptor, stacking with and base pairing with helical bases (Figure 6A). Three structures (1VQO:2069–2076, 3OFR:2210–2214, 3OFR:1493–1497) have interaction surfaces with splayed nucleotides in the loop structure that extend the interaction surface (Figure 6B, 6C). Another interesting example is the UNCG loop of 1VQO:1770–1773, which forms an interaction in which T2 (the “N” of UNCG) forms a Watson-Crick base pair with a receptor nucleotide (Figure S1 D4). Thus, UNCG tetraloops can interact with helical receptors in some contexts.

**Figure 7:**
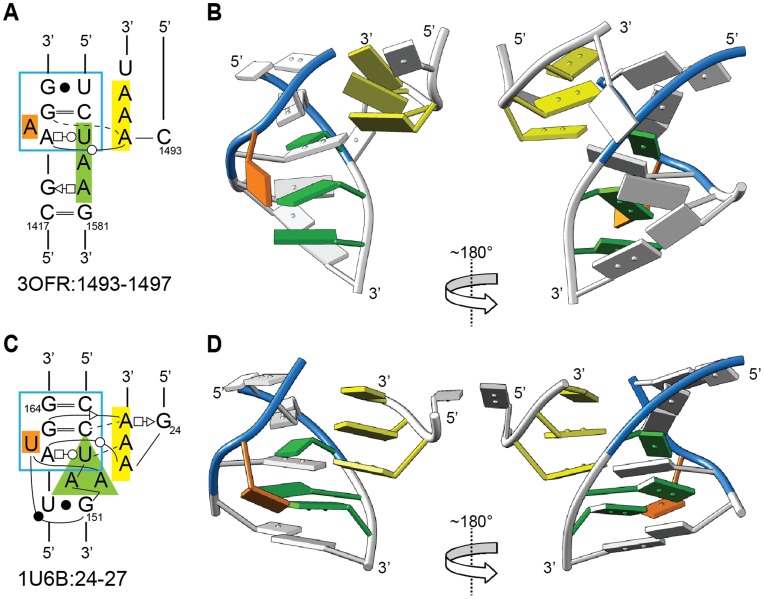
The loop-helix interaction 3OFR:1493–1497 resembles the GAAA/11 nt motif in secondary structure, but assumes a distinct three-dimensional geometry. A, C) Secondary structures for 3OFR:1493–1497 (Class IV(individual)) and 1U6B:24–27 (Class I, or GAAA/11-nt). Corresponding distinctive residues are coded in yellow, orange, or green shading, or are boxed in blue. B, D) Two rotational views of the structures, with residues colored as in (A) and (C). The residues with blue backbones superpose with an RMSD of 1.35 Å between the two structures, while the two green A’s form an adenosine platform in 1U6B:24–27 but form a stack in 3OFR:1493–1497.

One Class IV structure resembles the GAAA/11-nt interaction in its secondary structure, and to some extent in its tertiary structure (3OFR:1493–1497) (Figure 7A, 7B). Like the GAAA/11-nt structure, the receptor contains a bulged nucleotide (orange in Figure 7) flanked by a G-C and A-U WC-Hoogsteen base pair, with T2 forming a *trans*-WC base pair with the A. These five nucleotides and an adjoining base pair (blue boxes in Figure 7A, 7C) superpose well between the two structures (1.35 Å, 7 nts/84 backbone atoms), indicating a shared structural motif. However, the lower nucleotides adopt quite a different structure, with the AA internal loop of 3OFR:1493–1497 forming a two-base stack rather than an adenosine platform; furthermore, the position of the loop bases are shifted about 4 Å in the direction away from the AA sequence.

### Sequence-structure Correlations

Having assembled and categorized a set of loop-helix interactions, we looked for correlations between sequence and structure. Considering simply the loop structures, it is notable that tetraloops with a GNRA sequence almost always fold into the typical structure, with one exception (30 of 31 examples) (Figure S8A). The exception is for the GAAA sequence of 3OFR:124–127 (Figure S3–D1), which breaks from the expected geometry so that its two stacked A’s can reach the receptor’s minor groove and form hydrogen bonds. Conversely, some non-GNRA sequences fold into GNRA-like structures, including UCAA, GAAC and GNAG. All of these loops substitute a different non-Watson-Crick configuration for the closing G-A pair of GNRA (Figure S8B).

In examining the entire set of interactions, the most obvious sequence-structure correlation is the GAAA/11-nt loop interaction (Class I), for which the receptor sequences are nearly identical within the set, and invariably bind a GAAA tetraloop. A second correlation is for GNAG loops (Class II/Subclass 3), which bind to a terminal stem-loop receptor of the sequence 5′CGRAAG (Figure S4E, S4F). For this interaction, both the loop and receptor sequences are unique, and potentially diagnostic of a structure. A third apparent correlation is for the GAAC loop sequence (Class II/Subclass 5), in which both the tetraloop and receptor have conserved sequence motifs and corresponding structures [27] (see below).

Focusing on GNRA tetraloops, it is notable that while there are eight possible sequence combinations, they are not uniformly represented. In order of frequency, they are: GAAA (15/31), GUGA (5/31), GCGA (4/31), GCAA (3/31), GUAA (2/31), GAGA (1/31) and GGAA (1/31), while GGGA was not encountered (Table S3). Of these, the GCGA tetraloop is always found with the AG/CU receptor sequence in a Class II/Subclass 1.1.1 structure, while GUGA binds multiple receptor sequences, but always forms ClassII/Subclass 1.1 interactions. In contrast, the tetraloop sequence GAAA forms interactions of many types, including Class I, Class II/Subclass 1.1, Subclass 1, Subclass 2, Subclass 4, and Class IV/Subclass 1.

Similarly for 2-bp-receptors, only two sequences are commonly used out of 36 possibilities. Among Class II interactions, the most common 2-bp-receptors are AG/CU (10/29), GG/CC (7/29), CC/GG (2/29), CG/CG (2/29) and AG/CG (2/29), with other sequences being present only once (Table S3). All AG/CU receptors are found in Class II/Subclass 1 interactions, with all but one being Subclass 1.1. Other receptor sequences notably do not correspond to specific structures. The receptor sequence GG/CC for example, forms five types of interactions, which belong to Class II/Subclasses 1.1, 1, and 4, Class IV/Subclass 1, and Class IV (Individual).

**Figure 8:**
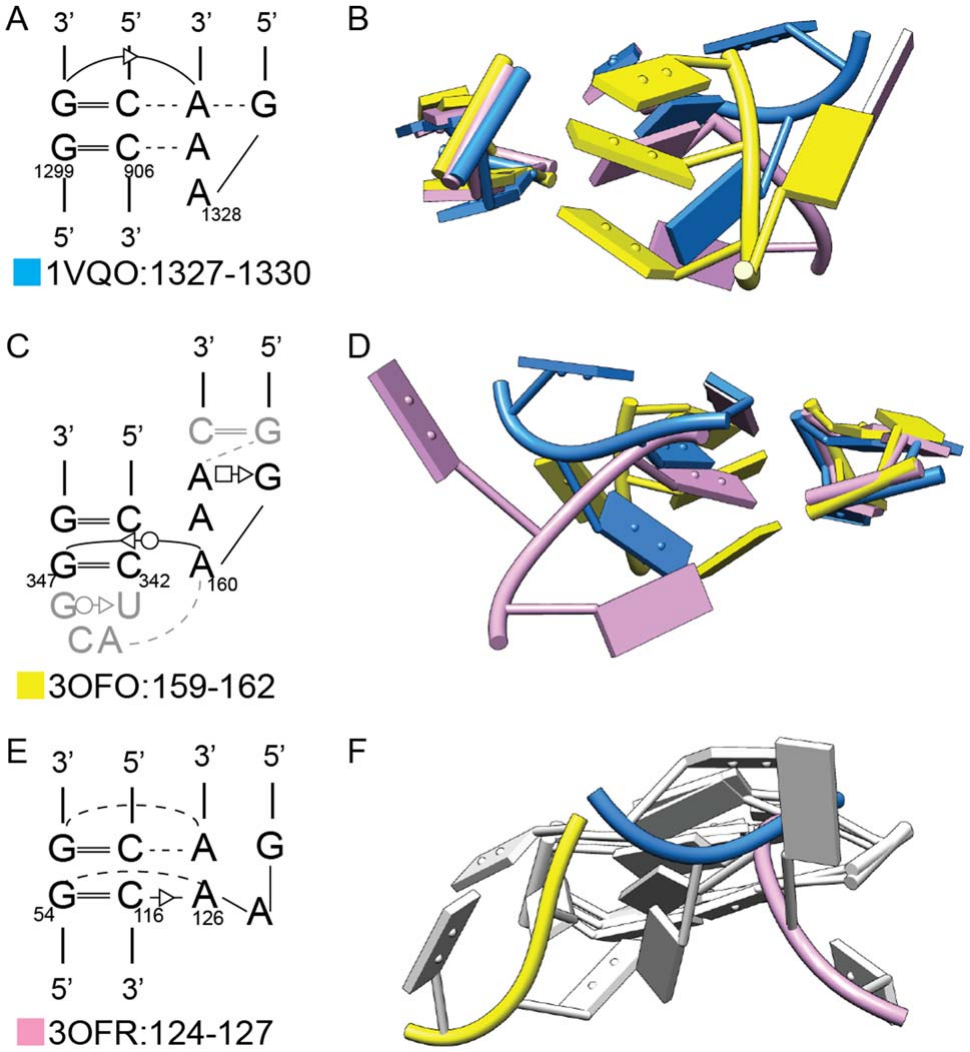
Three distinct three-dimensional structures formed by a GAAA tetraloop and GG/CC receptor. A, C, E) Secondary structure depictions of three structures. B, D, F) Three views of the superposition of 1VQO:1327–1330 (yellow), 3OFO:159–162 (blue) and 3OFR:124–127 (pink). In Panel F, only the backbones are colored.

Interestingly, interactions between a GAAA tetraloop and GG/CC receptor show a lack of sequence-structure correlation. Their sequences occur in three different geometries, belonging to Class II/Subclass 1.1, Class II/Subclass 4 and Class IV/Subclass 1 (Figure 8). Thus, while GAAA and GG/CC sequences are among the most common loop and receptor sequences, their structures are not predictable.

**Figure 9:**
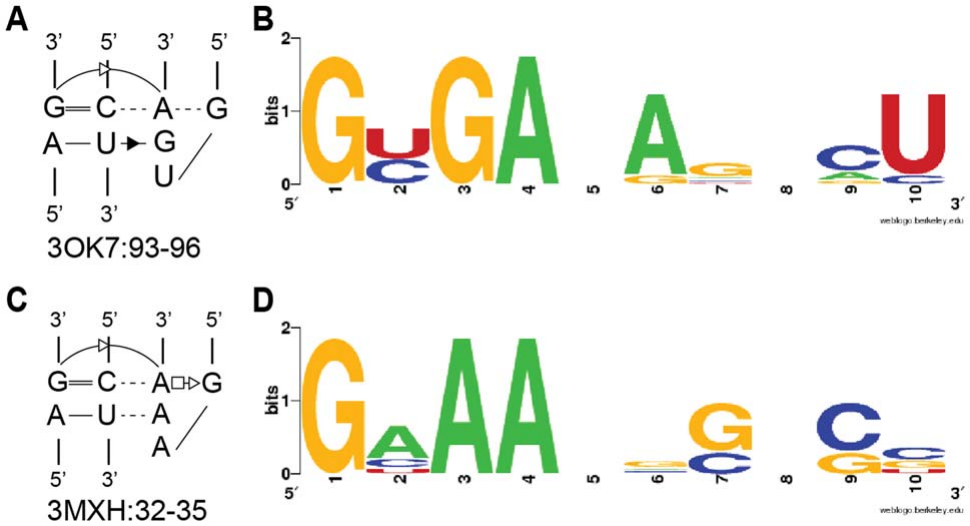
Weblogo profiles for GYGA and GNAA tetraloops and their respective receptors. A, C) Secondary structures of representative GYGA- and GNAA-containing interactions. B, D) Weblogo profiles of the two types of interactions.

A final sequence-structure correlation is detectable for GYGA and GNAA tetraloops within the structural class II/Subclass 1. A correlation was previously inferred based on the observation that GUGA tetraloops interact with AG/CU receptors, while GUAA tetraloops interact with GG/CC receptors [13], [28], [29]. The implication was that the third base (A or G) formed a triple base pair with a receptor base pair (either A-U or G-C). Our assembled data repeats the observation, but with an inexact and more complex correlation. Weblogo profiles for receptors of GYGA and GNAA loops suggest an eight-nucleotide correlation that is more complicated than a single, interchangeable triple-base pair (Figure 9).

In summary, the data set suggests a number of sequence-structure correlations as follows: the 11-nt receptor and the GAAA tetraloop; the GNAG loop and CGRAAG receptor; GYGA loop and AR/YU receptor; and GYAA loop and RG/CY receptor. There are also correlations between specific sequences and structural subclasses: the GUGA tetraloop forms ClassII/Subclass 1.1 structures; and the AG/CU receptor forms Class II/Subclass 1 structures. Each of these correlations may be useful as a starting hypothesis in predicting structure from sequence. In contrast, the lack of sequence-structure correlations for GAAA tetraloops and GG/CC receptors cautions against predicting their structures.

### Structure-function Considerations

While compilations and statistical correlations are useful, they do not substitute for experimental characterization. An unaddressed issue is the binding affinities for the various interactions identified. This ultimately can only be resolved experimentally; nonetheless, a few predictions are suggested. First, the GAAA/11-nt motif can be hypothesized to have the strongest and most specific interaction in the set because of its many hydrogen bonds and stacking interactions (Figure 2). No other interactions have such a complex interaction network. It remains possible, however, that some of the more complicated interactions may have significant binding strengths (e.g., 2QBZ:100–106, 1VQO:2301–2306, 3OFR:1493–1497). Among the large set of GNRA-minor groove interactions, we predict that Class II/Subclass 1.1.1/1.1.2 (Figure 3) has the greatest binding strength, judging from its frequency in the data set (i.e., nature has used the interaction motif repeatedly).

A second issue is whether structures deviating from the more standard geometries do so because of RNA flexibility, because of structural distortion, or due to adaptability in the loop-helix specificity. An examination of crystal structures of ribosomes in different conformations [30]–[32] did not identify differences in the loop-helix interactions, suggesting that they do not contribute to ribosomal RNA conformational changes. Examination of variant structures of Class II/Subclass 1(Indiv) (Figure S2C, S2D), showed that the sequences deviate from Subclass 1.1.1/1.1.2 and cannot form the same hydrogen-bonding network as for the “standard” structure. Thus, we suspect that in most cases, the structural deviations are not due to conformational flexibility or structural distortions, but represent alternative specificities made possible by sequence and structural contexts.

Plasticity in GNRA/minor groove geometries may be rationalized as follows. The interactions basically consist of contacts between two hydrogen-bonding surfaces–the three stacked tetraloop bases and the minor groove of the helix. Complementarity of the two hydrogen-bonding surfaces is dependent on the specific bases involved, such that certain geometries will be favored or disfavored by the base identities. In this view, GNRA-minor groove interactions have an intrinsic adaptability to deviate from the “standard” geometry of Subclass 1.1.1/1.1.2. While the deviations may come at a cost to binding strength, they would also permit additional ways for RNA segments to pack against each other.

### Evolution of a Loop-helix Interaction: an Example from Group II Introns

Because tetraloop-receptor interactions are important for the folding and stability of RNAs, it is of interest to consider how they may evolve over time, in terms of both sequence and structure. In group II ribozymes, the ζ-ζ’ interaction is a GAAA/11-nt receptor interaction [5], which is especially important for the structure of aI5γ, a well-studied intron [33]. However, the GAAA/11-nt sequence motif is not found across all group II introns [34], [35], which indicates that other sequence motifs fulfill the same structural role–or at least be compatible with same overall fold of the RNA.

In the group IIC intron crystal structure, the ζ-ζ’ interaction is not a GAAA/11-nt motif (Class I in this study), but a Class II/Subclass 5 structure (Figure 5A). At first glance, it is surprising that both motifs are used for ζ-ζ’ interactions, because the Class I and Class II/Subclass 5 structures are so different in sequence and geometry. However, the exchange of motifs makes sense when the larger structures are overlaid. Although the GAAA/11-nt motif of group II introns has not been crystallized, its structure can be approximated by the motif in *Tetrahymena* group I intron, whose sequence is highly similar (Figure 5C, 5E, 5F). When the structures are overlaid based on the nucleotides with green and yellow solid shading in Figure 5D, 5F, the flanking helices are positioned almost identically, while the ζ-ζ’ interacting nucleotides do not overlay well. This example shows that two completely different loop-helix structural motifs have been exchanged among group II introns, while the helices that they anchor are maintained in the same positions.

Among other lineages of group II introns, the sequence patterns for ζ-ζ’ are not consistent with either the IIC or GAAA/11-nt motifs, which implies the existence of additional interaction surfaces. These sequence patterns include a 4-bp stem-loop having a pentaloop (Figure 5H; Class E, F), and a GAGA tetraloop interacting with a symmetrical receptor sequence (Figure 5G; Class D). These lineage-specific motifs may be middle ground structurally and evolutionarily between the extremes of the IIC and GAAA/11-nt motifs. Neither of these sequence patterns is represented in our structural compilation, which indicates that available crystal structures do not contain a complete set of possible loop-helix interaction motifs. Consistent with this conclusion, the IC3 receptor motif is not present in any crystal structures, although it has been characterized experimentally [12]. Overall, the structural evolution of ζ-ζ’ appears to have involved modest sequence changes that resulted in dramatically different interaction structures and presumably binding strengths. Hence, what was conserved over evolution was the larger geometry, while the specific molecular basis for the interaction (i.e., structural motif) was not conserved.

### Conclusions

We have compiled a set of loop-helix interactions and examined diversity in both sequence and structural motifs. As expected, the GAAA/11-nt receptor and GNRA-minor groove interactions are the most predominant types of interactions in the set. However, we distinguish several subtypes of GNRA-minor groove interactions, as well as variations and unique interactions that represent additional ways for loops and helices to form interfaces. A number of sequence-structure correlations emerged, which may help in predicting structures of RNA from sequence in the future. The study improves our understanding of the types of loop-helix interactions that occur in nature, although it also points to the existence of additional loop-helix interaction motifs that await discovery.

## Materials and Methods

PDB files were downloaded from the Protein Data Bank (RNAs, >50 nts, X-ray crystal structures, prior to Jan. 1, 2011) and screened for redundancy. In most cases structures with greatest resolution were retained (Table S1). For ribosome structures, one large and one small subunit were retained for the *E. coli* ribosome (PDB ID’s 3OFO, 3OFR), as well as the large subunit of the archaebacteria *Haloarcula marismortui* (1VQO). Ribosomes of other species were deemed to mostly repeat the structural motifs of *E. coli*. In the cases of group I introns and RNase P, crystal structures exist for both full-length ribozymes at low resolution, and subdomains at higher resolution. In these cases, both structures were retained, and data from the lower resolution structures were considered only for the regions not present in the higher resolution structures (Tables S1, S2). Three SRPs were kept in spite of their similar structures, because their overall sequence identity was relatively low.

Loop-helix interactions were identified visually in the PDB files, and sub-PDB files were extracted using Swiss-PdbViewer (DeepView) [36] (http://www.expasy.org/spdbv/). Interactions were not retained when they spanned across unit cells of a crystal structure. Sixteen *E. coli* LSU RNA substructures were determined by superposition with Swiss-PdbViewer to be essentially identical to *Haloarcula* LSU structures, and were not included (Tables 1, 2). Four *E. coli* substructures differed somewhat from *Haloarcula* substructures (e.g. tetraloop vs. pentaloop) and were retained (Tables 1, 2). The extracted sub-PDB files are available in Structures S1. If opened with Swiss-PdbViewer the interactions are color-coded.

Structures were initially divided into four sets: 1) the GNRA-tetraloop/11-nt receptor configuration; 2) a tetraloop of any sequence interacting with a helix; 3) a loop of greater than four or fewer than four residues, but having a GNRA-tetraloop-like geometry and interacting with a helix; and 4) the remaining structures. Structures with GNRA/11-nt interactions were readily identified and superposed because of their similar sequences and structures. For set 2, similar structures were identified using Ensemble Cluster and Ensemble Match functions of the UCSF Chimera package [37] (http://www.cgl.ucsf.edu/chimera), which is from the Resource for Biocomputing, Visualization, and Informatics at the University of California, San Francisco (supported by NIH P41 RR001081). Pairwise superpositions by Ensemble Match were based on the backbone atoms of eight nucleotides (tetraloop and two base pairs; 96 atoms total), while groupings formed by Ensemble Cluster were based on RMSD values.

For sets 3 and 4, RNA loops were not of uniform length, making it difficult to judge which nucleotides should superimpose. To identify similar structures in these sets, the MatchMaker function of Chimera was used, which uses one point per nucleotide, and refines superposition by iterative cycles that exclude outlier nucleotides. After identifying similar structures within sets 3 and 4, all members of the two sets were compared with each structure in sets 1 and 2. Structures in sets 3 and 4 that were superposed with structures in sets 1 or 2 by MatchMaker were moved to those sets, and were tagged NTL (non-tetraloop). In addition, structures in set 2 that did not adopt the GNRA geometry were moved to sets 3 or 4. Throughout the process we visually compared structures, and similarities missed by MatchMaker were corrected by removing extraneous nucleotides and re-analyzing with MatchMaker.

To judge the quality of the selected substructures, the models and electron density maps were visualized using the program Coot [38]. Electron density maps were downloaded from the Electron Density Server [39], if available. For the other cases where structure factors were available from the Protein Data Bank, electron density maps were calculated using either PHENIX [40] or programs from the CCP4 package [41]. Although Coot can calculate the residue density fit, the score is based on the average electron density at the centers of the atoms in the model and does not consider electron density nearby, which may better fit the residue. For this reason, a qualitative judgment was made of the agreement between the electron density and residues of the substructures.

Leontis-Westhof notation of base-base interactions [26], [42] was extracted with S2S (Sequence to Structure) [43]. Molecular graphics images were produced using the UCSF Chimera package.

## Supporting Information

Figure S1 **Secondary structures and front and back stereo views of all interactions in this study.** Gray indicates nucleotides that do not belong to the loop-helix interaction. Secondary structures are drawn in the Leontis-Westhof notation (see Figure 1C). A) Class I interactions B) Class II interactions B1) Class II/Subclass 1 interactions Pt1) Class II/Subclass 1.1.1 Pt2) Class II/Subclass 1.1.2 Pt3) Class II/Subclass 1.1 (Indiv) Pt4) Class II/Subclass 1 (Indiv) Pt5) Class II/Subclass 1 (NTL) B2) Class II/Subclass 2 and 2 (NTL) B3) Class II/Subclass 3 and 3 (NTL) B4) Class II/Subclass 4 and 4 (NTL) B5) Class II/Subclass 5 C) Class III interactions C1) Class III/Subclass 1 C2) Class III/Indiv D) Class IV interactions D1) Class IV/Subclass 1 D2) Class IV/Indiv: single base inserted into receptor helix D3) Class IV/Indiv: surface formed by splayed nucleotides D4) Class IV/Indiv: other unique interactions D5) Class IV/Indiv: single base interactions
(PDF)

Figure S2 **Structures of Class II/Subclasses 1.1 (Indiv) and 1 (Indiv).** A) Consensus secondary structure of Class II/Subclass 1.1 (Indiv). B) Two views of an overlay of eight 1.1 (Indiv) structures (purple backbones) and a reference structure of Subclass 1.1.1 (3IGI:90–93) (yellow backbone and bases). C) Secondary structures of the four Subclass 1 (Indiv) structures and the reference 1.1.1 structure (3IGI:90–93) (yellow). Gray bases are not shown in the three-dimensional depictions in panels D, E and F. D,E,F) Superposition of Class II/Subclass 1 (Indiv) structures based on the receptor backbone atoms. Three stereoviews are shown, color-coded as indicated in Panel C.
(JPG)

Figure S3 **Structures of Class II/Subclass 1 (NTL) structures.** A) Overlay of thirteen Subclass I (NTL) structures and a 1.1.1 reference (3IGI:90–93; yellow). Superposition is based on the backbones of 4 nucleotides of the receptor and either one or two nucleotides from the loop (T2, T3 in the case of tetraloops). Green and blue indicate the two loop nucleotides having different backbone geometries but analogous base positions as other structures in the subclass (see Panel B). B) Secondary structures of all Subclass 1 (NTL) loops, with gray indicating nucleotides not adopting the GNRA tetraloop geometry, and not shown in Panel A.
(JPG)

Figure S4 **Secondary and tertiary structures of Class II Subclasses 2, 3 and 4.** A) Two views of the overlay of five structures of Class II/Subclass 2. Blue nucleotides represent the three tetraloop-receptor structures, while pink and green nucleotides represent the NTL-receptor structures (see panels B, C, D). B) Consensus secondary structure for the five structures in Subclass 2. C, D) Secondary structures for Subclass 2 (NTL) loops. E) Two views of the overlay of four structures of Class II/Subclass 3. Blue nucleotides represent the three tetraloop-receptor structures, while green nucleotides represent the NTL-receptor structure. F) Consensus secondary structure for the four structures in Subclass 3. G) Secondary structure for the NTL loop in Subclass 3. H) Two views of an overlay of Class II/Subclass 4 structures. Blue nucleotides represent the tetraloop-receptor structure, while green nucleotides represent the NTL interaction. I) Consensus secondary structure for Subclass 4. J) Secondary structure for the NTL loop of Class II/Subclass 4.
(JPG)

Figure S5 **Superposition of representative Class II structures from all five classes.** A) Secondary structures of representatives from each class (Subclass 1, 3IGI:90–93 (yellow); Subclass 2, 1VQO:691–694 (green); Subclass 3, 1MFQ:147–150 (orange); Subclass 4∶3OFO:159–162 (pink); Subclass 5∶3IGI:369–372 (white)). B, C, D) Three stereoviews of the superposition, based on the backbone atoms of the tetraloop, and color-coded as in panel A.
(JPG)

Figure S6 **Secondary and tertiary structures of Classes III and IV.** A) Consensus secondary structure for Class III/Subclass 1. B) Two views of the superposition of VQO:2837–2843 (green) and 3OFR:642–646 (purple). C) Consensus secondary structure for Class IV/Subclass 1. D) Two views of the superposition of 2A64∶98–107 (green) and 3OFR:124–127 (yellow). Gray nucleotides are loop nucleotides that do not interact with the receptor.
(JPG)

Figure S7 **Comparison of structures in Classes II and III.** A, B, C) Three stereoviews of the overlay of four representatives from Class II/Subclasses I–IV and all five structures from Class III. Superposition is based on the backbone atoms of the four receptor nucleotides. D) Secondary structure depictions and color-coding for the three-dimensional depictions above.
(JPG)

Figure S8 **Superposition of GNRA loop structures.** A. Superposition of 30 GNRA tetraloop structures. Green (Class I) 1NBS:205–208, 1U6B:24–27, 1U6B:189–192, 2R8S:150–153; Pink (Class II Subclass 1.1.1) 2Z75∶114–117, 3IGI:90–93, 3OK7∶93–96, 3OK7∶285–288, 3OFO:1077–1080, 3OFO:1266–1269, 3OFR:2857–2860; Orange, Class II Subclass 1.1.2, 1U9S:205–208, 3MXH:32–35, 1VQO:1629–1632, 1VQO:1863–1866; Gold, Class II Subclass 1.1 (Indiv) 1Y0Q:22–25, 1Y0Q:205–208, 1VQO:469–4723, 1VQO:577–580, 1VQO:1327–13303, 1VQO:2630–26333, 3OFO:1013–1016, 3OFR:1807–1810; Purple (Class II Subclass 1 (Indiv)) 1X8W:323–326, 3OFO:898–901, 3OFO:1516–1519; Light blue (Class II Subclass 2) 1VQO:691–694, 3OFR:630–633, 3OFR:1364–1367; Red (Class II Subclass 4) 3OFO:159–162; Burnt orange (Class IV Subclass 1) 3OFR:124–127. B. Superposition of five non-GNRA sequences that assume GNRA tetraloop structures. Gold (Class II Subclass I (Indiv)) (UCAA sequence) 1VQO:734–737; Blue (Class II Subclass 3) (GNAG sequence) 1LNG:163–166, 1MFQ:147–150, 3KTW:164–167; Pink (Class II Subclass 5) (GAAC sequence) 3IGI: 369–372.
(JPG)

Structures S1 **This file contains sub-PDB files for all individual interactions in this study.** The file name corresponds to the PDB ID with start and end positions of the loop. If opened with Swiss-PdbViewer the interactions are color-coded.
(RAR)

Table S1 **Compiled list of nonredundant crystal structures for RNAs greater than 50 nucleotides and with resolution greater than 4 Å (as of Dec. 31, 2010).**
(DOC)

Table S2 **List of PDB substructures containing RNA loop-helix interactions (derived from Table S1).**
(DOC)

Table S3 **Sequences of tetraloops and receptors arranged according to structural subclasses.**
(DOC)
