# Supplementary material for: Structural Variation and Uniformity among Tetraloop-Receptor Interactions and Other Loop-Helix Interactions in RNA Crystal Structures
Source: PLoS One. 2012 Nov 9;7(11):e49225. doi: 10.1371/journal.pone.0049225 (PMC3494683; doi:10.1371/journal.pone.0049225)
Supplement: Figure S1 — Secondary structures and front and back stereo views of all interactions in this study. Gray indicates nucleotides that do not belong to the loop-helix interaction. Secondary structures are drawn in the Leontis-Westhof notation (see Figure 1C). A) Class I interactions B) Class II interactions B1) Class II/Subclass 1 interactions Pt1) Class II/Subclass 1.1.1 Pt2) Class II/Subclass 1.1.2 Pt3) Class II/Subclass 1.1 (Indiv) Pt4) Class II/Subclass 1 (Indiv) Pt5) Class II/Subclass 1 (NTL) B2) Class II/Subclass 2 and 2 (NTL) B3) Class II/Subclass 3 and 3 (NTL) B4) Class II/Subclass 4 and 4 (NTL) B5) Class II/Subclass 5 C) Class III interactions C1) Class III/Subclass 1 C2) Class III/Indiv D) Class IV interactions D1) Class IV/Subclass 1 D2) Class IV/Indiv: single base inserted into receptor helix D3) Class IV/Indiv: surface formed by splayed nucleotides D4) Class IV/Indiv: other unique interactions D5) Class IV/Indiv: single base interactions (PDF) [file pone.0049225.s001.pdf]

A

Class I

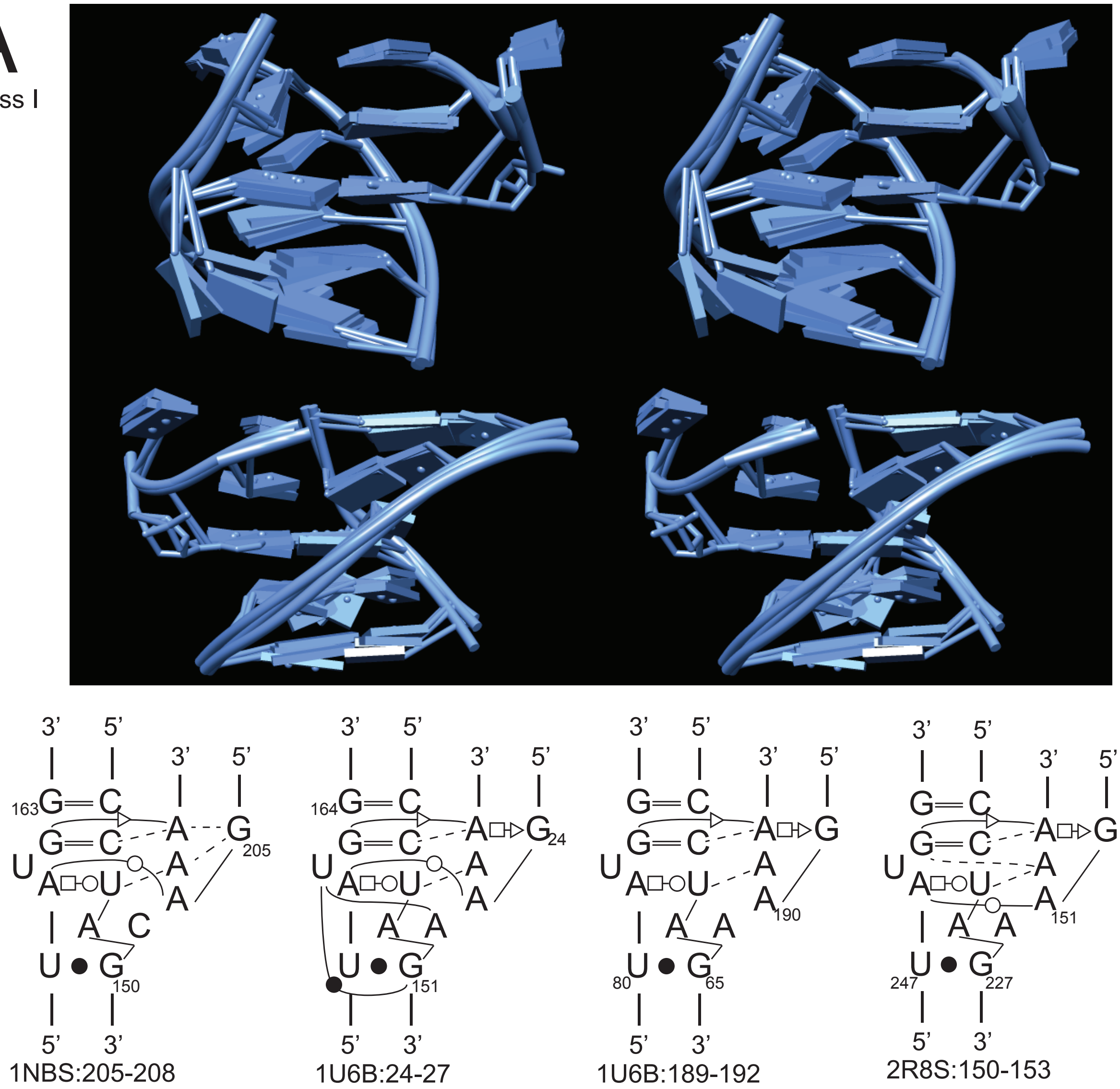

B1 (Pt1 #1)

Class II/Subclass 1.1.1

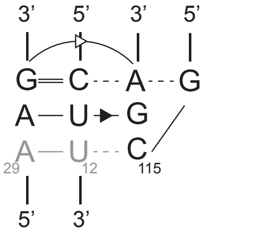

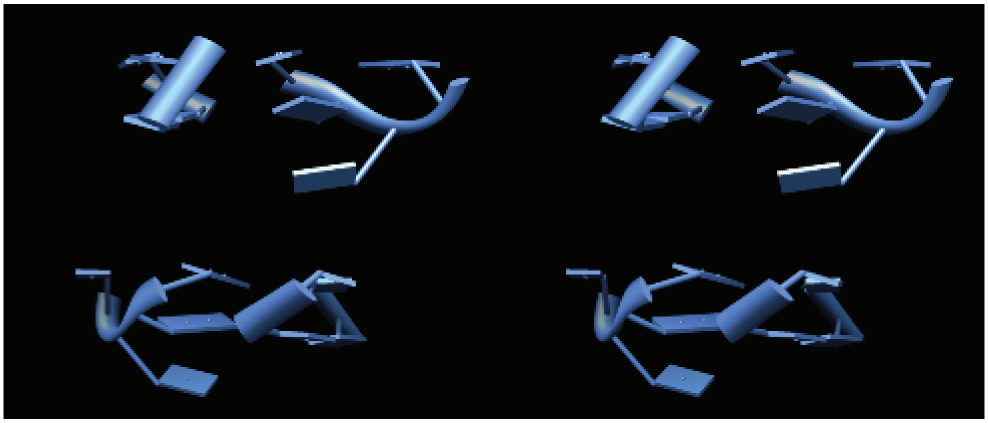

2Z75:114-117

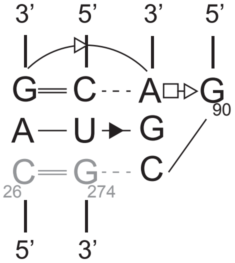

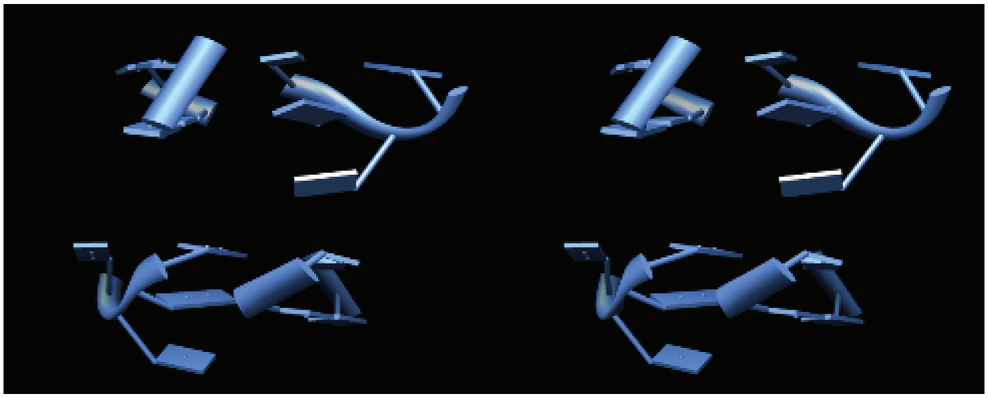

3IGI:90-93

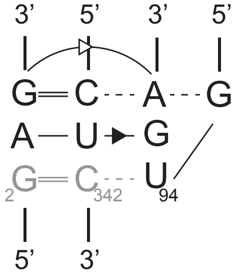

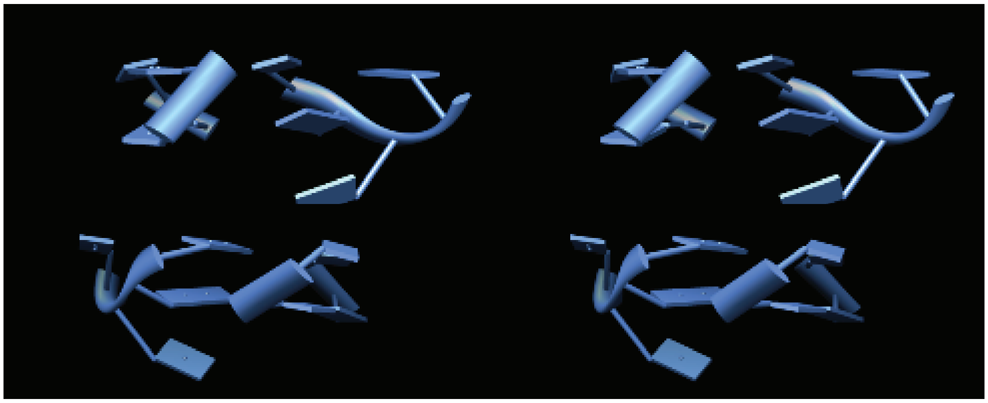

3OK7:93-96

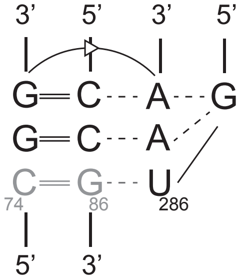

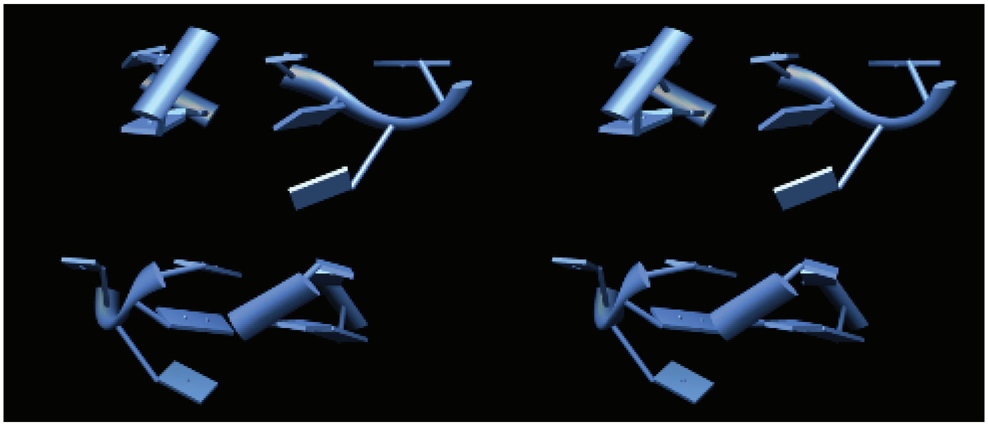

3OK7:285-288

B1 (Pt1 #2)

Class II/Subclass 1.1.1

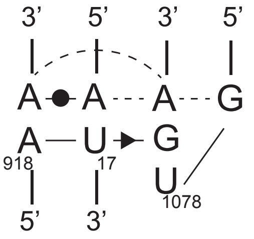

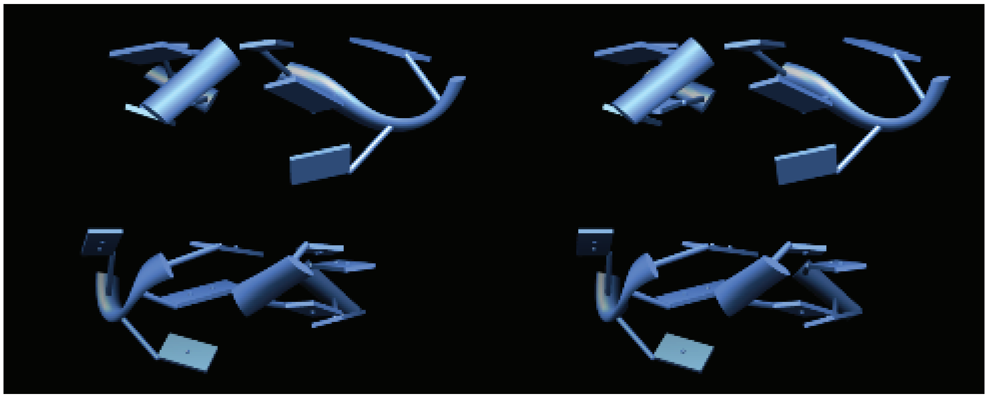

3OFO:1077-1080

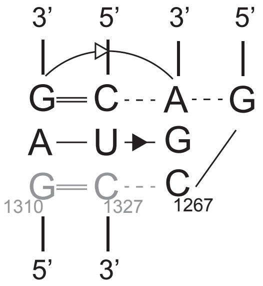

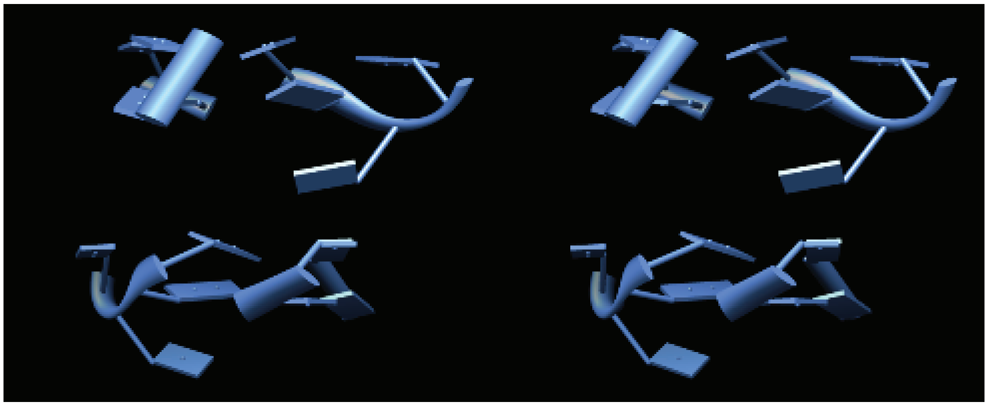

3OFO:1266-1269

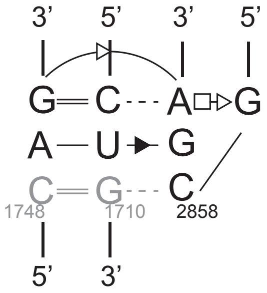

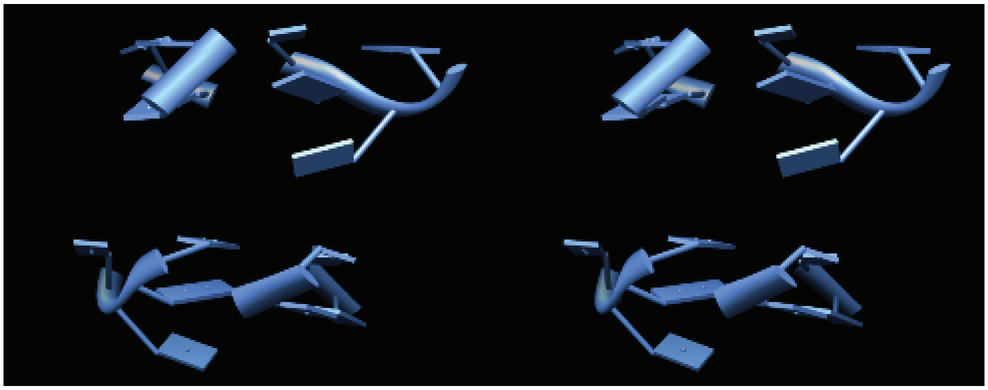

3OFR:2857-2860

B1 (Pt2)

Class II/Subclass 1.1.2

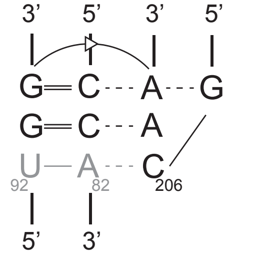

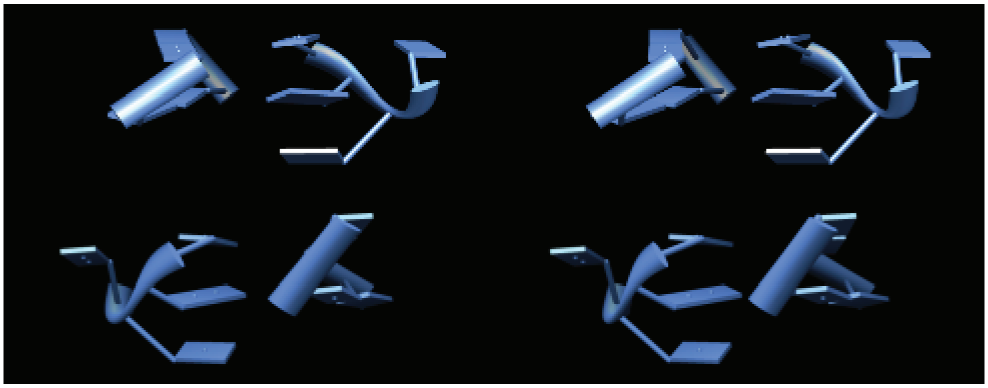

1U9S:205-208

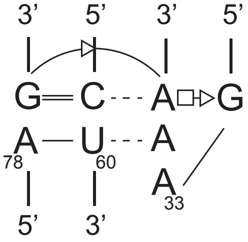

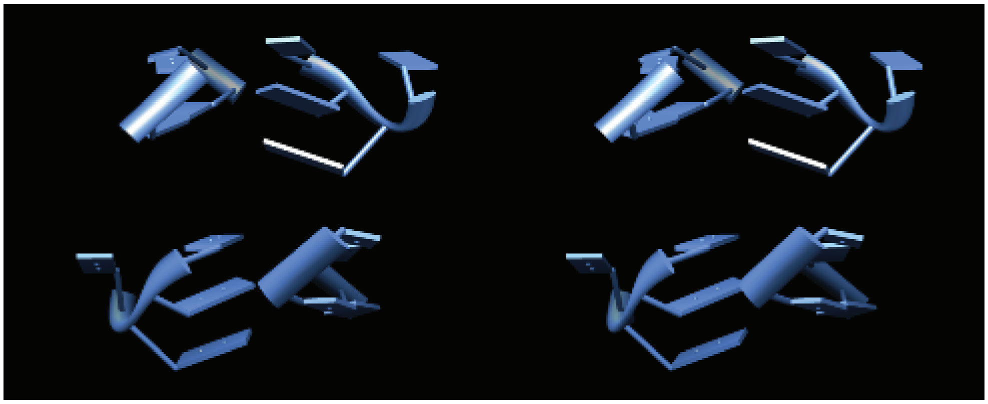

3MXH:32-35

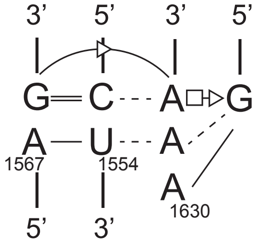

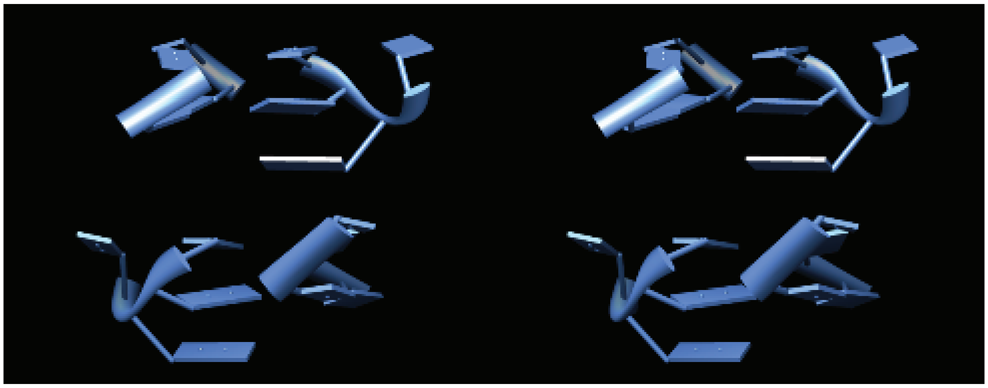

1VQO:1629-1632

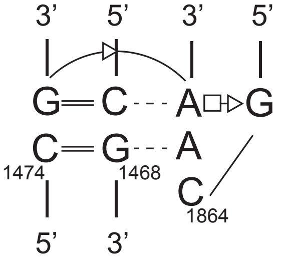

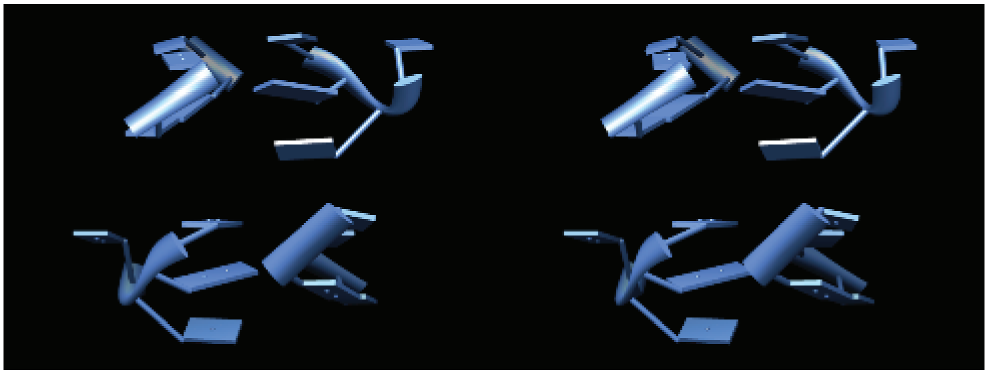

1VQO:1863-1866

B1 (Pt3 #1)

Class II/Subclass 1.1
(Individual)

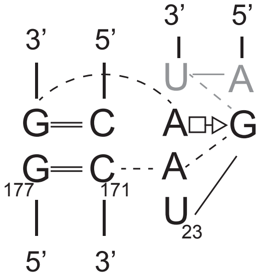

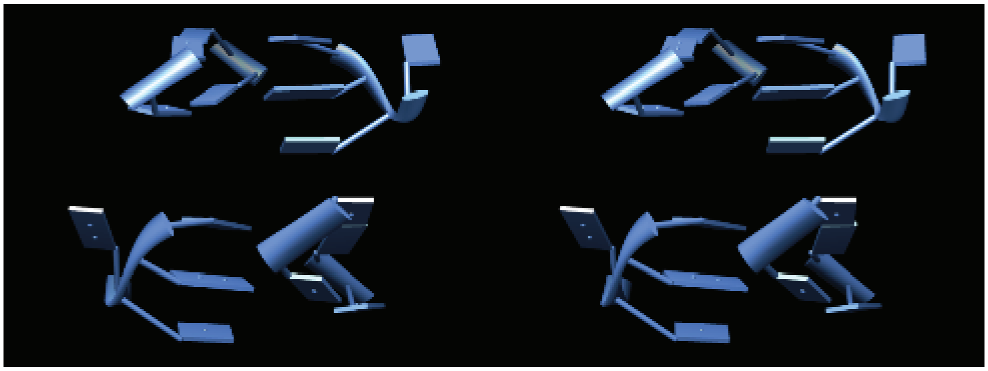

1Y0Q:22-25

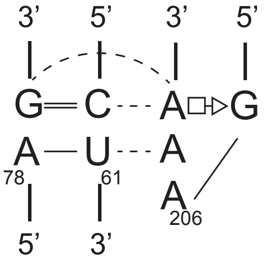

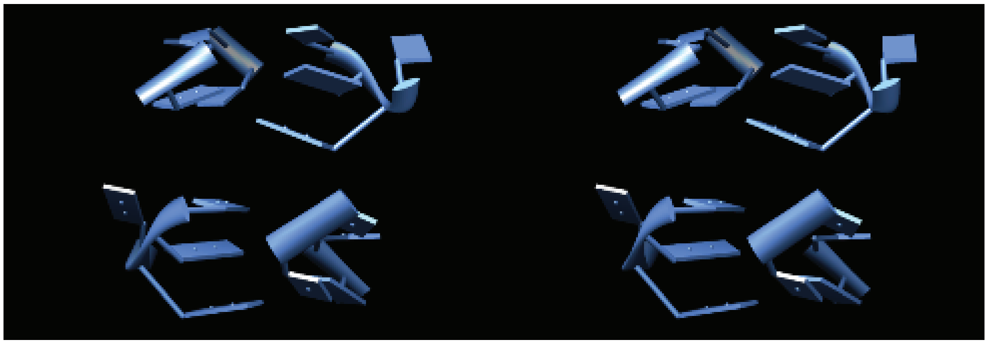

1Y0Q:205-208

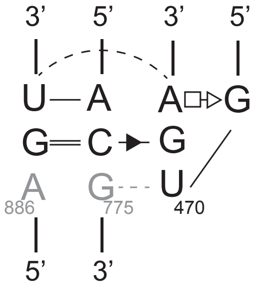

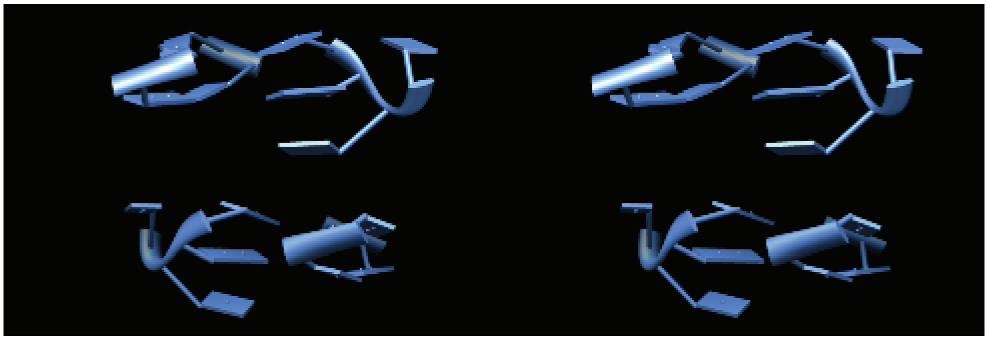

1VQO:469-472

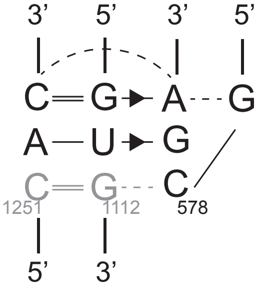

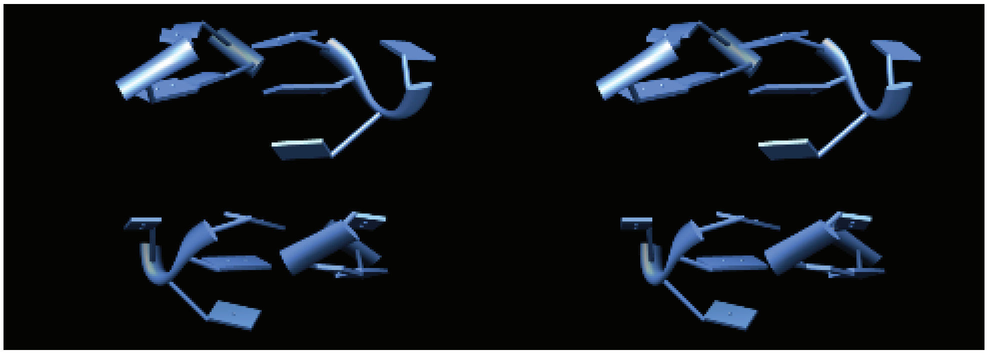

1VQO:577-580

B1 (Pt3 #2)

Class II/Subclass 1.1
(Individual)

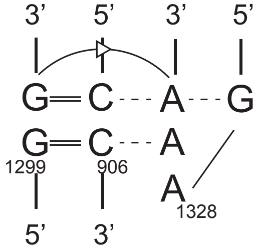

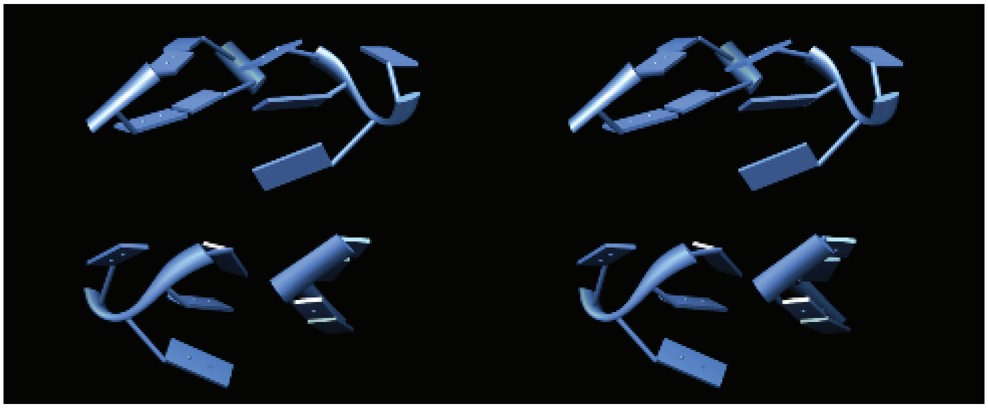

1VQO:1327-1330

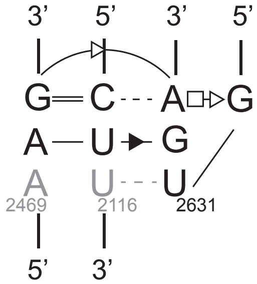

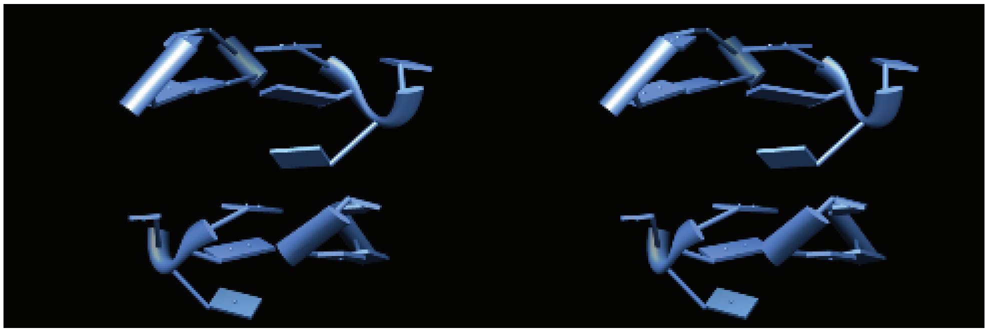

1VQO:2630-2633

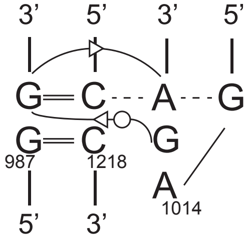

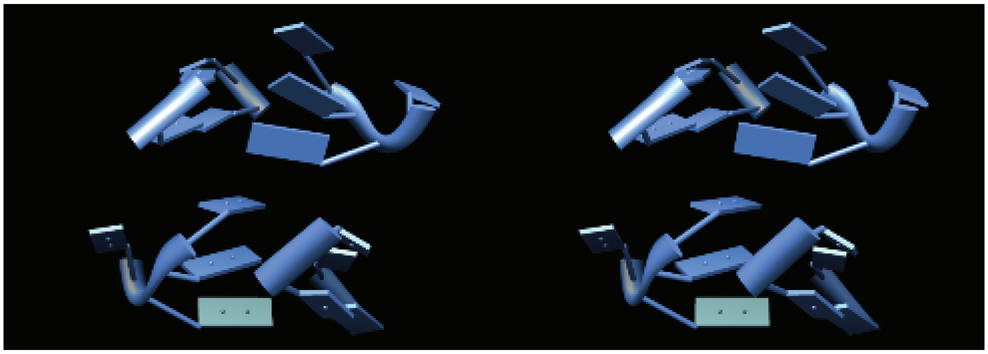

3OFO:1013-1016

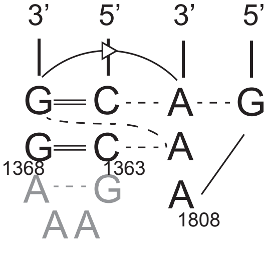

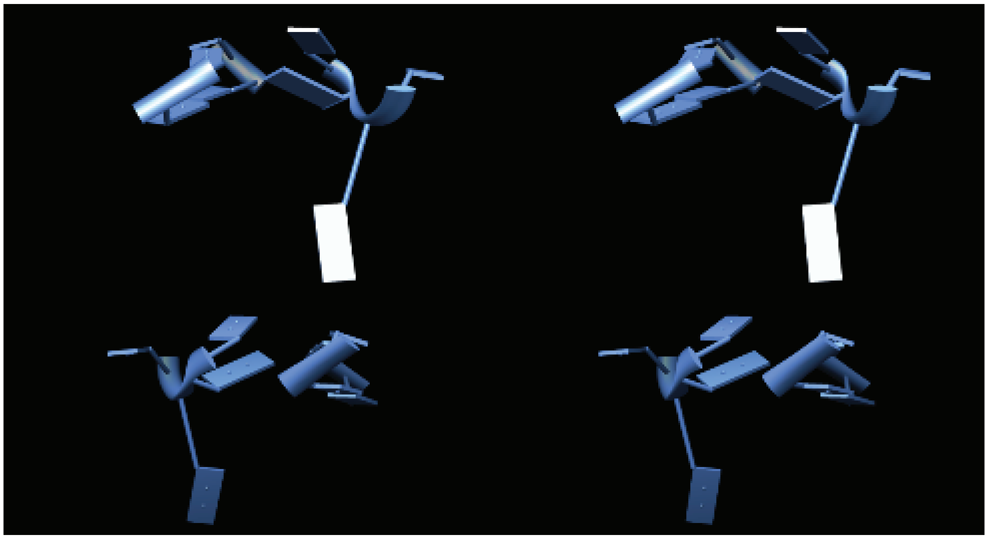

3OFR:1807-1810

B1 (Pt4)

Class II/Subclass 1
(Individual)

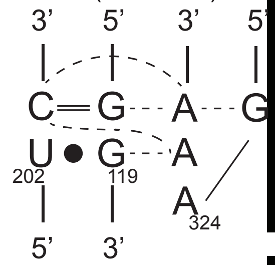

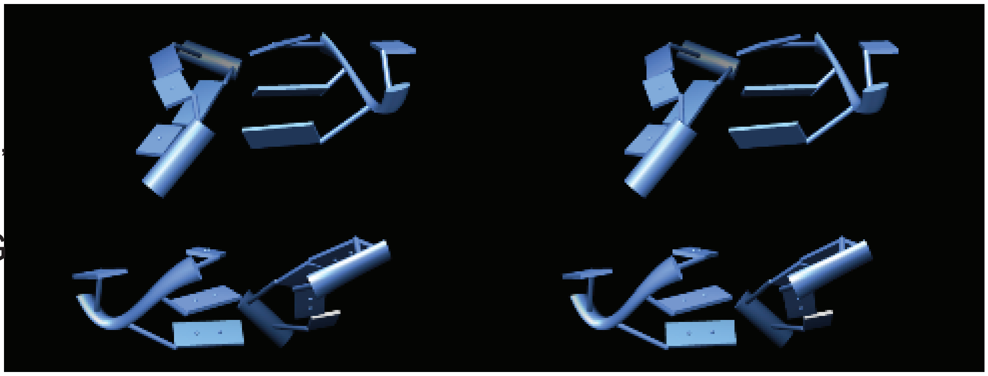

1X8W:323-326

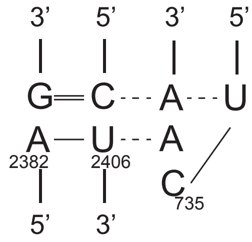

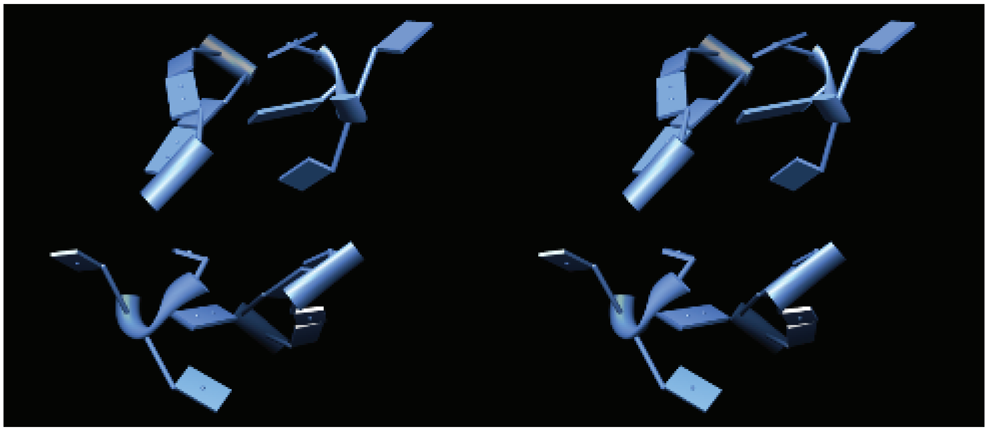

1VQO:734-737

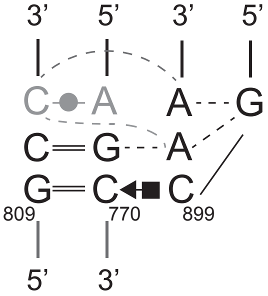

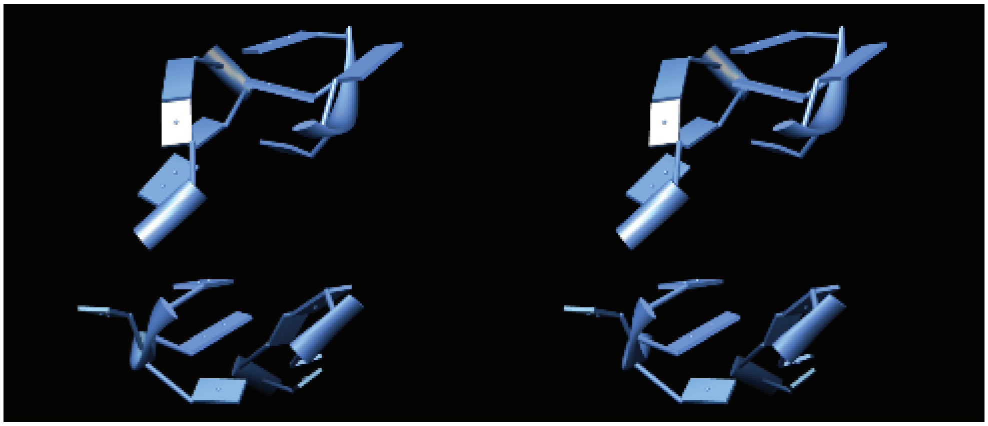

3OFO:898-901

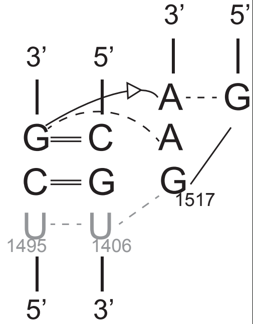

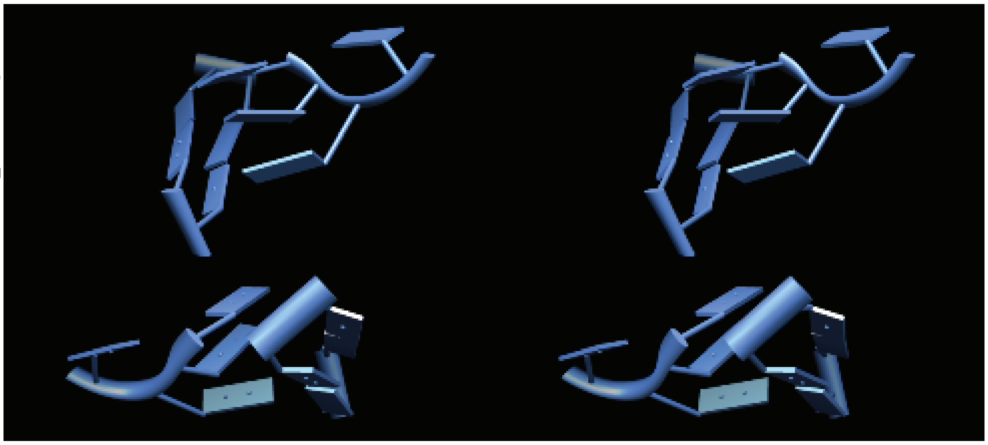

3OFO:1516-1519

B1 (Pt5 #1)

Class II/Subclass1 (NTL)

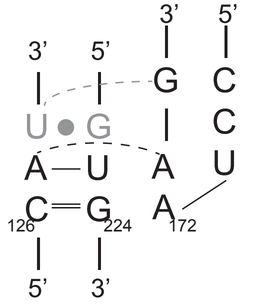

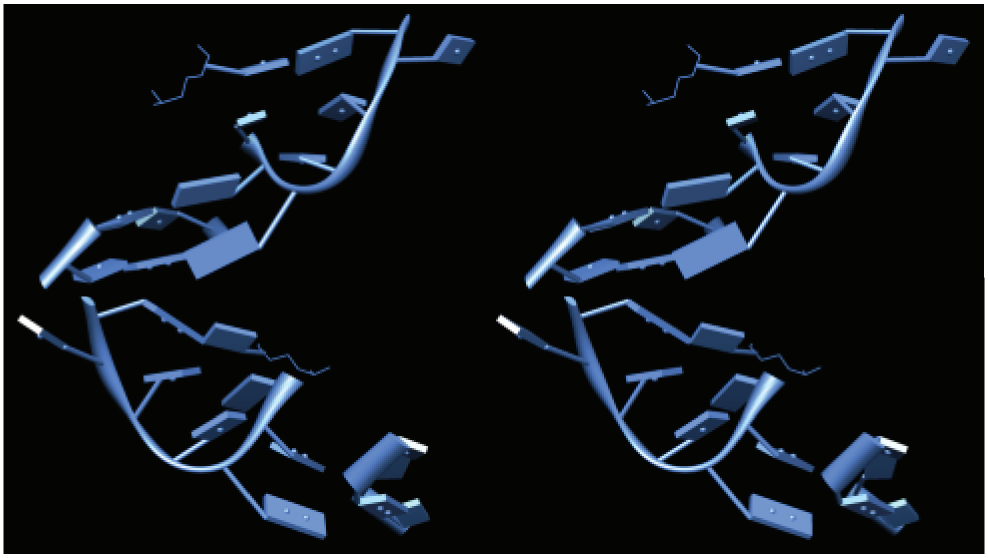

1MFQ:169-174

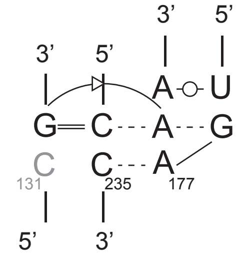

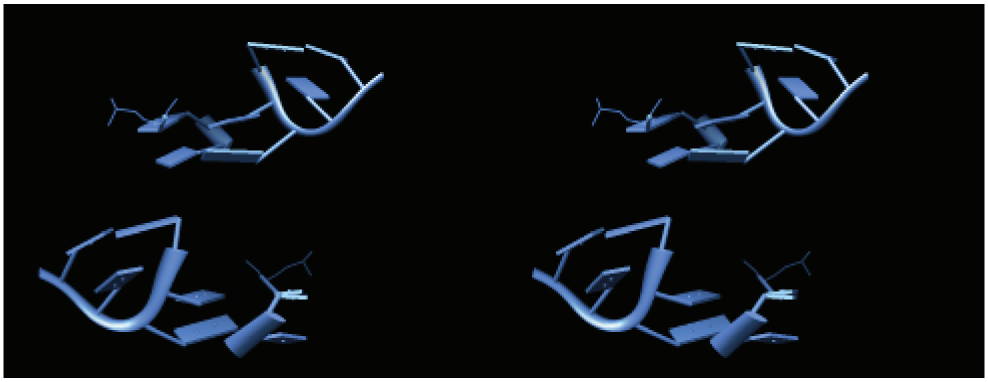

1NBS:175-179

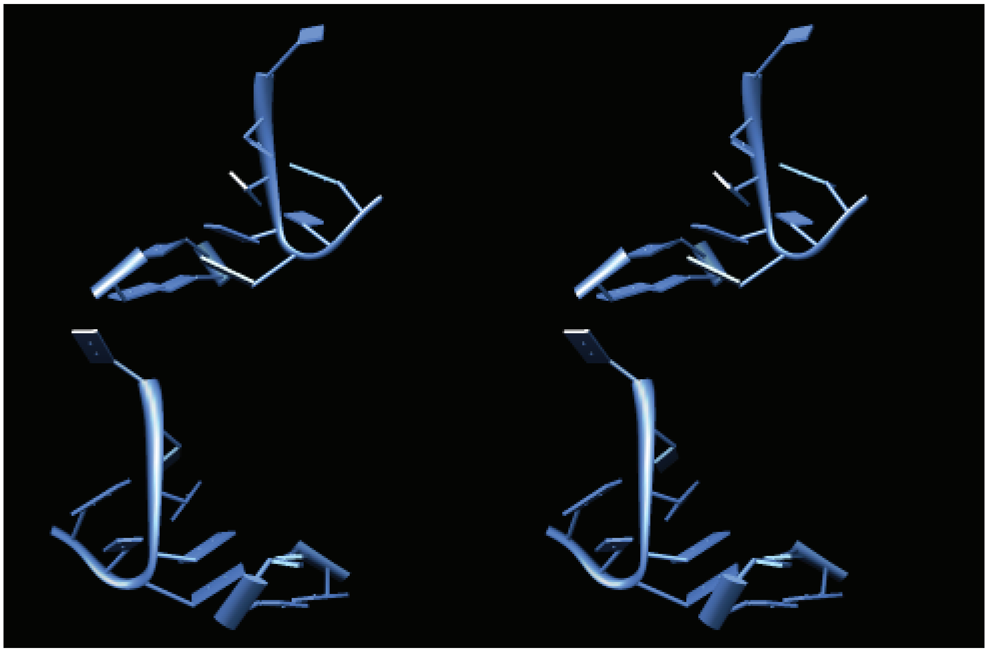

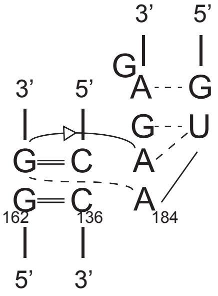

1U9S:182-188

B1 (Pt5 #2)

Class II/Subclass1 (NTL)

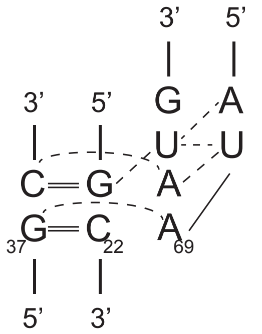

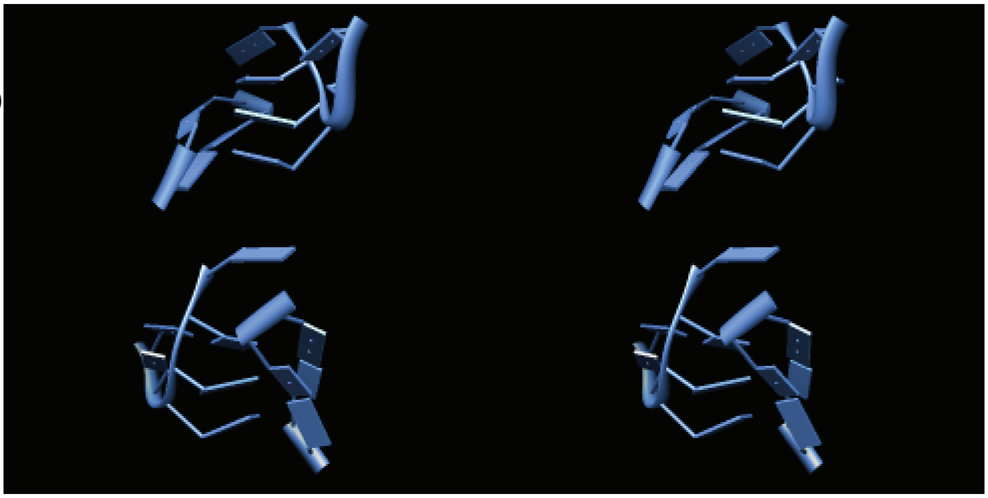

2GDI:67-72

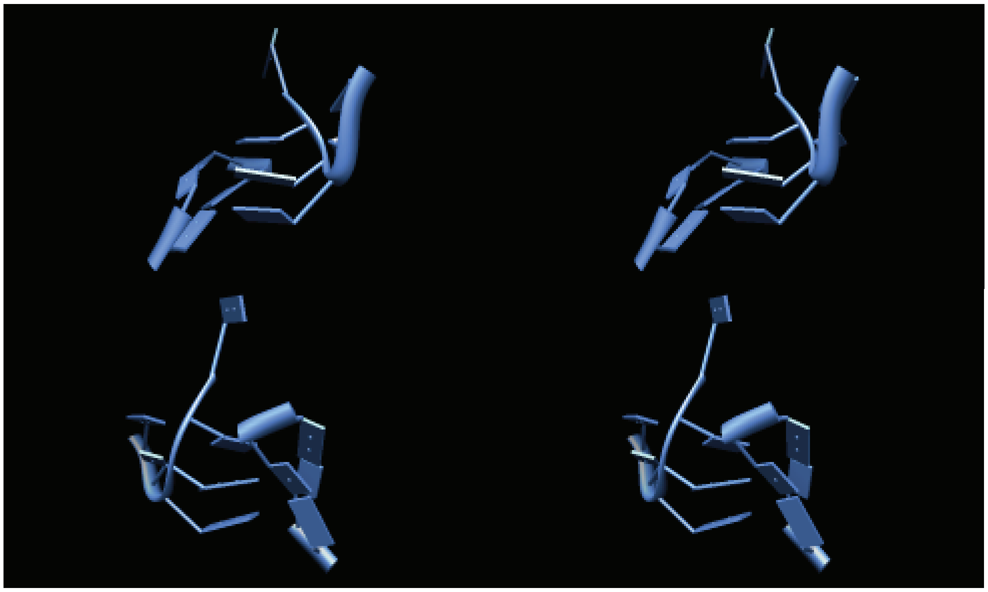

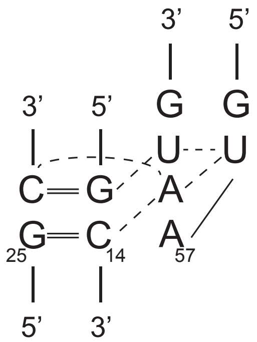

3D2V:55-60

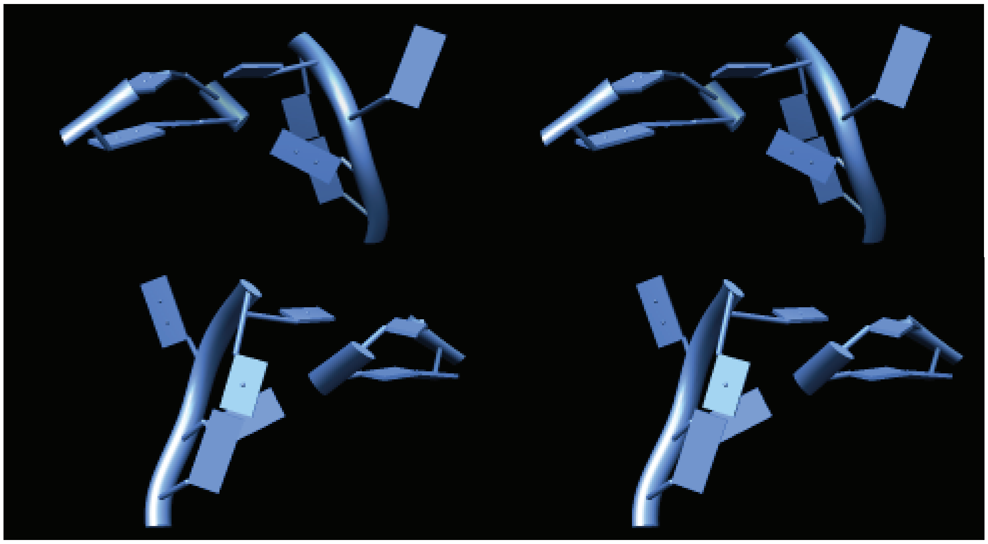

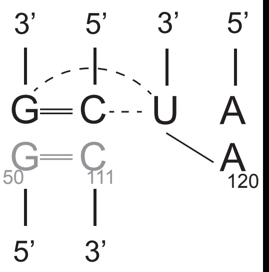

1VQO:119-121

B1 (Pt5 #3)

Class II/Subclass1 (NTL)

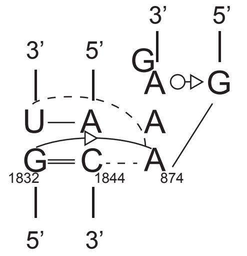

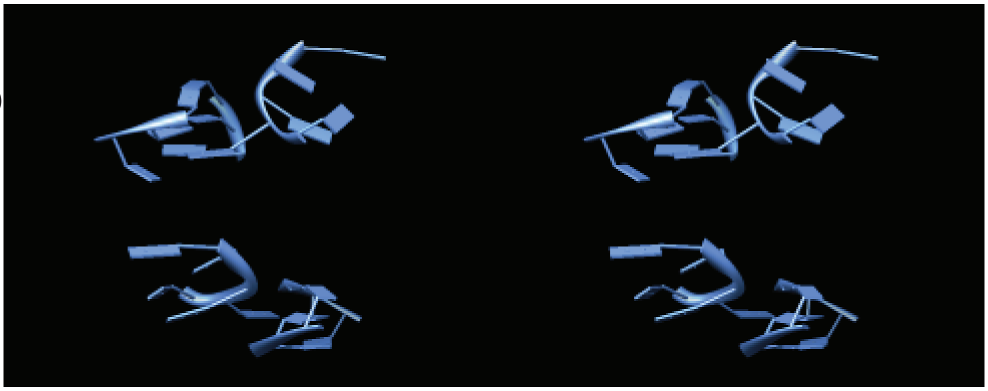

1VQO:873-877

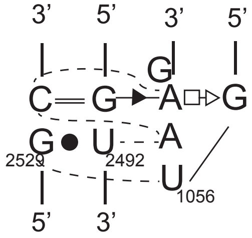

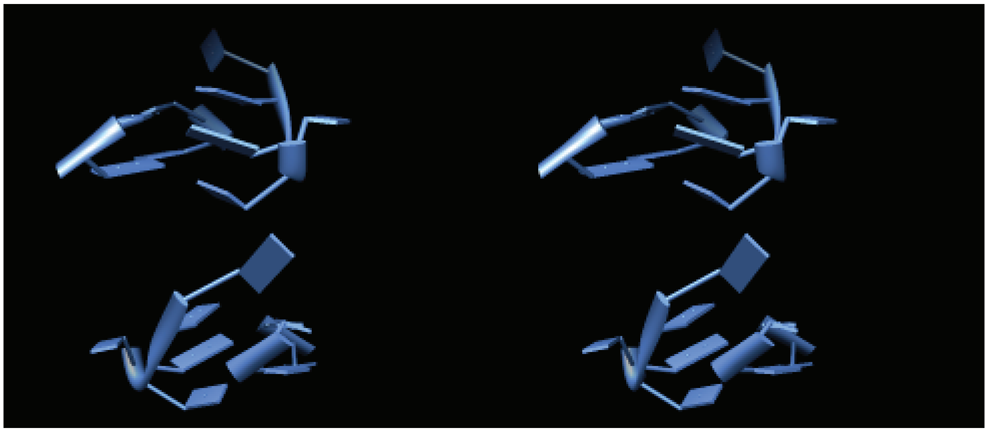

1VQO:1055-1059

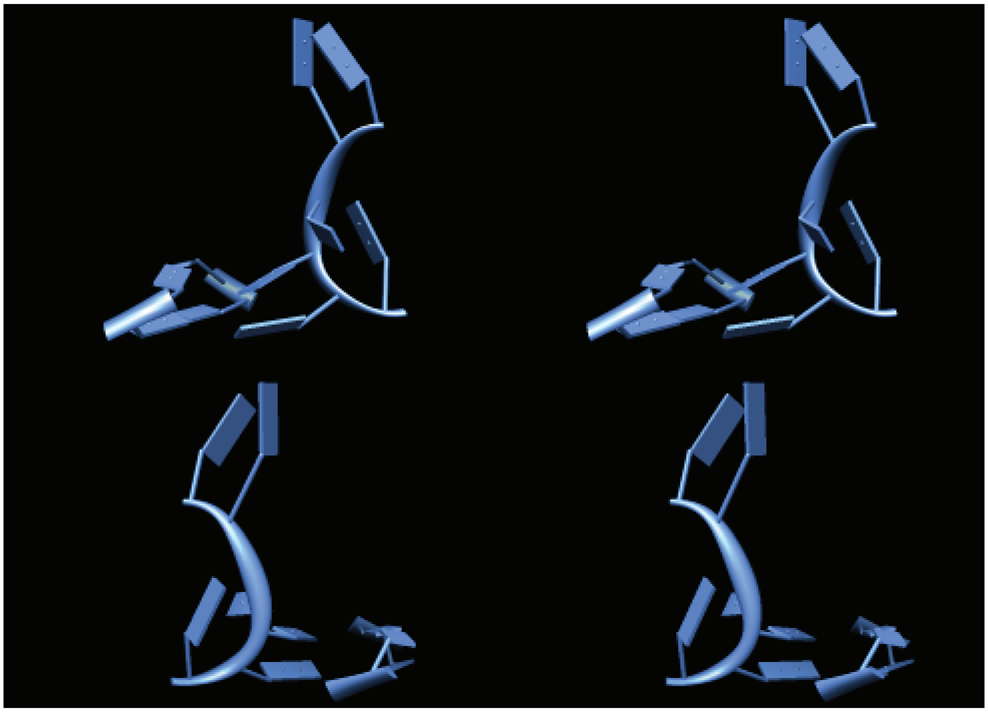

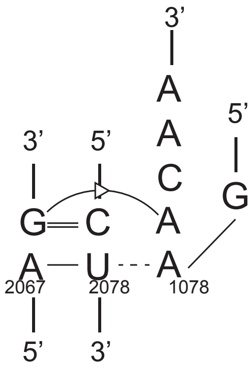

1VQO:1077-1082

B1 (Pt5 #4)

Class II/Subclass1 (NTL)

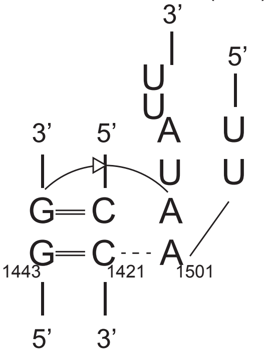

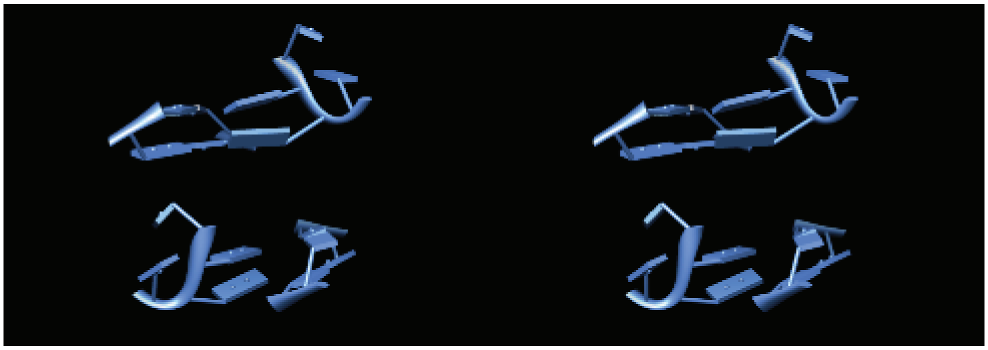

1VQO:1499-1506

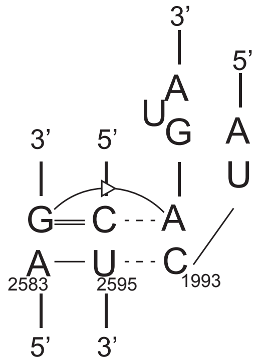

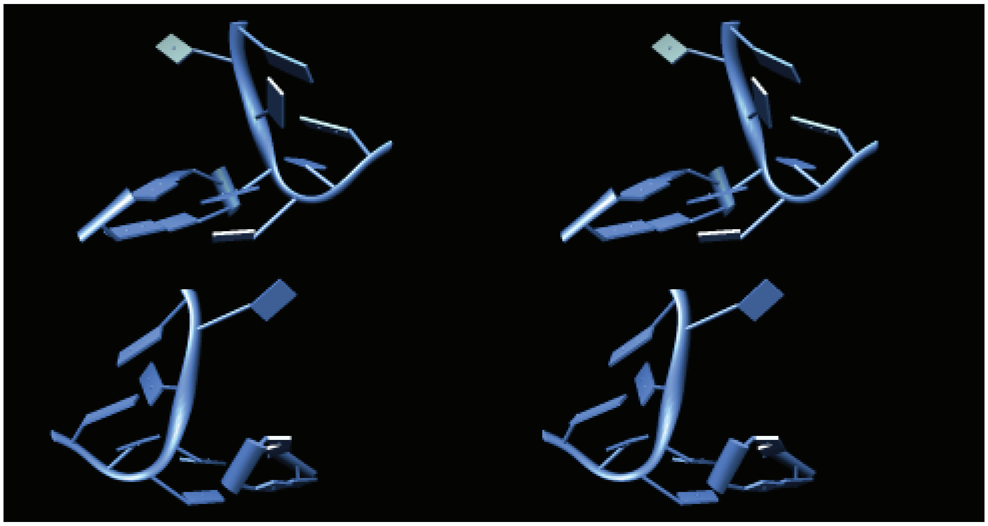

1VQO:1991-1997

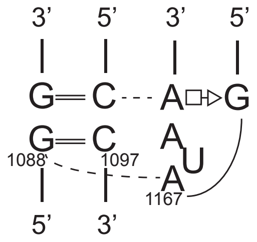

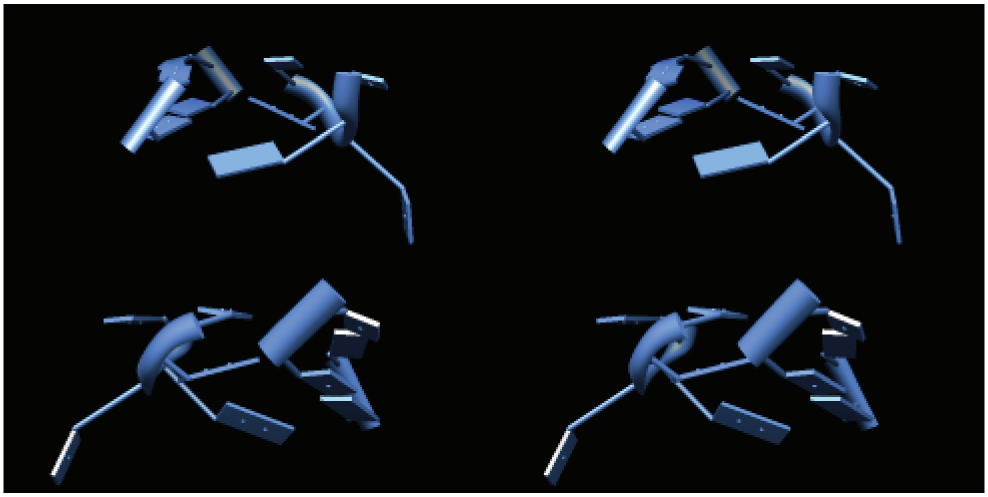

3OFO:1166-1170

B1 (Pt5 #5)

Class II/Subclass1 (NTL)

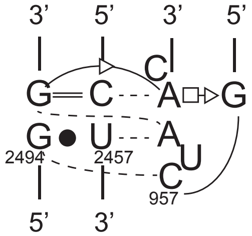

3OFR:956-961

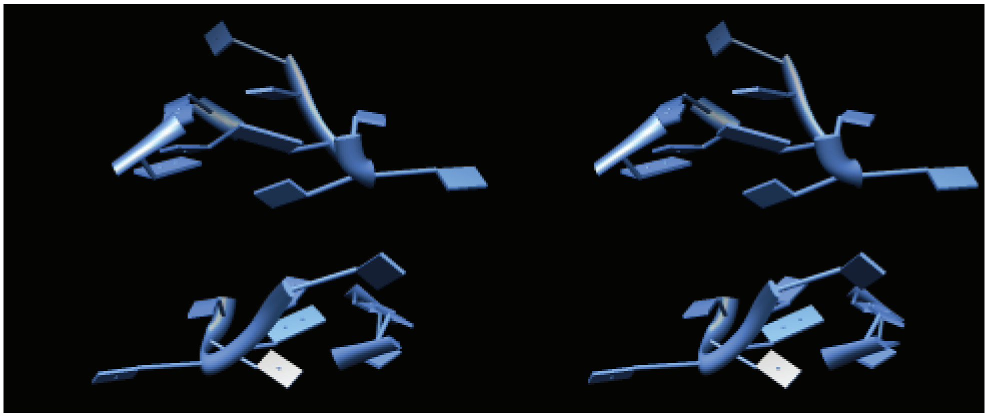

B2(#1)

Class II/Subclass 2

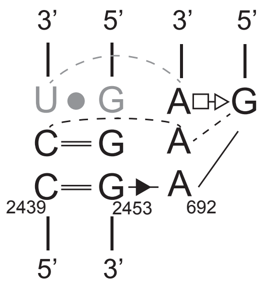

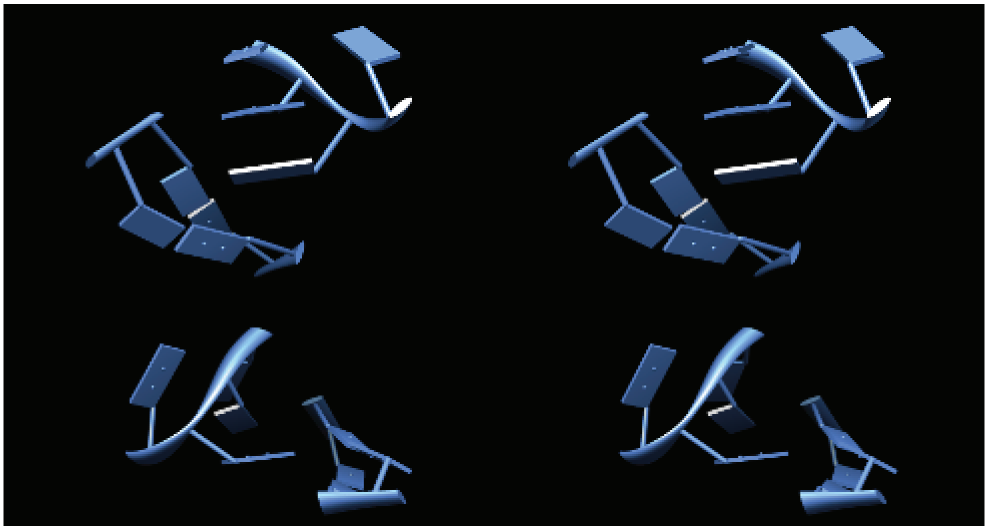

1VQO:691-694

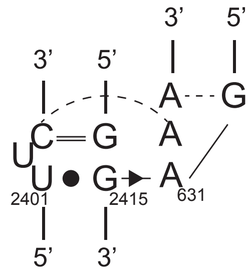

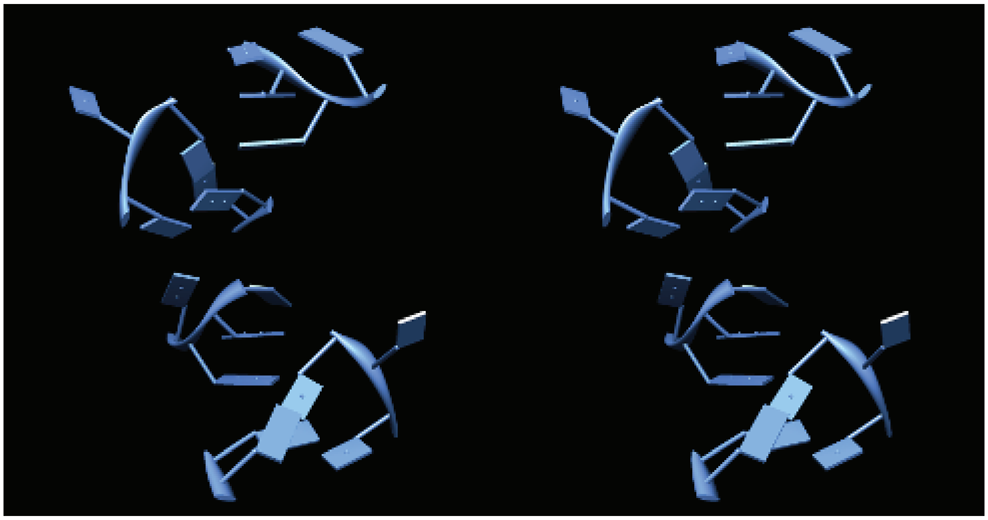

3OFR:630-633

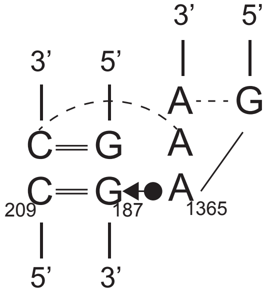

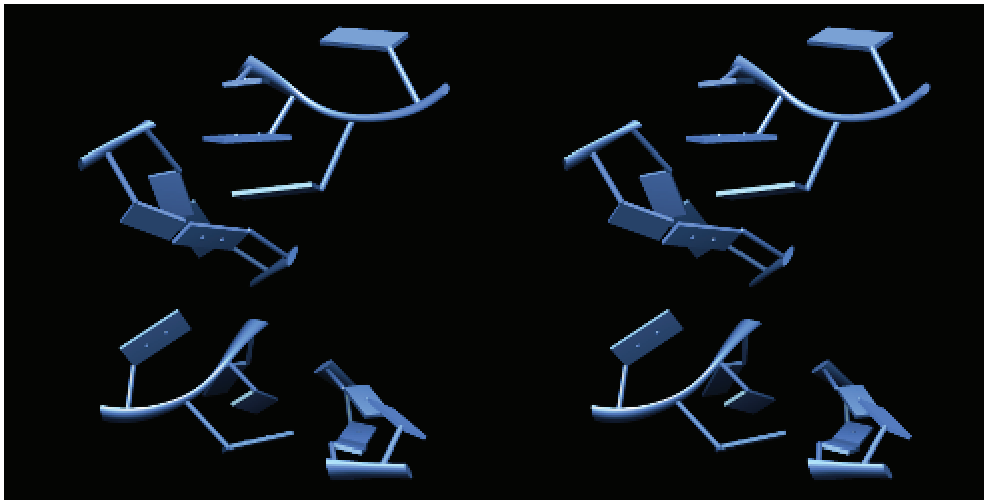

3OFR:1364-1367

B2(#2)

Class II/Subclass 2
(NTL)

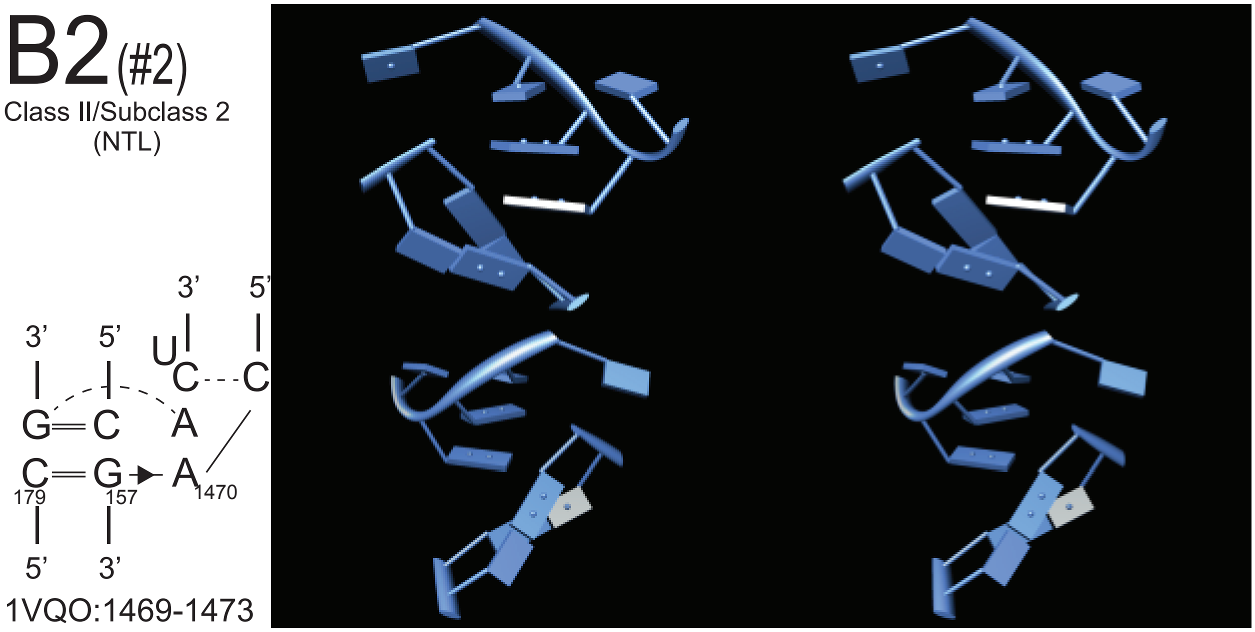

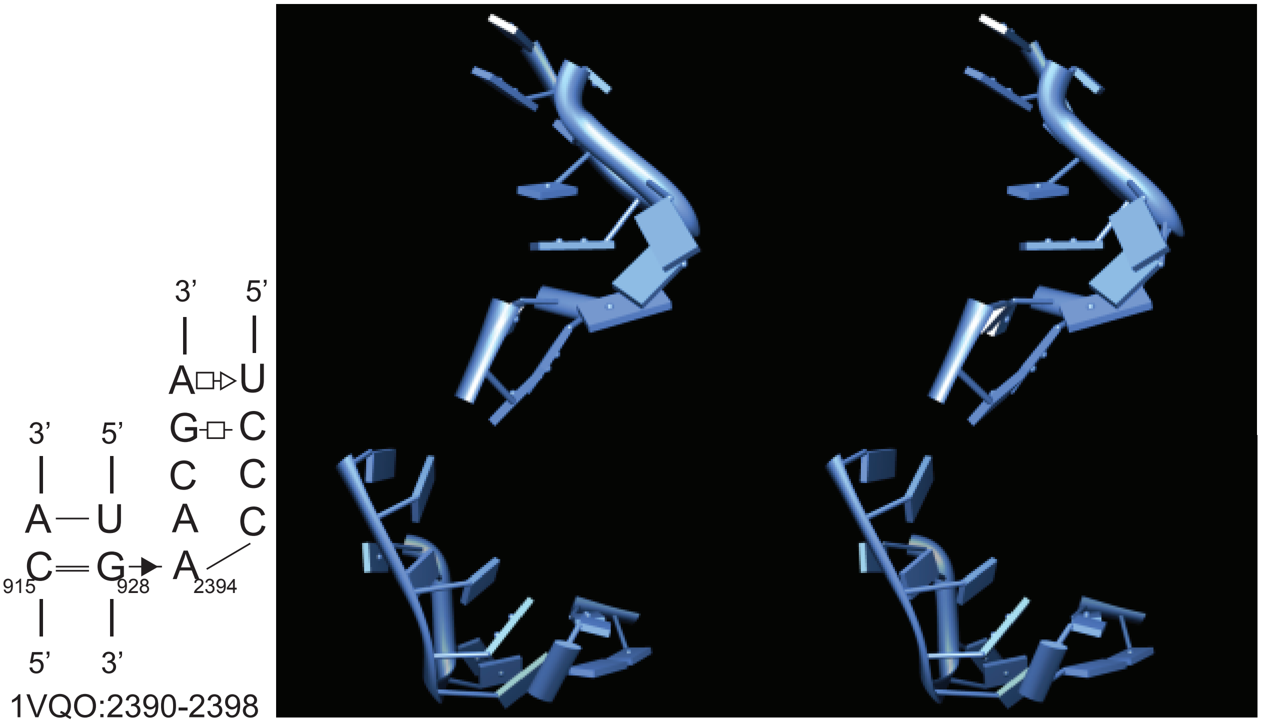

B3

Class II/Subclass 3

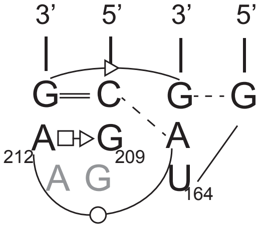

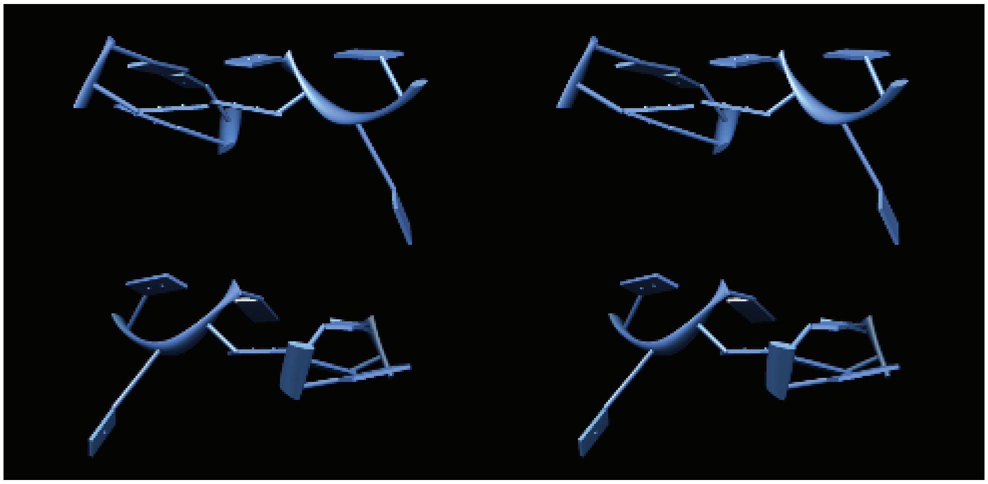

1LNG:163-166

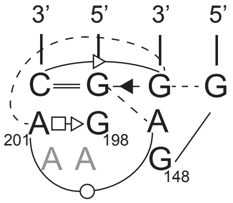

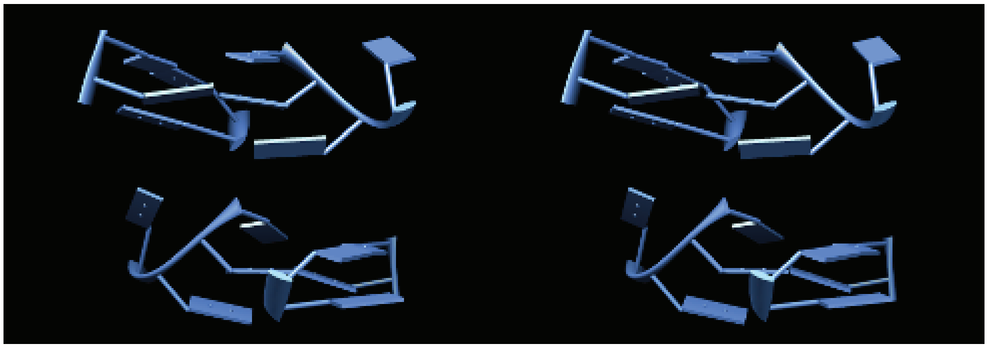

1MFQ:147-150

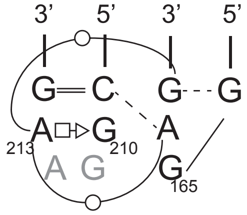

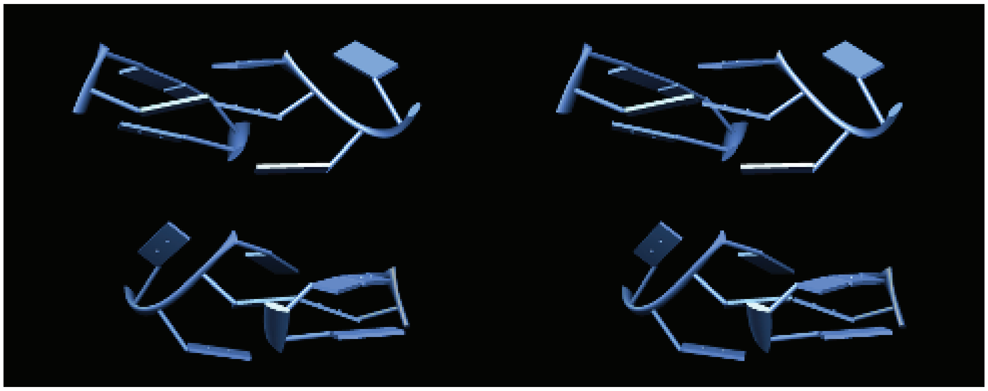

3KTW:164-167

Class II/Subclass 3
(NTL)

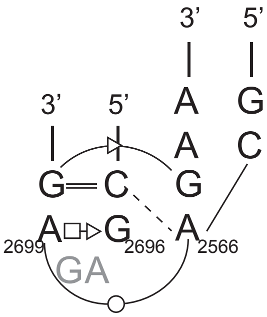

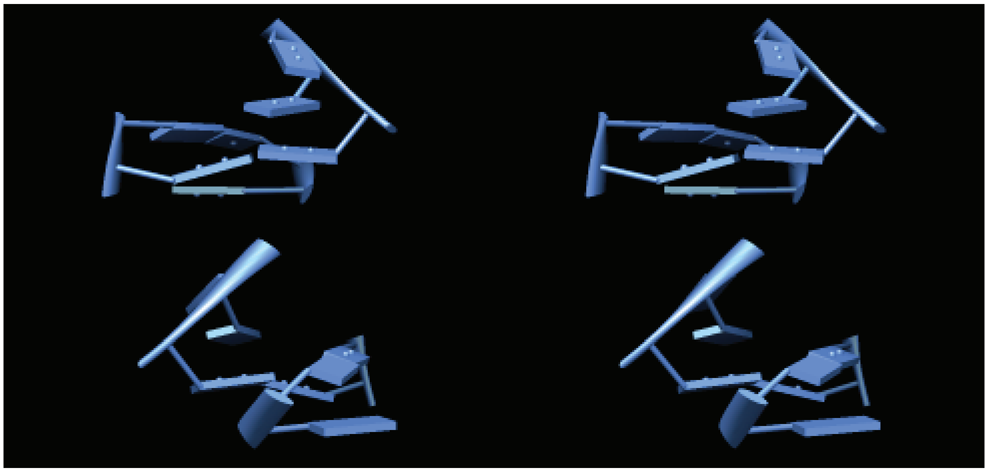

1VQO:2564-2569

B4

Class II/Subclass 4

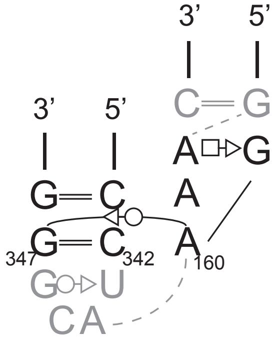

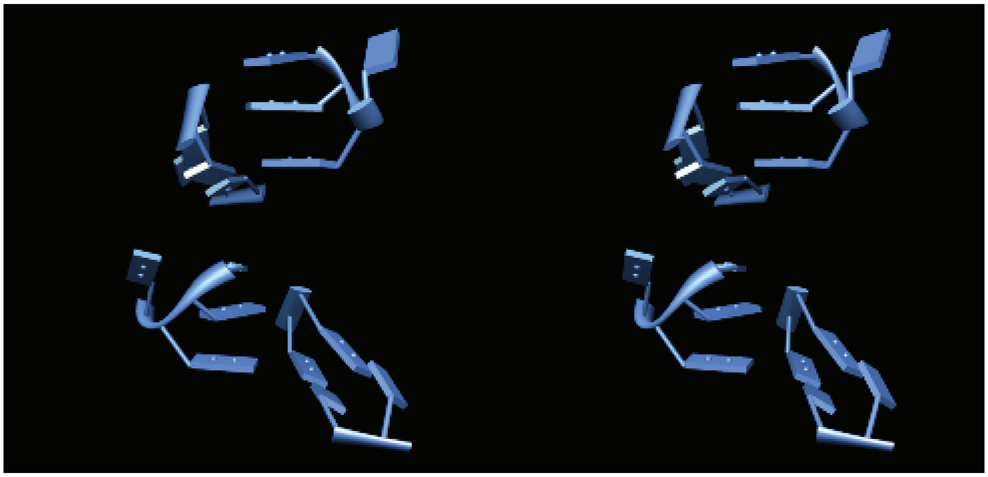

3OFO:159-162

Class II/Subclass 4
(NTL)

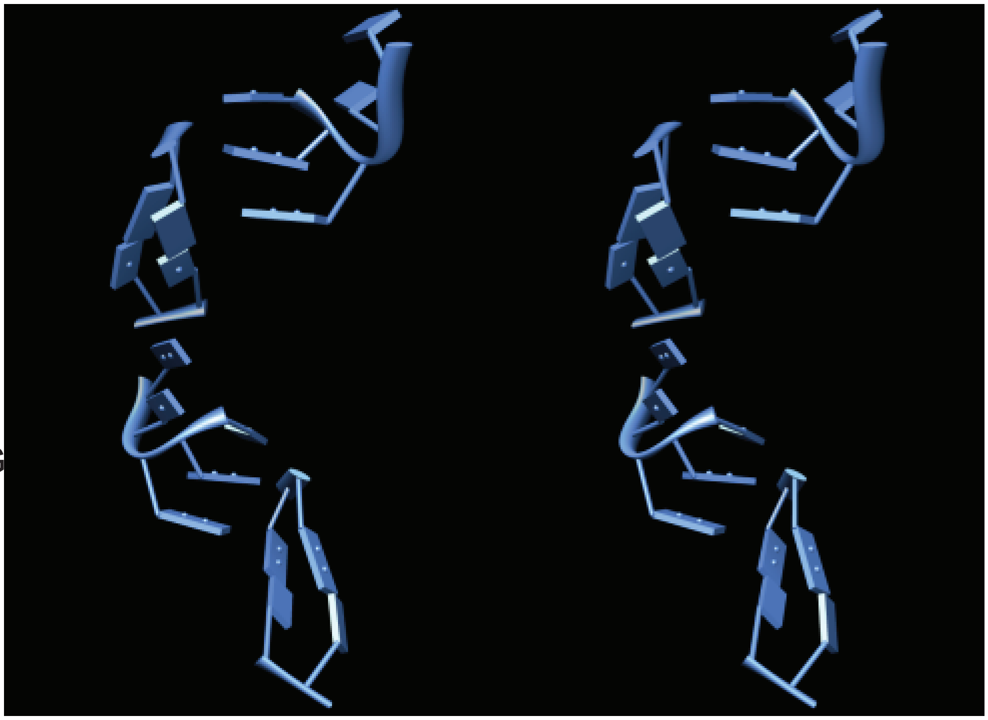

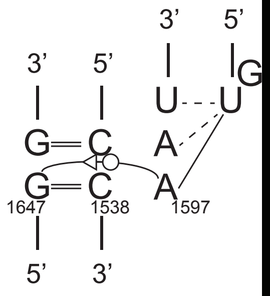

1VQO:1595-1599

B5

Class II/Subclass 5

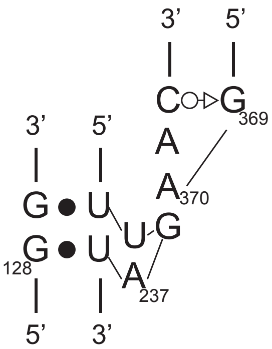

3IGI:369-372

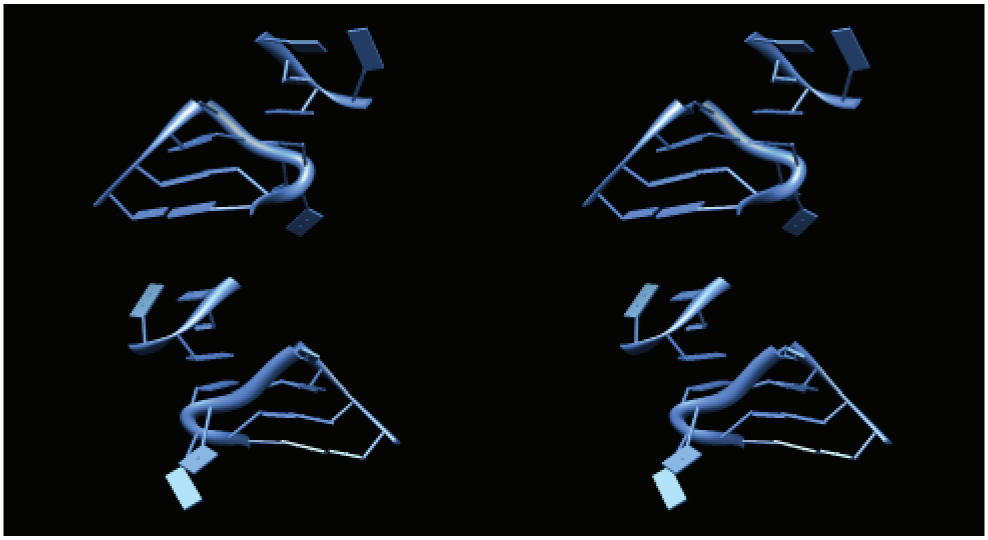

C1

Class III/Subclass 1
(NTL)

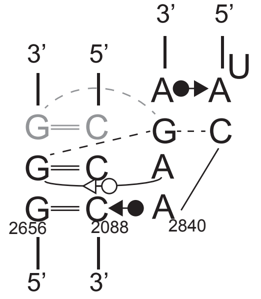

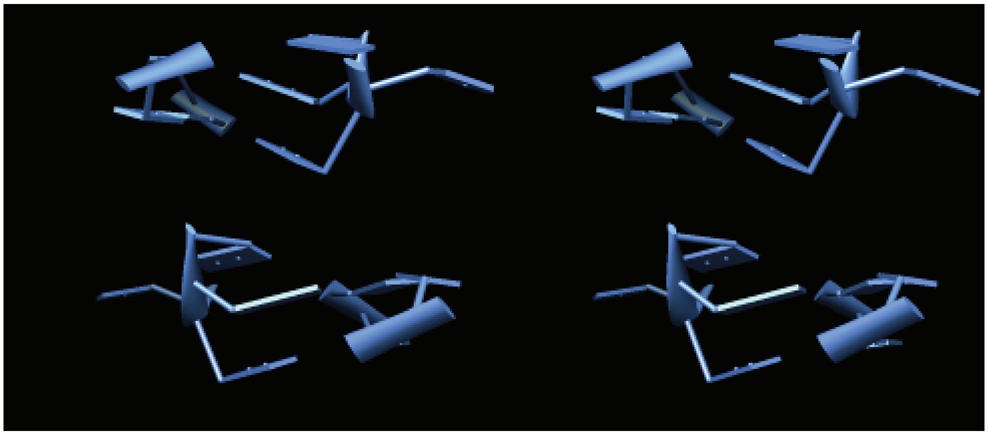

1VQO:2837-2843

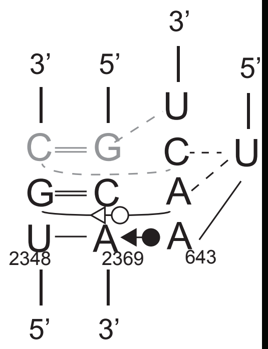

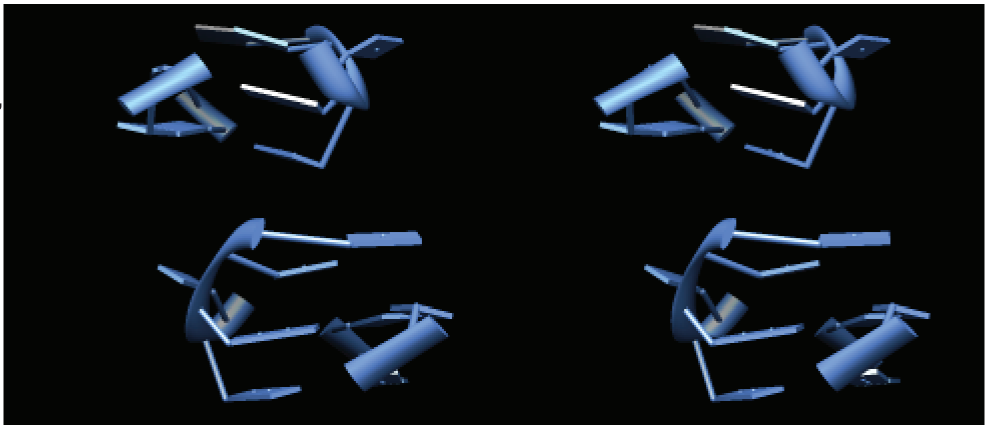

3OFR:642-646

C2

Class III/Individual
(NTL)

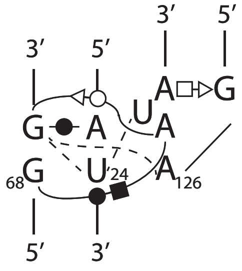

3DIL:125-129

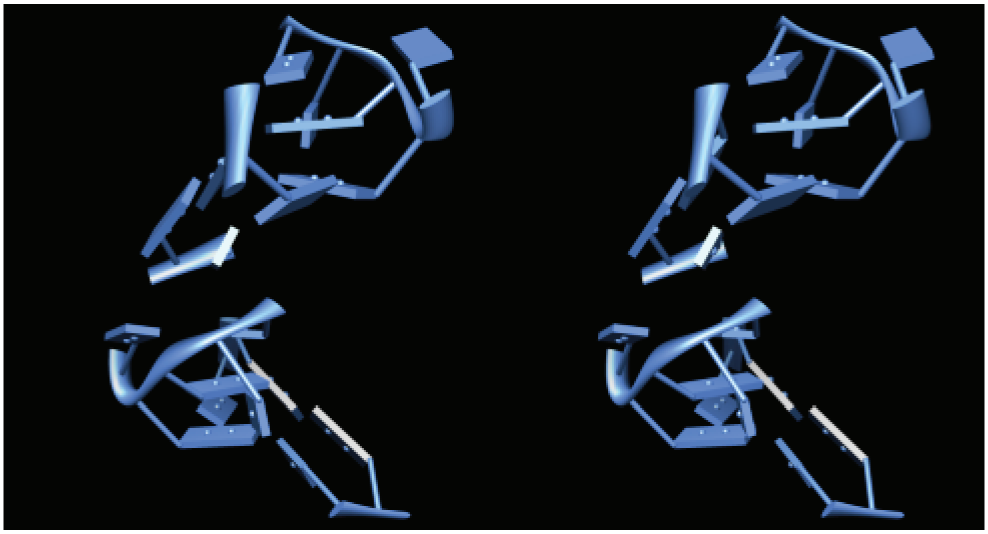

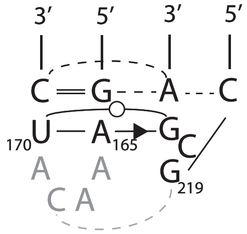

1VQO:218-222

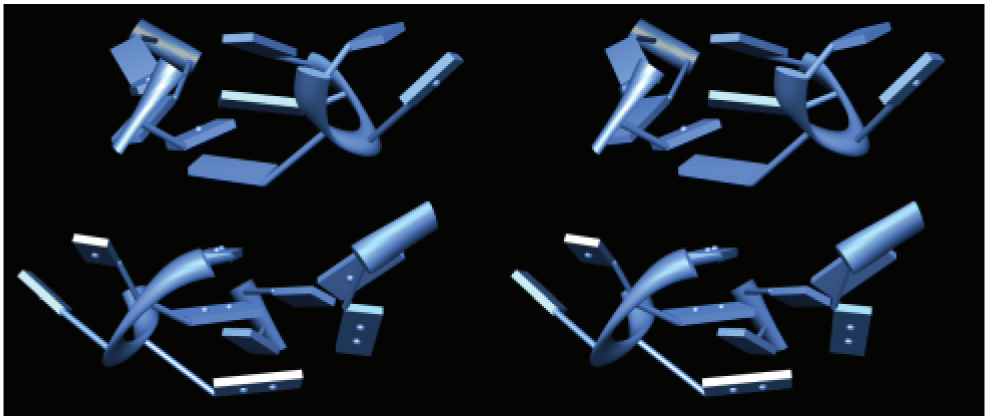

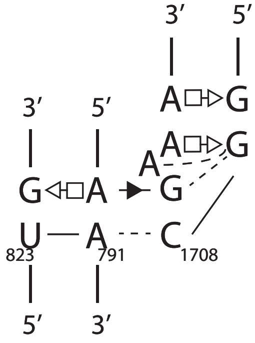

1VQO:1706-1712

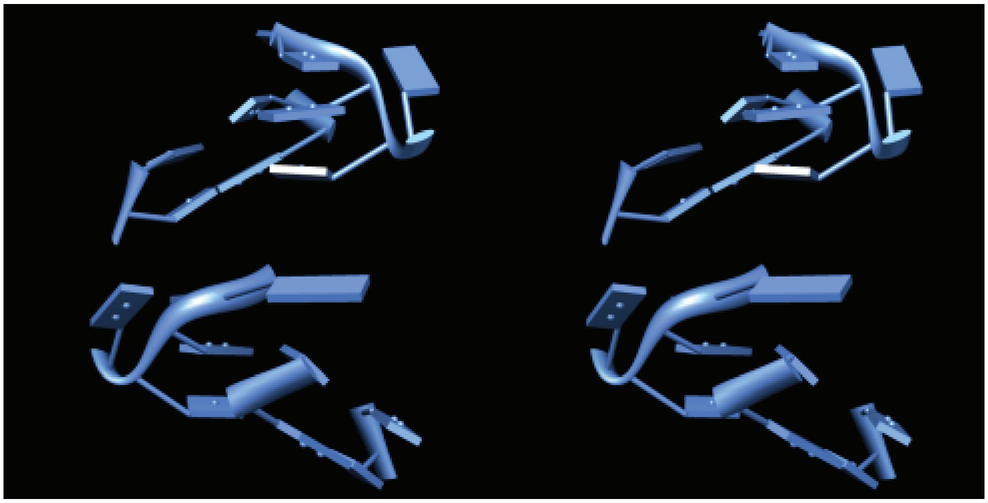

D1

Class IV/Subclass 1
(NTL)

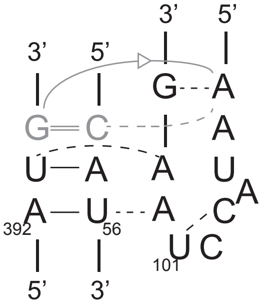

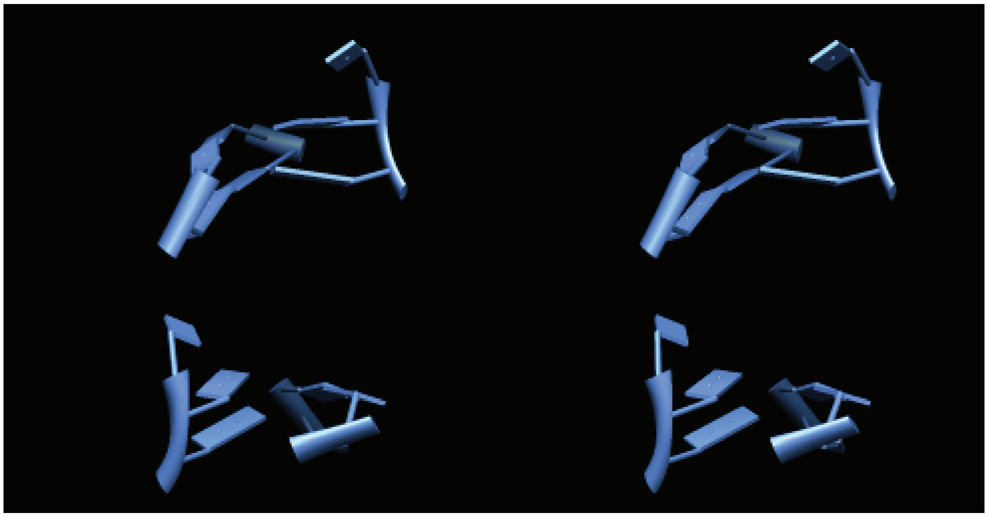

2A64:98-107

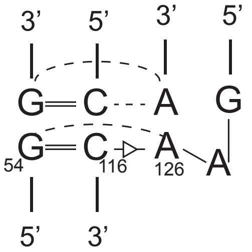

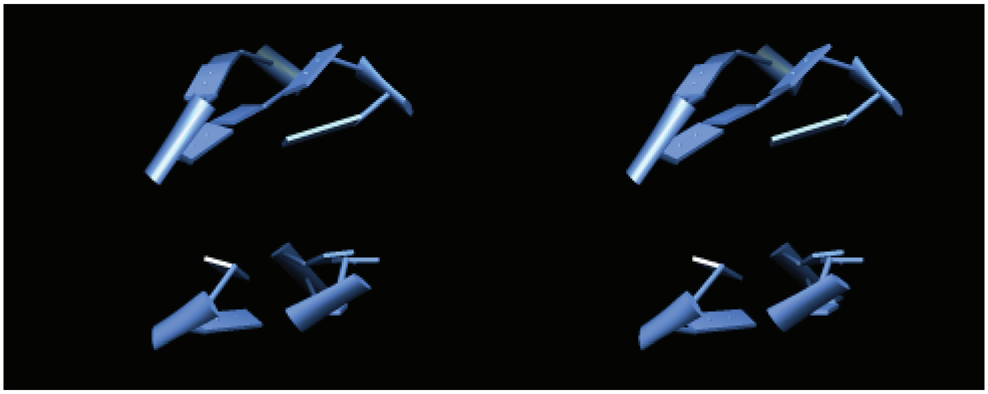

3OFR:124-127

D2

Class IV/Individual (NTL)

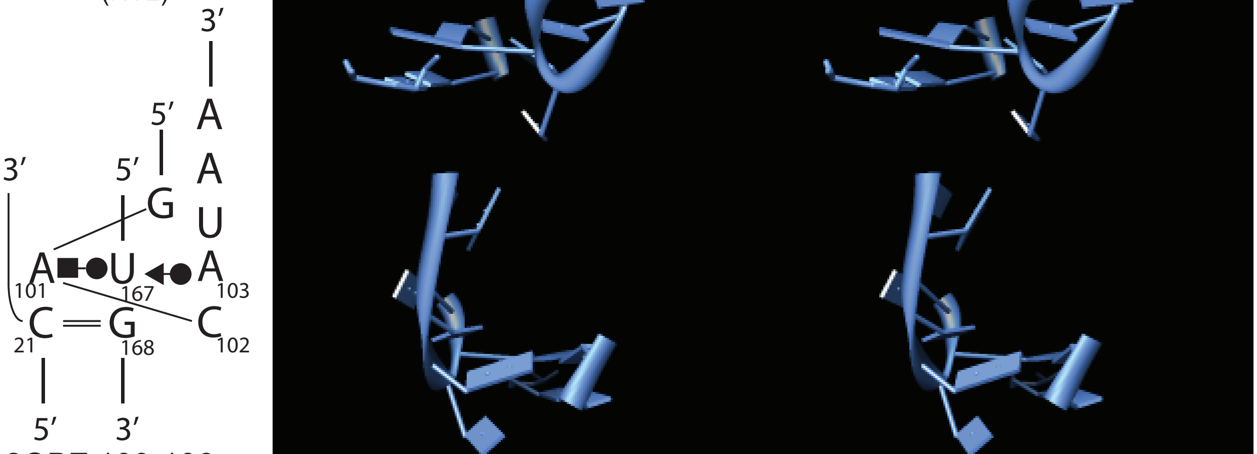

2QBZ:100-106

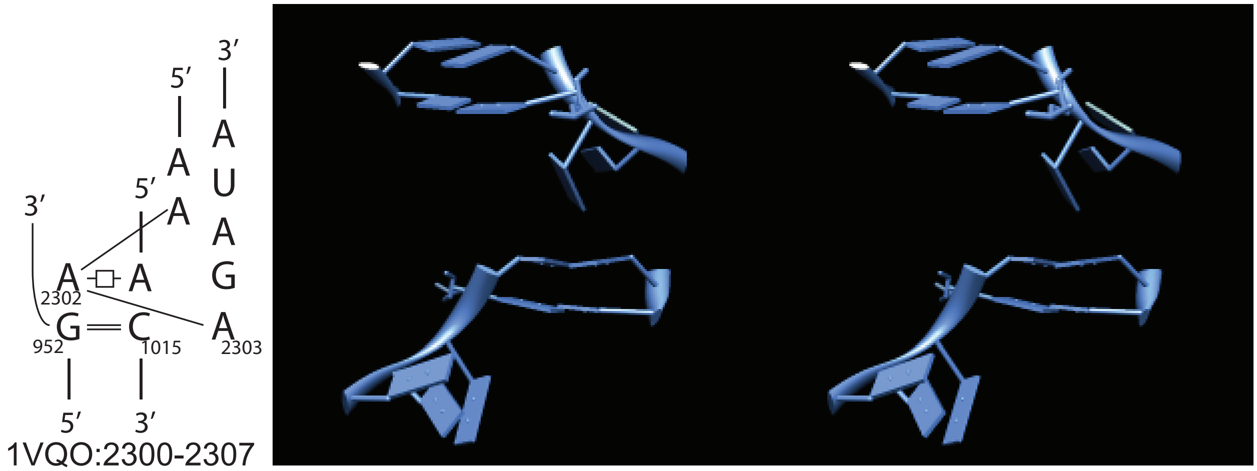

1VQO:2300-2307

D3

Class IV/Individual
(NTL)

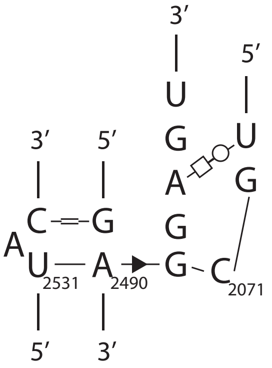

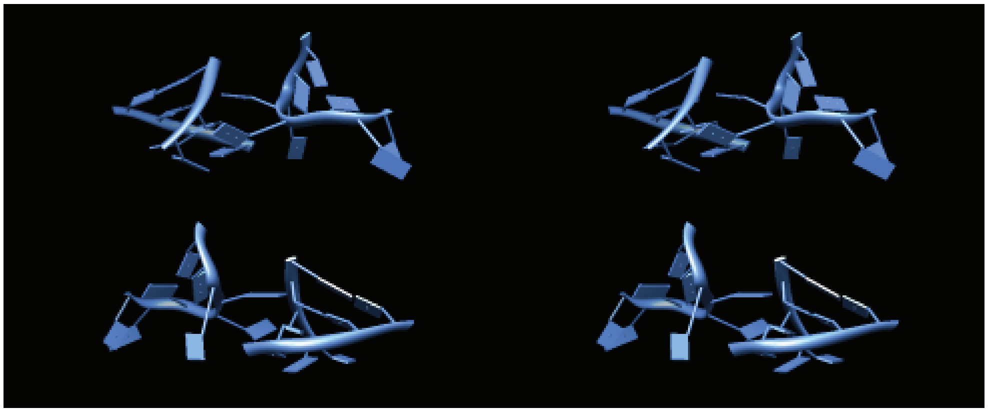

1VQO:2069-2076

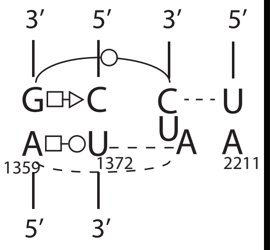

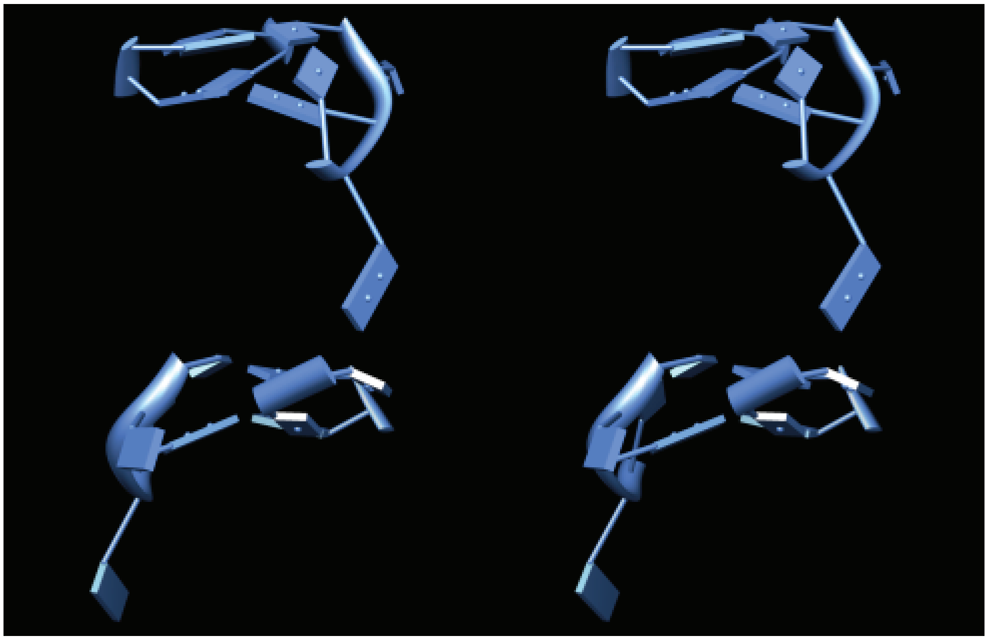

3OFR:2210-2214

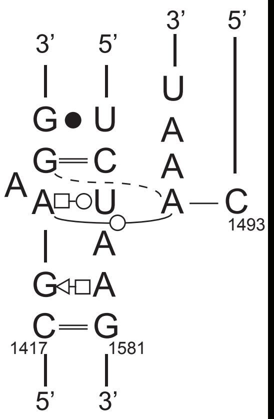

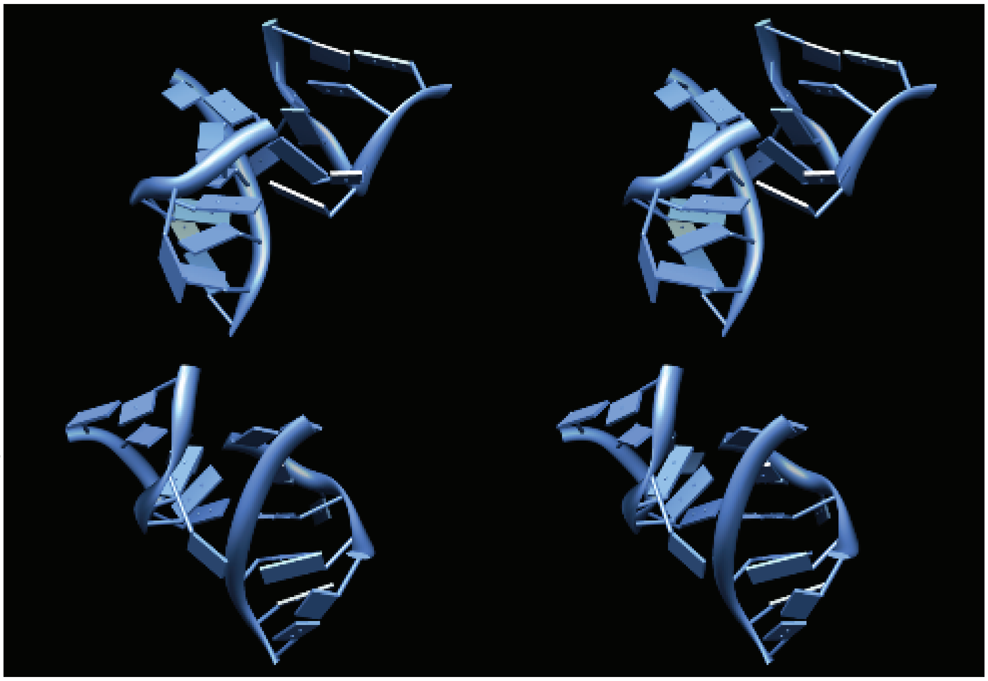

3OFR:1493-1497

D4(#1)

Class IV/Individual
(NTL)

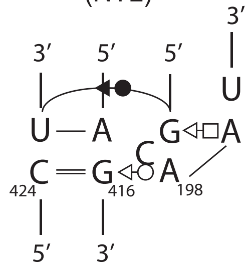

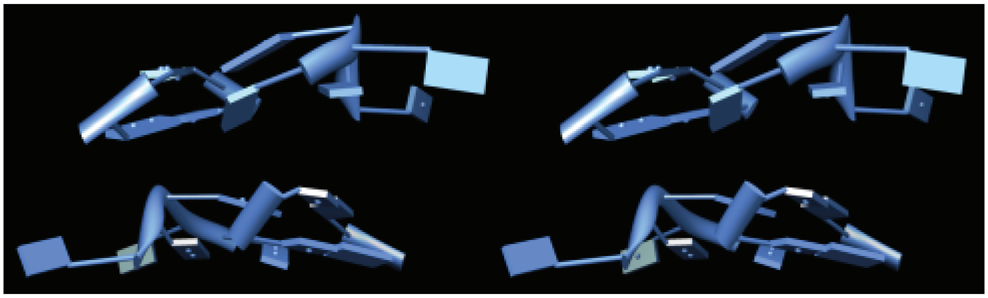

1VQO:196-200

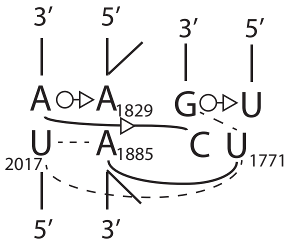

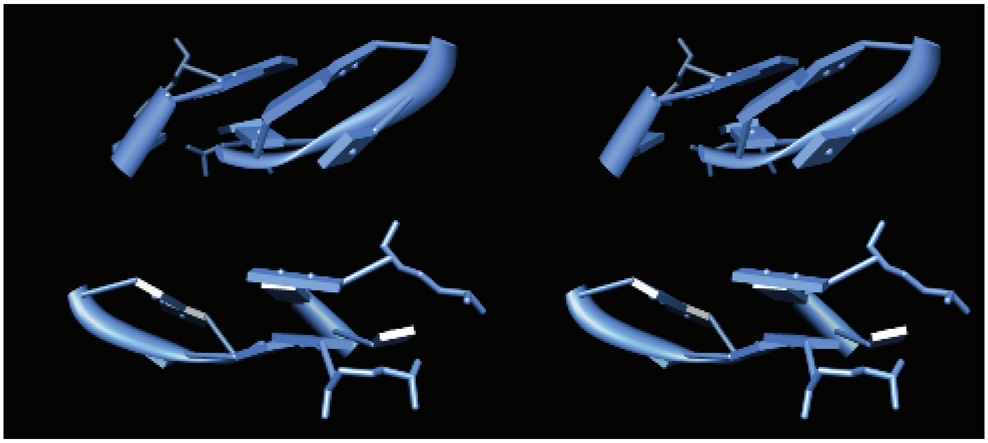

1VQO:1770-1773

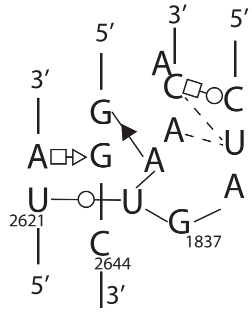

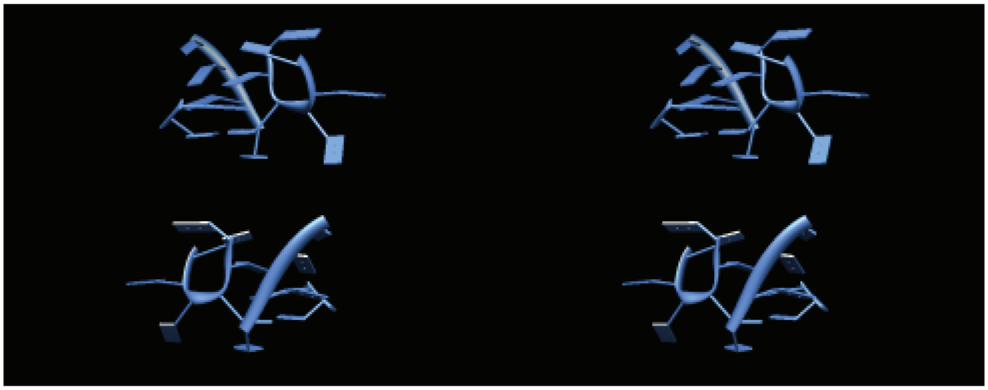

1VQO:1834-1842

D4(#2)

Class IV/Individual
(NTL)

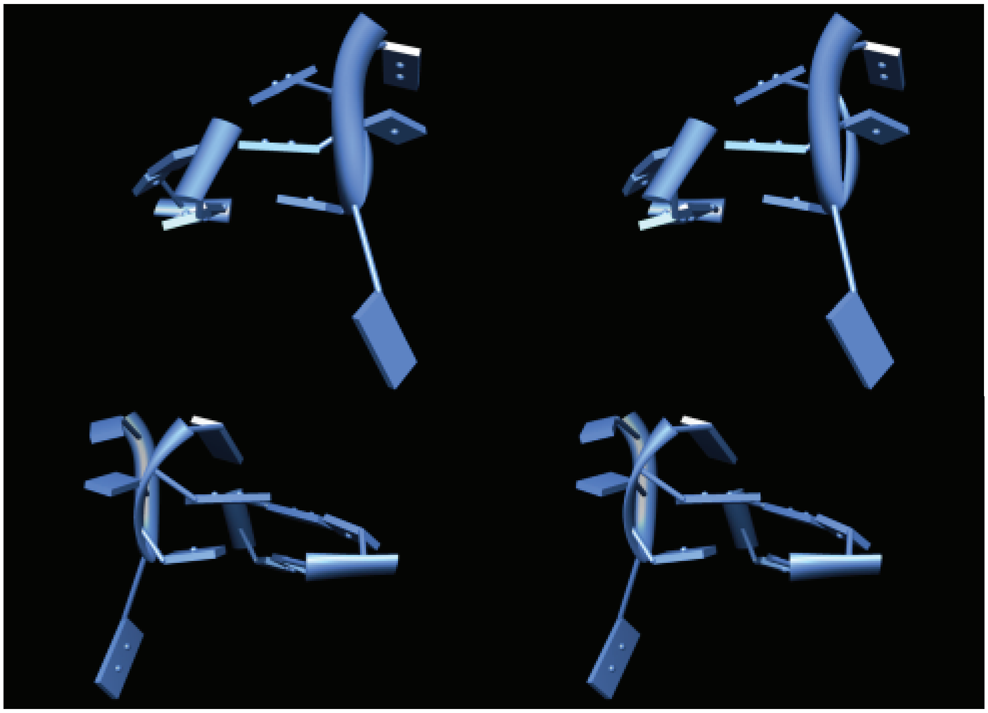

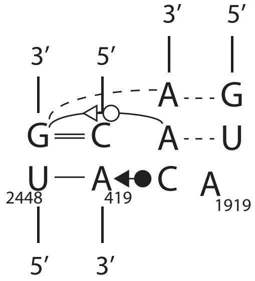

1VQO:1917-1922

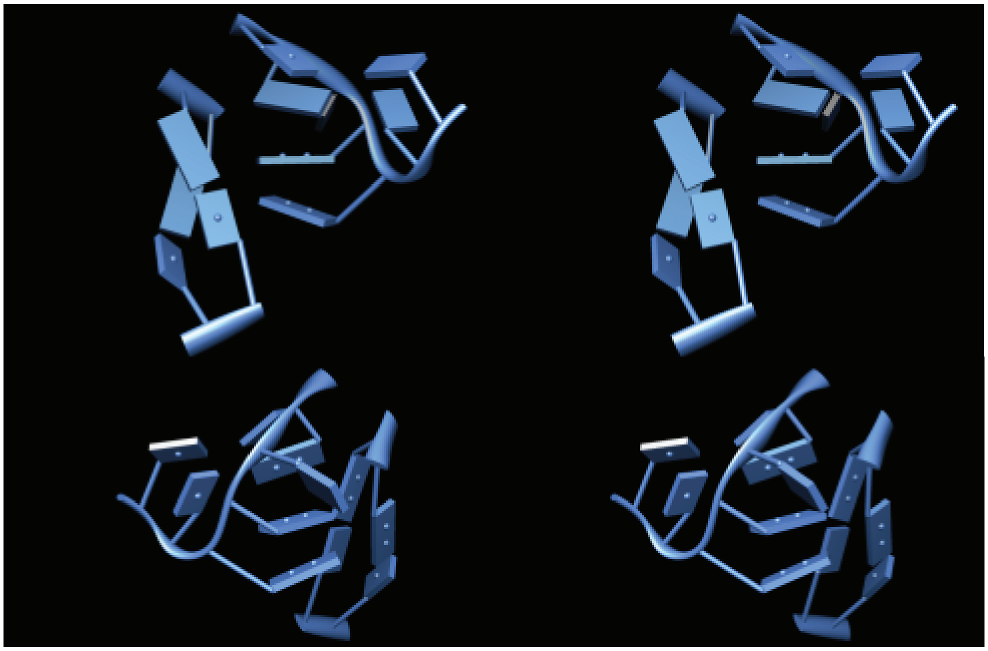

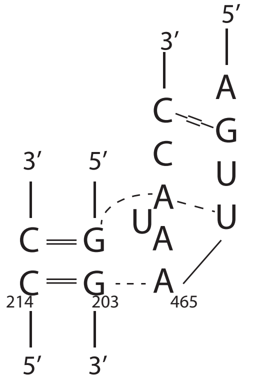

3OFO:461-470

D4(#3)

Class IV/Individual (NTL)

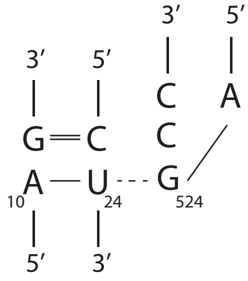

3OFO:523-526

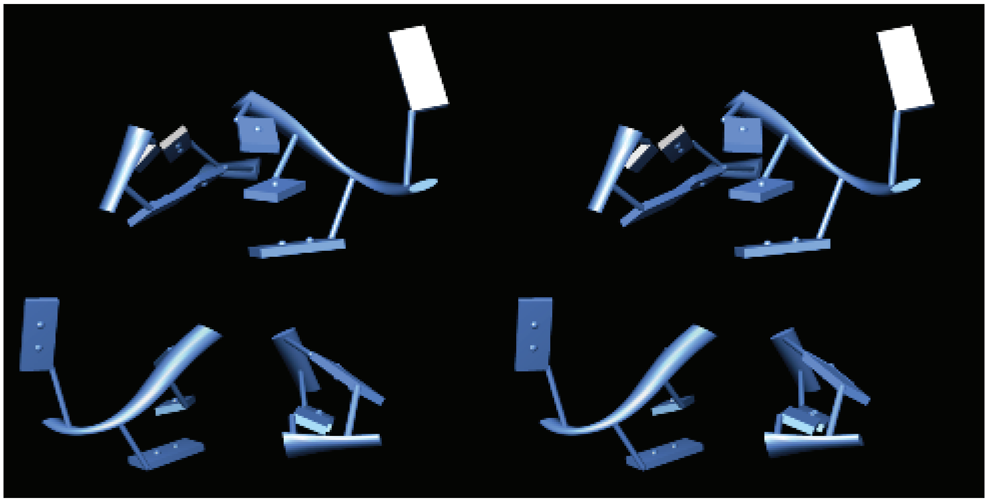

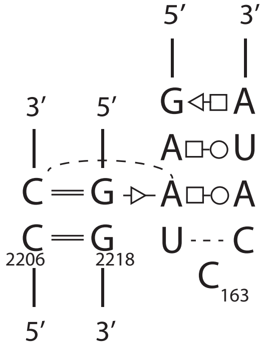

3OFR:159-167

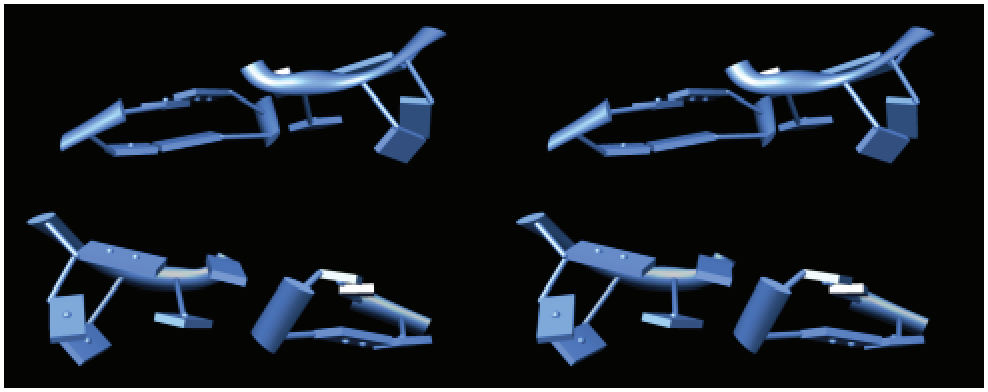

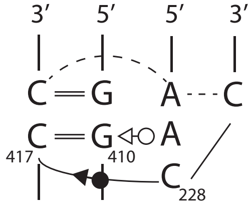

3OFR:226-229

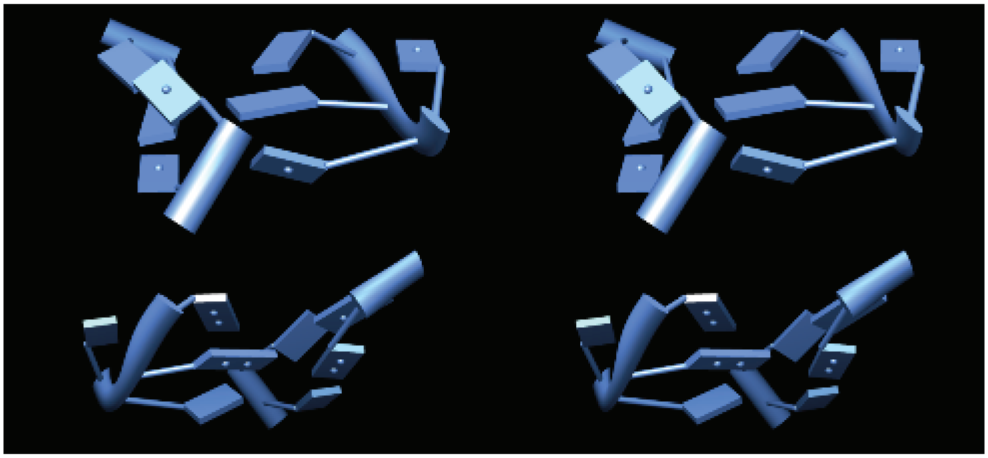

D4(#3)

Class IV/Individual
(NTL)

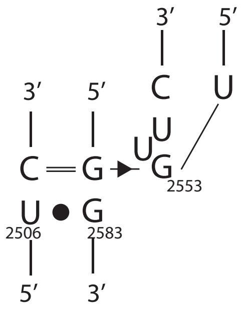

3OFR:2552-2556

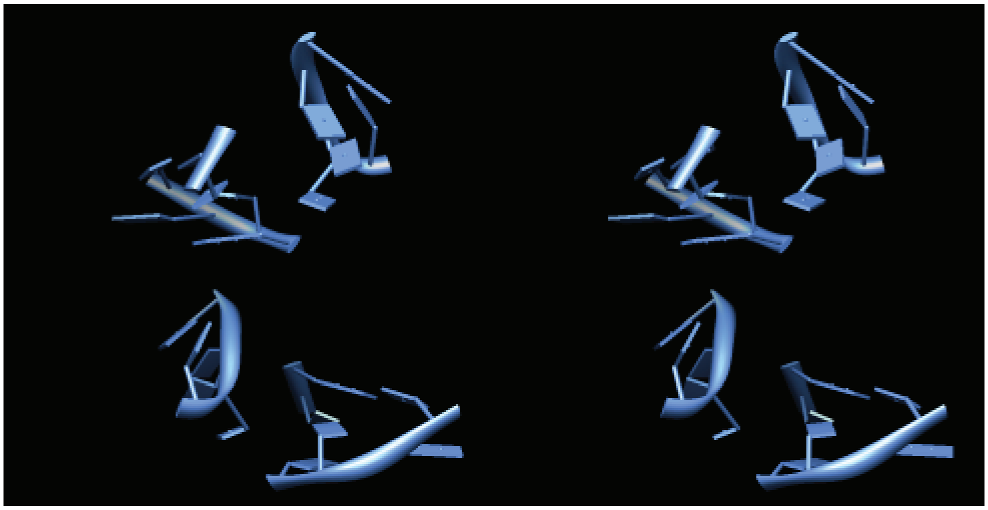

D5(#1)

Class IV/Individual
(NTL)

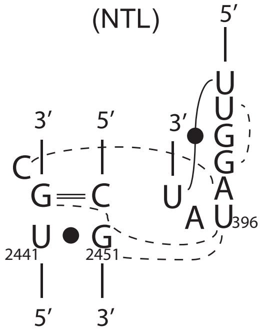

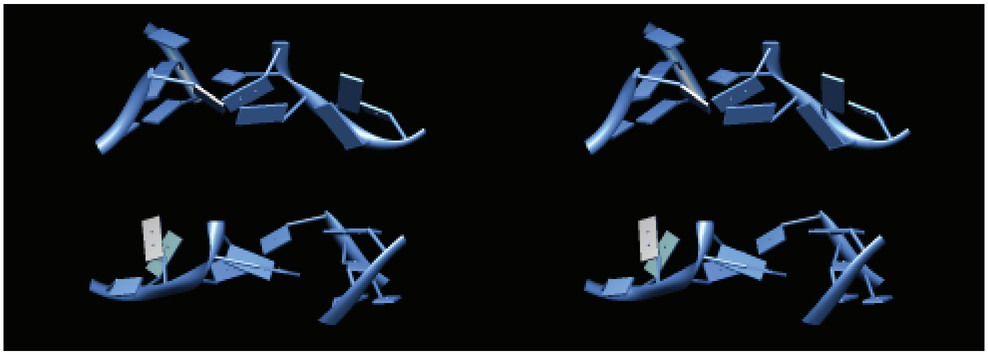

1VQO:391-398

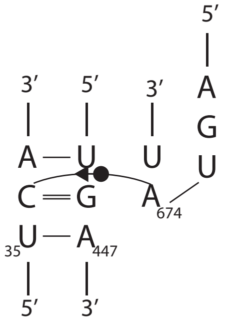

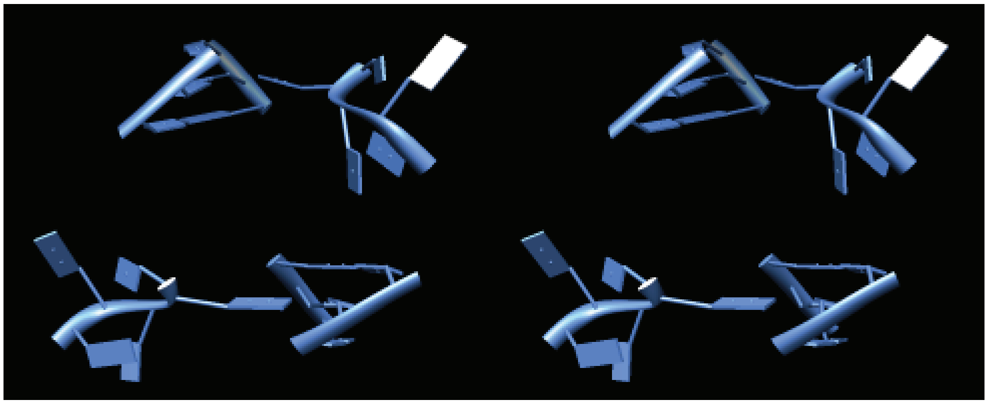

1VQO:671-675

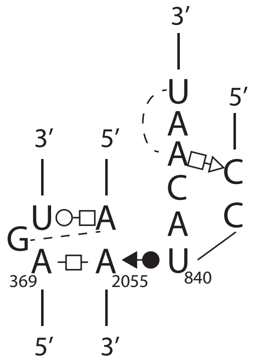

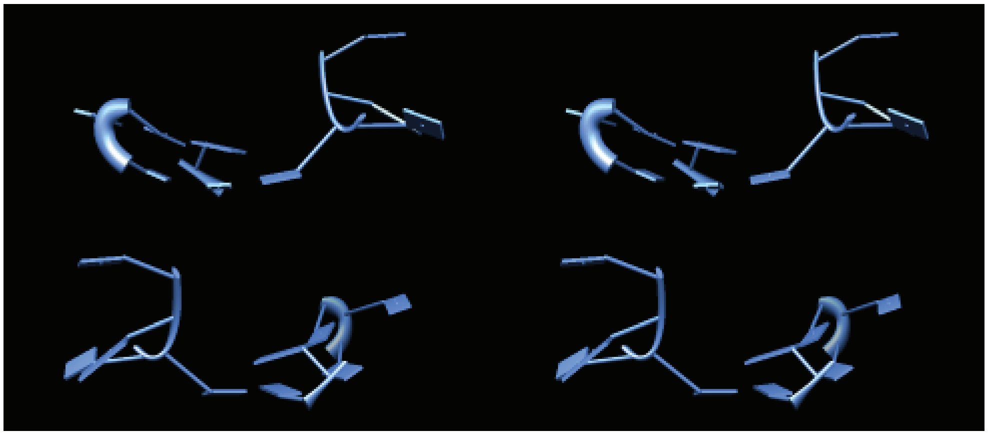

1VQO:838-845

D5(#2)

Class IV/Individual
(NTL)

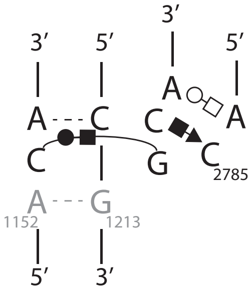

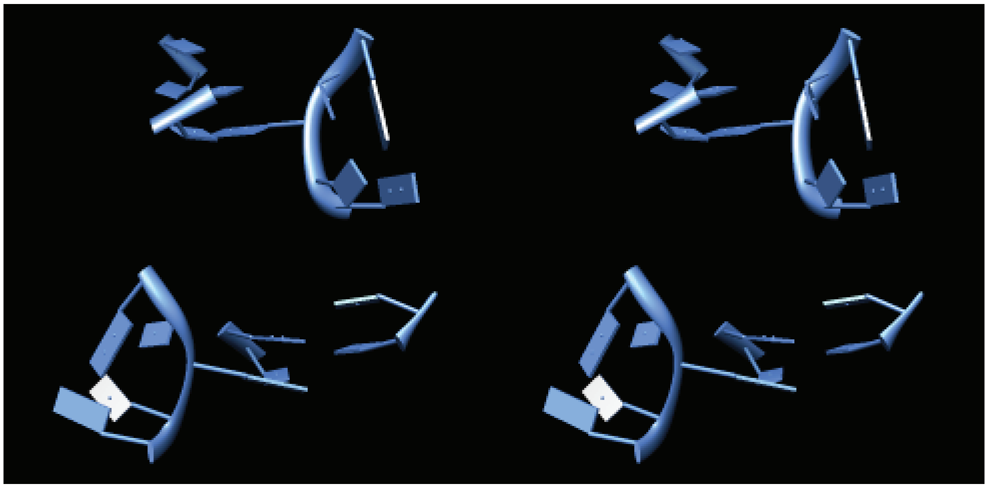

1VQO:2784-2788

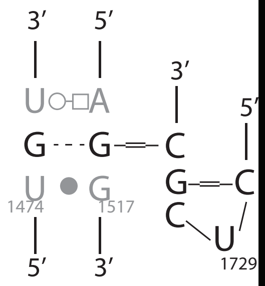

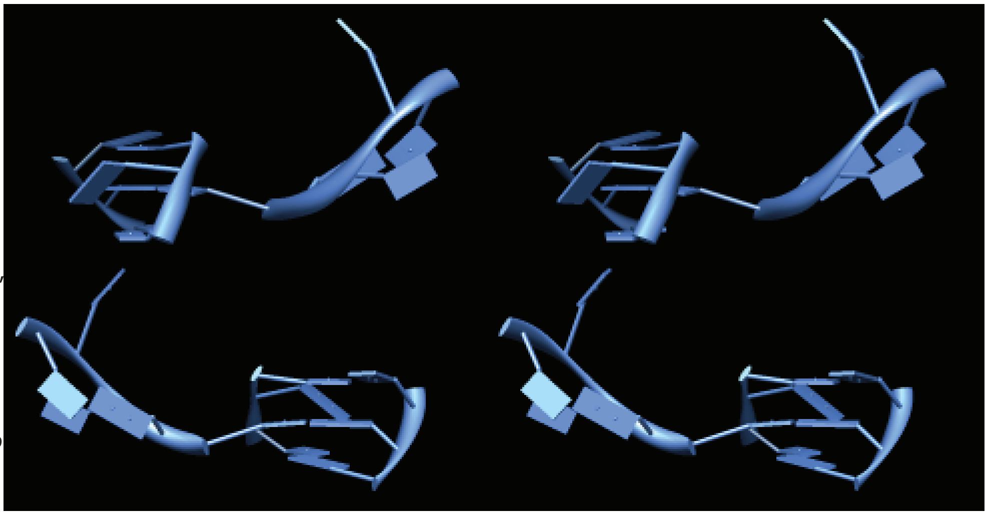

3OFR:1728-1732
