# Supplementary figures and images for: Structural Variation and Uniformity among Tetraloop-Receptor Interactions and Other Loop-Helix Interactions in RNA Crystal Structures
Source: PLoS One. 2012 Nov 9;7(11):e49225. doi: 10.1371/journal.pone.0049225 (PMC3494683; doi:10.1371/journal.pone.0049225)

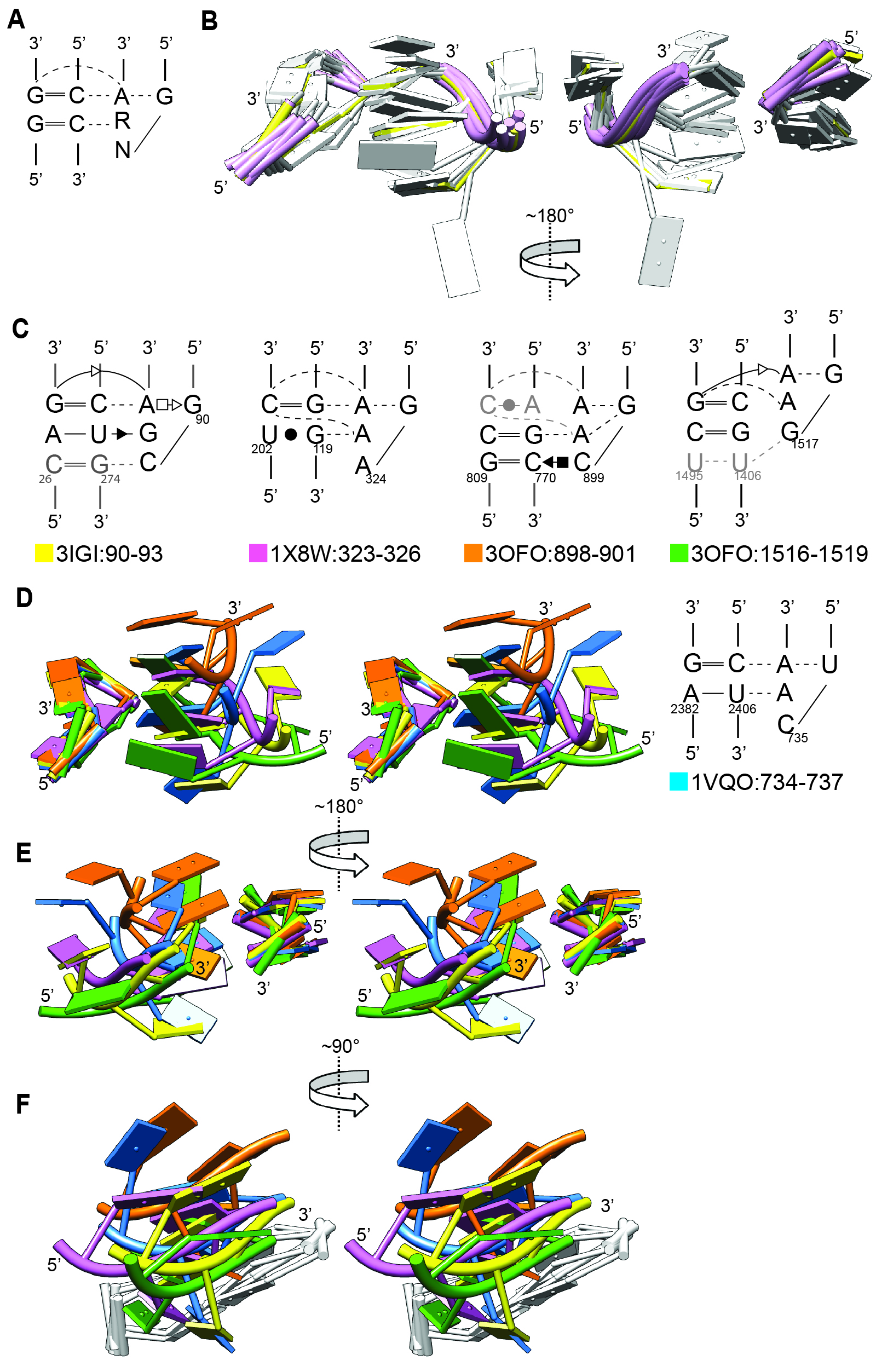

Supplement: Figure S2 — Structures of Class II/Subclasses 1.1 (Indiv) and 1 (Indiv). A) Consensus secondary structure of Class II/Subclass 1.1 (Indiv). B) Two views of an overlay of eight 1.1 (Indiv) structures (purple backbones) and a reference structure of Subclass 1.1.1 (3IGI:90–93) (yellow backbone and bases). C) Secondary structures of the four Subclass 1 (Indiv) structures and the reference 1.1.1 structure (3IGI:90–93) (yellow). Gray bases are not shown in the three-dimensional depictions in panels D, E and F. D,E,F) Superposition of Class II/Subclass 1 (Indiv) structures based on the receptor backbone atoms. Three stereoviews are shown, color-coded as indicated in Panel C. (JPG) [file pone.0049225.s002.jpg]

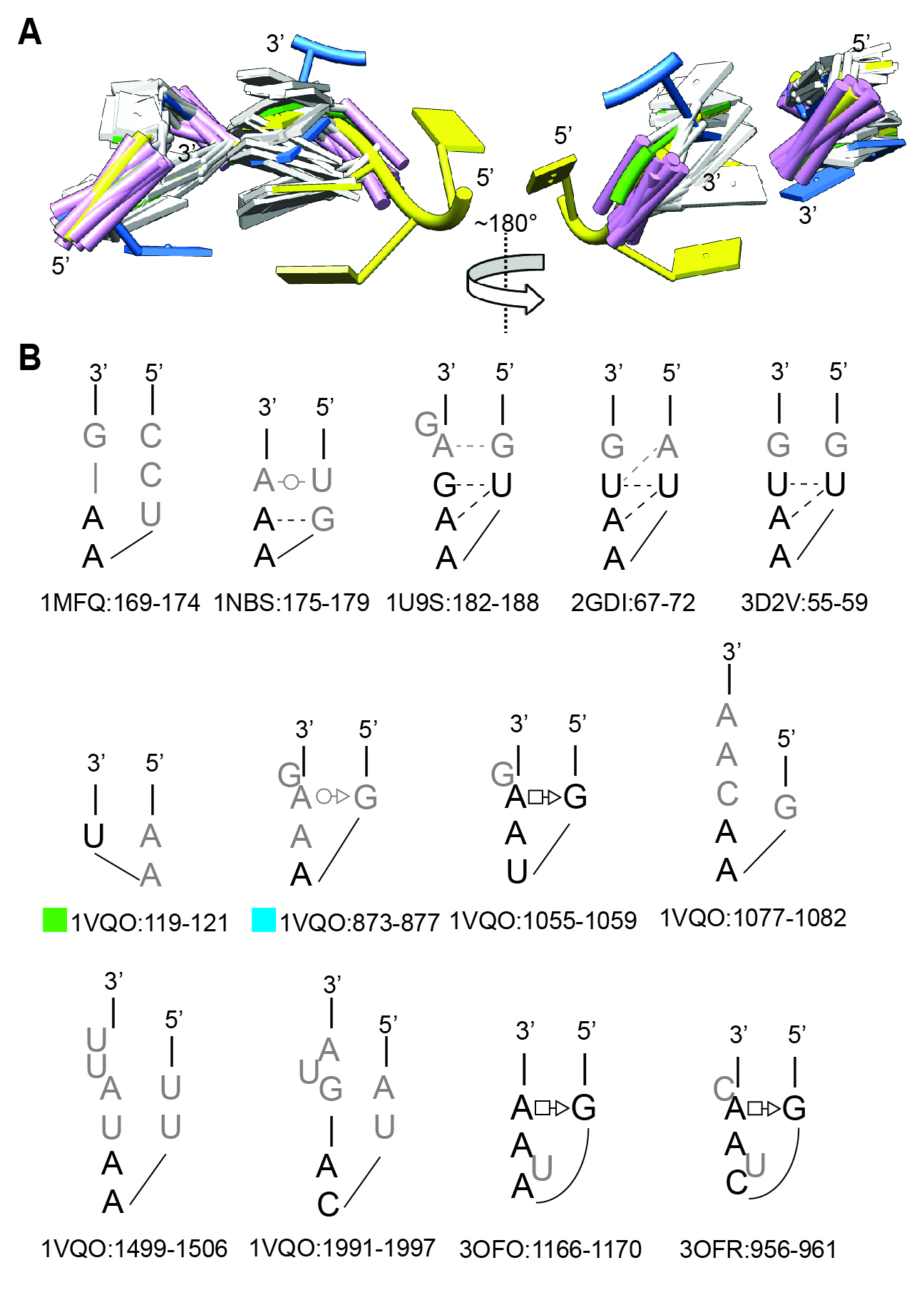

Supplement: Figure S3 — Structures of Class II/Subclass 1 (NTL) structures. A) Overlay of thirteen Subclass I (NTL) structures and a 1.1.1 reference (3IGI:90–93; yellow). Superposition is based on the backbones of 4 nucleotides of the receptor and either one or two nucleotides from the loop (T2, T3 in the case of tetraloops). Green and blue indicate the two loop nucleotides having different backbone geometries but analogous base positions as other structures in the subclass (see Panel B). B) Secondary structures of all Subclass 1 (NTL) loops, with gray indicating nucleotides not adopting the GNRA tetraloop geometry, and not shown in Panel A. (JPG) [file pone.0049225.s003.jpg]

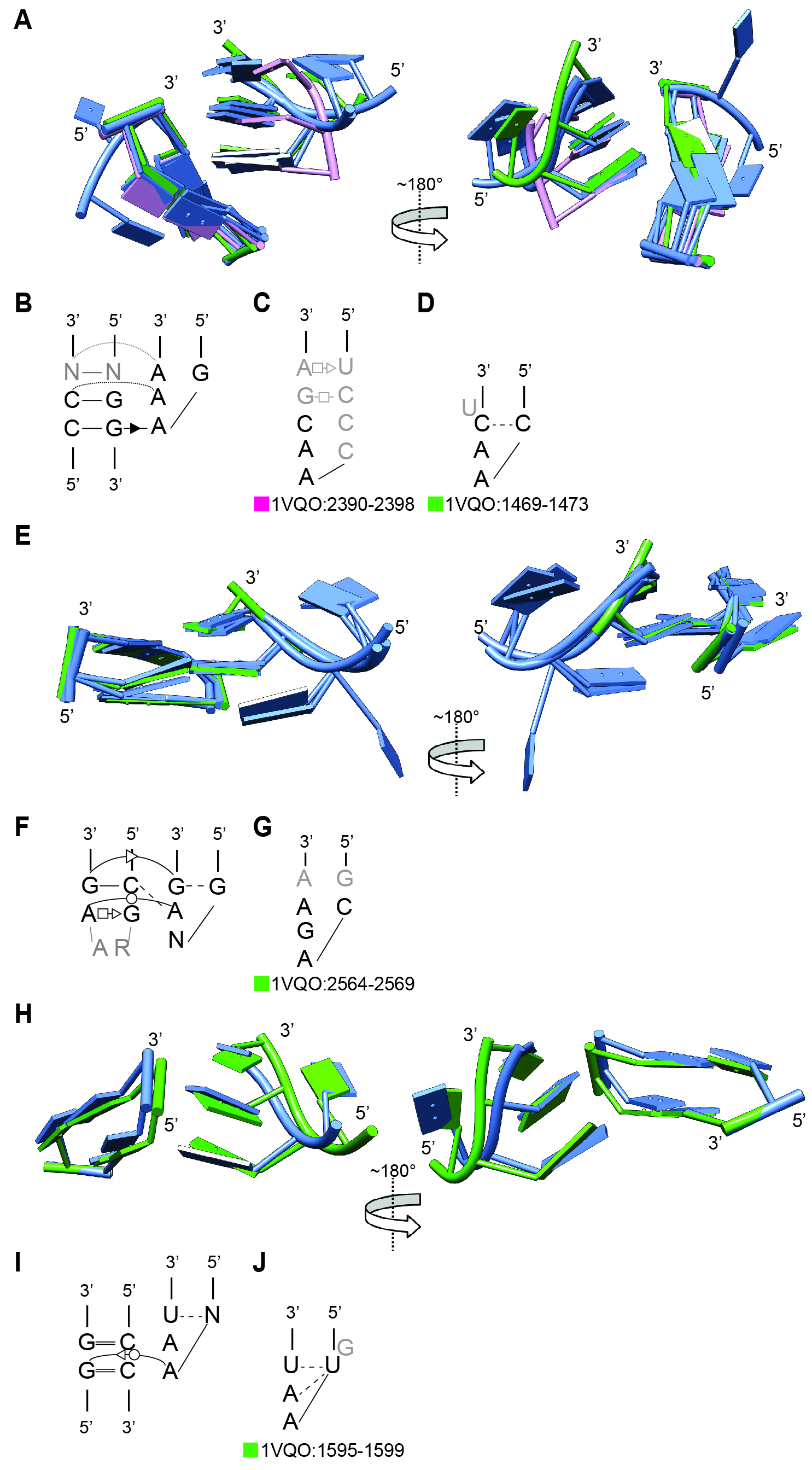

Supplement: Figure S4 — Secondary and tertiary structures of Class II Subclasses 2, 3 and 4. A) Two views of the overlay of five structures of Class II/Subclass 2. Blue nucleotides represent the three tetraloop-receptor structures, while pink and green nucleotides represent the NTL-receptor structures (see panels B, C, D). B) Consensus secondary structure for the five structures in Subclass 2. C, D) Secondary structures for Subclass 2 (NTL) loops. E) Two views of the overlay of four structures of Class II/Subclass 3. Blue nucleotides represent the three tetraloop-receptor structures, while green nucleotides represent the NTL-receptor structure. F) Consensus secondary structure for the four structures in Subclass 3. G) Secondary structure for the NTL loop in Subclass 3. H) Two views of an overlay of Class II/Subclass 4 structures. Blue nucleotides represent the tetraloop-receptor structure, while green nucleotides represent the NTL interaction. I) Consensus secondary structure for Subclass 4. J) Secondary structure for the NTL loop of Class II/Subclass 4. (JPG) [file pone.0049225.s004.jpg]

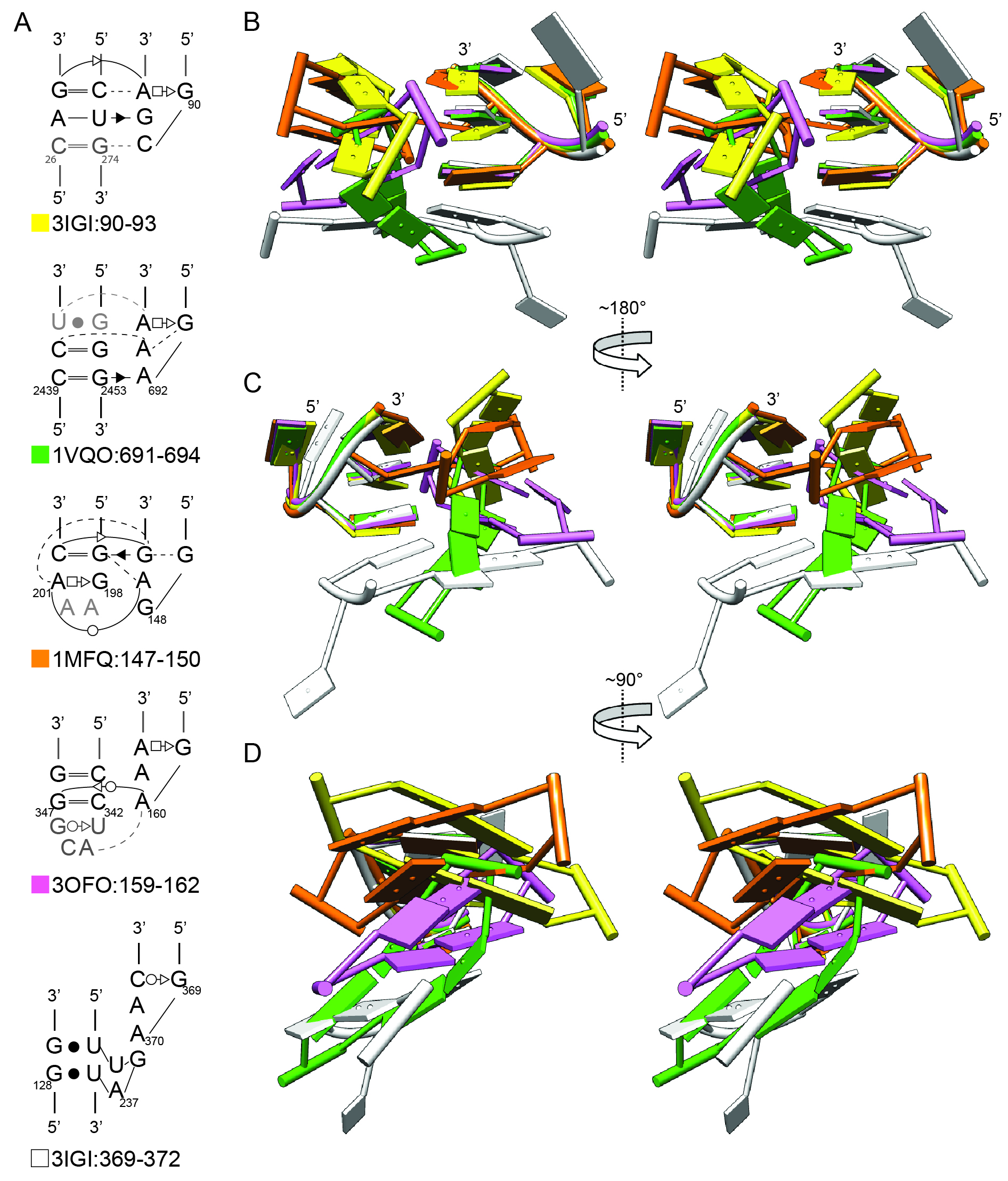

Supplement: Figure S5 — Superposition of representative Class II structures from all five classes. A) Secondary structures of representatives from each class (Subclass 1, 3IGI:90–93 (yellow); Subclass 2, 1VQO:691–694 (green); Subclass 3, 1MFQ:147–150 (orange); Subclass 4∶3OFO:159–162 (pink); Subclass 5∶3IGI:369–372 (white)). B, C, D) Three stereoviews of the superposition, based on the backbone atoms of the tetraloop, and color-coded as in panel A. (JPG) [file pone.0049225.s005.jpg]

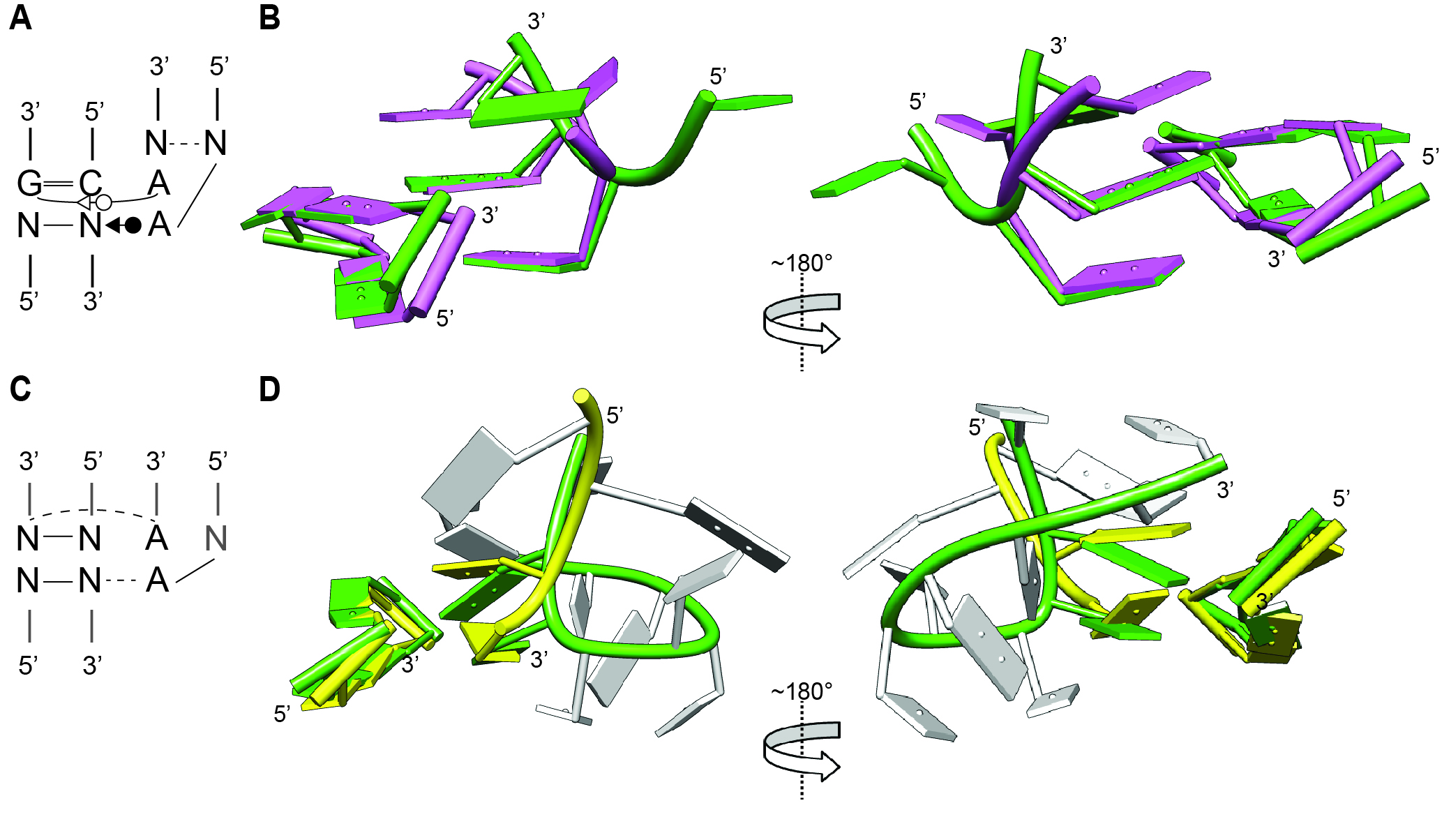

Supplement: Figure S6 — Secondary and tertiary structures of Classes III and IV. A) Consensus secondary structure for Class III/Subclass 1. B) Two views of the superposition of VQO:2837–2843 (green) and 3OFR:642–646 (purple). C) Consensus secondary structure for Class IV/Subclass 1. D) Two views of the superposition of 2A64∶98–107 (green) and 3OFR:124–127 (yellow). Gray nucleotides are loop nucleotides that do not interact with the receptor. (JPG) [file pone.0049225.s006.jpg]

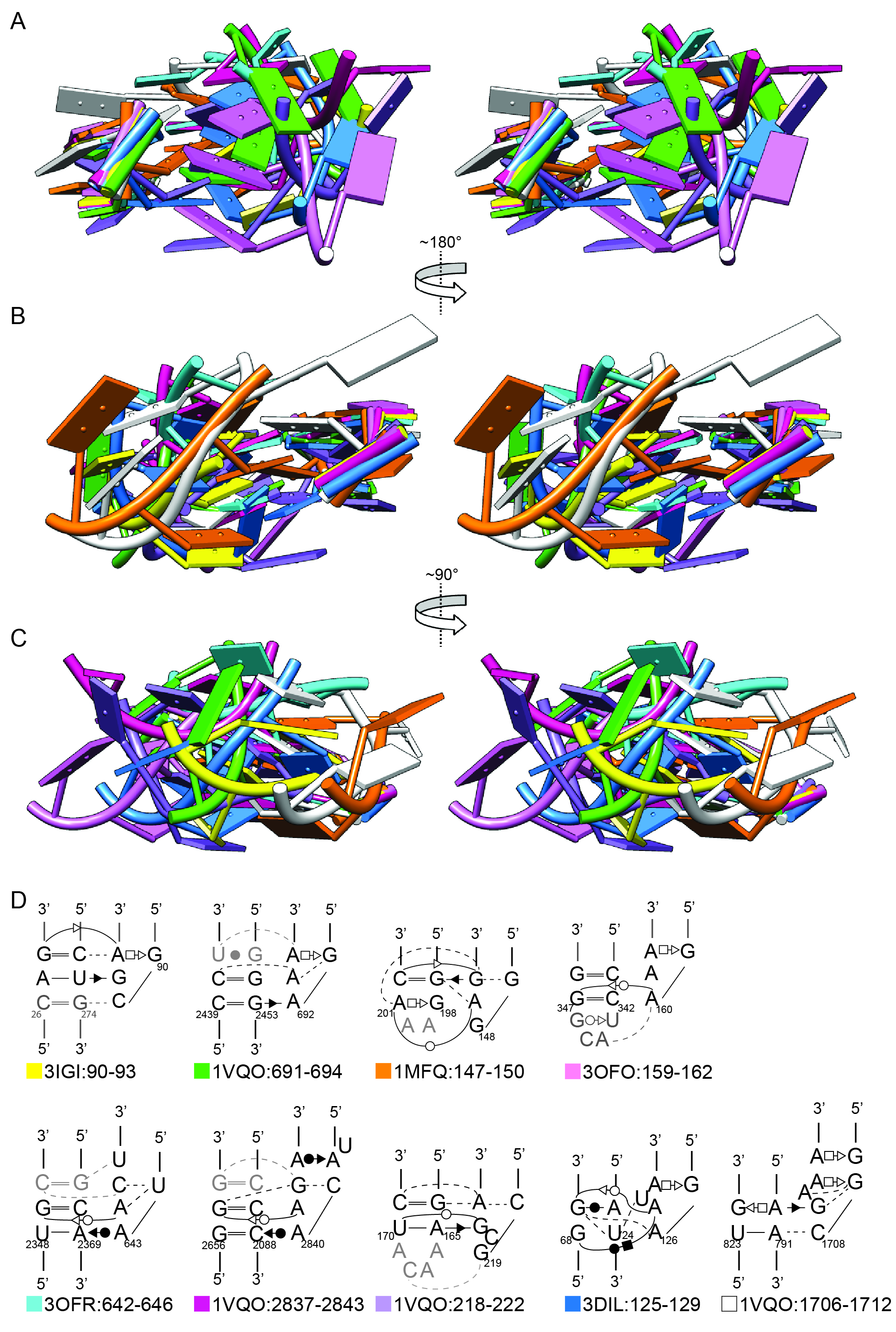

Supplement: Figure S7 — Comparison of structures in Classes II and III. A, B, C) Three stereoviews of the overlay of four representatives from Class II/Subclasses I–IV and all five structures from Class III. Superposition is based on the backbone atoms of the four receptor nucleotides. D) Secondary structure depictions and color-coding for the three-dimensional depictions above. (JPG) [file pone.0049225.s007.jpg]

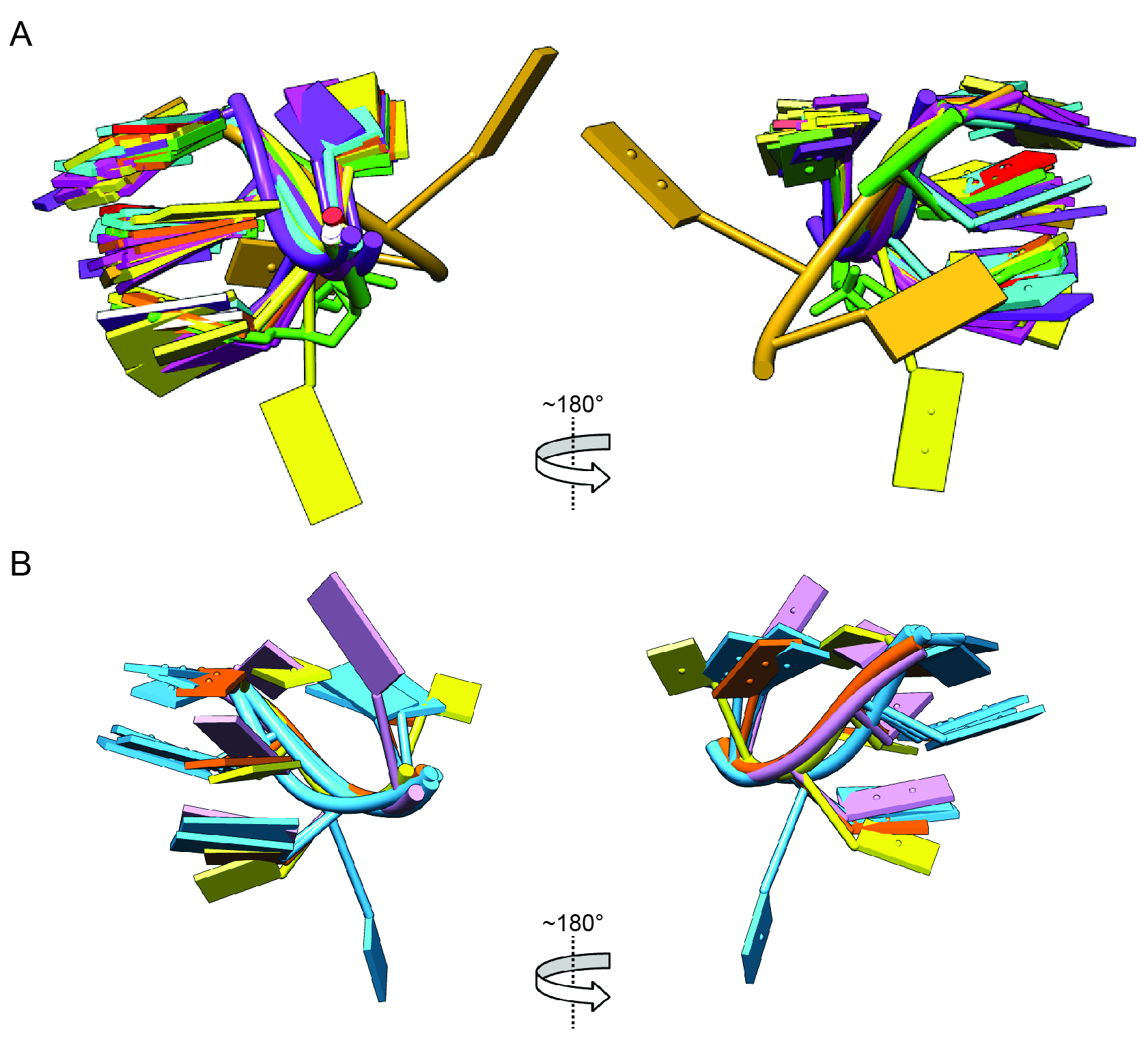

Supplement: Figure S8 — Superposition of GNRA loop structures. A. Superposition of 30 GNRA tetraloop structures. Green (Class I) 1NBS:205–208, 1U6B:24–27, 1U6B:189–192, 2R8S:150–153; Pink (Class II Subclass 1.1.1) 2Z75∶114–117, 3IGI:90–93, 3OK7∶93–96, 3OK7∶285–288, 3OFO:1077–1080, 3OFO:1266–1269, 3OFR:2857–2860; Orange, Class II Subclass 1.1.2, 1U9S:205–208, 3MXH:32–35, 1VQO:1629–1632, 1VQO:1863–1866; Gold, Class II Subclass 1.1 (Indiv) 1Y0Q:22–25, 1Y0Q:205–208, 1VQO:469–4723, 1VQO:577–580, 1VQO:1327–13303, 1VQO:2630–26333, 3OFO:1013–1016, 3OFR:1807–1810; Purple (Class II Subclass 1 (Indiv)) 1X8W:323–326, 3OFO:898–901, 3OFO:1516–1519; Light blue (Class II Subclass 2) 1VQO:691–694, 3OFR:630–633, 3OFR:1364–1367; Red (Class II Subclass 4) 3OFO:159–162; Burnt orange (Class IV Subclass 1) 3OFR:124–127. B. Superposition of five non-GNRA sequences that assume GNRA tetraloop structures. Gold (Class II Subclass I (Indiv)) (UCAA sequence) 1VQO:734–737; Blue (Class II Subclass 3) (GNAG sequence) 1LNG:163–166, 1MFQ:147–150, 3KTW:164–167; Pink (Class II Subclass 5) (GAAC sequence) 3IGI: 369–372. (JPG) [file pone.0049225.s008.jpg]
