# Supplementary material for: Structural Variation and Uniformity among Tetraloop-Receptor Interactions and Other Loop-Helix Interactions in RNA Crystal Structures
Source: PLoS One. 2012 Nov 9;7(11):e49225. doi: 10.1371/journal.pone.0049225 (PMC3494683; doi:10.1371/journal.pone.0049225)
Supplement: Table S1 — Compiled list of nonredundant crystal structures for RNAs greater than 50 nucleotides and with resolution greater than 4 Å (as of Dec. 31, 2010). (DOC) [file pone.0049225.s010.doc]

**Table S1. Compiled list of nonredundant crystal structures for RNAs greater than 50 nucleotides and with resolution greater than 4 Å (as of Dec. 31, 2010).**

| PDB ID | Macromolecule Name | Source | Length (nts) | Resolution (Å) |
| --- | --- | --- | --- | --- |
| RNase P | | | | |
| 3OK7^1^ | RNase P holoenzyme with tRNA (type A) | *Thermotoga maritima* | 347 | 3.8 |
| 1U9S^2^ | RNase P RNA specificity domain (type A) | *Thermus thermophilus* | 161 | 2.9 |
| 2A64^3^ | RNase P RNA (type B) | *Bacillus stearothermophilus* | 417 | 3.3 |
| 1NBS^4^ | RNase P RNA specificity domain (type B) | *Bacillus subtilis* | 155 | 3.15 |
|  |  |  |  |  |
| Group I introns | | | | |
| 2R8S^5^ | P4-P6 ribozyme domain | *Tetrahymena thermophila* | 159 | 1.95 |
| 1X8W^6^ | Group I ribozyme | *Tetrahymena thermophila* | 247 | 3.8 |
| 1U6B^7^ | Group I ribozyme with both exons | *Azoarcus sp.* | 197 | 3.1 |
| 1Y0Q^8^ | Group I ribozyme | *Staphylococcus phage Twort* | 229 | 3.6 |
|  |  |  |  |  |
| Group II introns | | | | |
| 1KXK^9^ | Group II intron domains 5,6 (ai5g) | *Saccharomyces cerevisiae* | 70 | 3.0 |
| 3IGI^10^ | Group IIC intron | *Oceanobacillus iheyensis* | 412 | 3.12 |
|  |  |  |  |  |
| Small ribozymes | | | | |
| 3NKB^11^ | Hepatitis delta virus ribozyme | Hepatitis delta virus | 64 | 1.92 |
| 1M5O^12^ | Hairpin ribozyme | Tobacco ringspot virus | 92 | 2.2 |
| 2QUW^13^ | Hammerhead ribozyme, cleaved fragment | Tobacco ringspot virus | 57 | 2.2 |
| 3IVK^14^ | RNA polymerase ribozyme | Synthetic | 128 | 3.1 |
| 3CUL^15^ | Aminoacyl-tRNA synthetase ribozyme | Synthetic | 92 | 2.8 |
| 2Z75^16^ | GlmS ribozyme RNA | *Thermoanaerobacter tengcongensis* | 125 | 1.7 |
| 3L3C^17^ | GlmS ribozyme RNA | *Bacillus anthracis* | 141 | 2.85 |
| 2OIU^18^ | L1 Ribozyme, ligase circular adduct | Synthetic | 71 | 2.6 |
|  |  |  |  |  |
| Riboswitches | | | | |
| 3GX5^19^ | SAM-I riboswitch variant bound to SAM | *Thermoanaerobacter tengcongensis* | 94 | 2.4 |
| 2QWY^20^ | SAM-II riboswitch bound to SAM | Environmental sequence | 52 | 2.8 |
| 3E5C^21^ | SMK box (SAM-III) riboswitch with SAM | *Enterococcus faecalis* | 53 | 2.25 |
| 3NPB^22^ | TL5 RNA (SAM-I), larger molecule | *Bacillus subtilis* | 119 | 3.02 |
| 2QBZ^23^ | M-Box riboswitch aptamer domain | *Bacillus subtilis* | 161 | 2.6 |
| 3LA5^24^ | Adenosine riboswitch | *Vibrio vulnificus* | 71 | 1.7 |
| 3NPQ^25^ | S-adenosylhomocysteine riboswitch | *Ralstonia solanacearum* | 54 | 2.18 |
| 2G9C^26^ | Guanine riboswitch | *Bacillus subtilis* | 67 | 1.7 |
| 3OWW^27^ | Domain II of glycine riboswitch with glycine | *Vibrio cholerae* | 88 | 2.8 |
| 3F2Q^28^ | FMN riboswitch bound to FMN | *Fusobacterium nucleatum* | 112 | 2.95 |
| 3DIL^29^ | Lysine riboswitch bound to lysine | *Thermotoga maritima* | 174 | 1.9 |
| 3MXH^30^ | c-di-GMP riboswitch | *Vibrio cholerae* | 92 | 2.3 |
| 2GDI^31^ | TPP riboswitch | *Escherichia coli* | 80 | 2.05 |
| 3D2V^32^ | TPP-specific riboswitch | *Arabidopsis thaliana* | 77 | 2.0 |
|  |  |  |  |  |
| Ribosomes | | | | |
| 3OFO^33^ | 16S ribosomal subunit | *Escherichia coli* | 1533 | 3.1 |
| 3OFR^33^ | 23S ribosomal subunit | *Escherichia coli* | 2904 | 3.1 |
| 1VQO^34^ | 23S ribosomal subunit | *Haloarcula marismortui* | 2922 | 2.2 |
|  |  |  |  |  |
| SRP RNAs | | | | |
| 1MFQ^35^ | 7S RNA of SRP | *Homo sapiens* | 128 | 3.1 |
| 1LNG^36^ | SRP19-7S.S SRP RNA complex | *Methanocaldococcus jannaschii* | 97 | 2.3 |
| 3KTW^37^ | SRP19/S-domain SRP RNA complex | *Sulfolobus solfataricus* | 96 | 3.2 |
|  |  |  |  |  |
| Other RNAs | | | | |
| 2IL9^38^ | Ribosomal binding domain of the IRES RNA | *Plautia stali intestine virus* | 142 | 3.1 |
| 1KH6^39^ | JIIIabc (IRES) | Hepatitis C virus | 53 | 2.9 |
| 2CZJ^40^ | tRNA domain of tmRNA | *Thermus thermophilus* | 63 | 3.01 |

1. Reiter NJ, Osterman A, Torres-Larios A, Swinger KK, Pan T, Mondragón A. (2010) Nature 468:784-9.
2. Krasilnikov AS, Xiao Y, Pan T, Mondragón A. (2004) Science 306:104-7.
3. Kazantsev AV, Krivenko AA, Harrington DJ, Holbrook SR, Adams PD, Pace NR. (2005) Proc Natl Acad Sci U S A 102:13392-7.
4. Krasilnikov AS, Yang X, Pan T, Mondragón A. (2003) Nature 421:760-4.
5. Ye JD, Tereshko V, Frederiksen JK, Koide A, Fellouse FA, Sidhu SS, Koide S, Kossiakoff AA, Piccirilli JA. (2008) Proc Natl Acad Sci U S A 105:82-7.
6. Guo F, Gooding AR, Cech TR. (2004) Mol Cell 16:351-62.
7. Adams PL, Stahley MR, Kosek AB, Wang J, Strobel SA. (2004) Nature 430:45-50.
8. Golden BL, Kim H, Chase E. (2005) Nat Struct Mol Biol 12:82-9.
9. Zhang L, Doudna JA. (2002) Science 295:2084-8.
10. Toor N, Keating KS, Fedorova O, Rajashankar K, Wang J, Pyle AM. (2010) RNA 16:57-69.
11. Chen JH, Yajima R, Chadalavada DM, Chase E, Bevilacqua PC, Golden BL. (2010) Biochemistry 49:6508-18.
12. Rupert PB, Massey AP, Sigurdsson ST, Ferré-D'Amaré AR. (2002) Science 298:1421-4.
13. Chi YI, Martick M, Lares M, Kim R, Scott WG, Kim SH. (2008) PLoS Biol Sep 30;6(9):e234.
14. Shechner DM, Grant RA, Bagby SC, Koldobskaya Y, Piccirilli JA, Bartel DP. (2009) Science 326:1271-5.
15. Xiao H, Murakami H, Suga H, Ferré-D'Amaré AR. (2008) Nature 454:358-61.
16. Klein DJ, Wilkinson SR, Been MD, Ferré-D'Amaré AR. (2007) J Mol Biol 373:178-89.
17. Cochrane JC, Lipchock SV, Smith KD, Strobel SA. (2009) Biochemistry 48:3239-46.
18. Robertson MP, Scott WG. (2007) Science 315:1549-53.
19. Montange RK, Mondragón E, van Tyne D, Garst AD, Ceres P, Batey RT. (2010) J Mol Biol 396:761-72.
20. Gilbert SD, Rambo RP, Van Tyne D, Batey RT. (2008) Nat Struct Mol Biol 15:177-82.
21. Lu C, Smith AM, Fuchs RT, Ding F, Rajashankar K, Henkin TM, Ke A. (2008) Nat Struct Mol Biol. 15:1076-83.
22. Lu C, Ding F, Chowdhury A, Pradhan V, Tomsic J, Holmes WM, Henkin TM, Ke A. (2010) J Mol Biol 404:803-18.
23. Dann CE 3rd, Wakeman CA, Sieling CL, Baker SC, Irnov I, Winkler WC. (2007) Cell 130:878-92.
24. Dixon N, Duncan JN, Geerlings T, Dunstan MS, McCarthy JE, Leys D, Micklefield J. (2010) Proc Natl Acad Sci U S A. 107:2830-5.
25. Edwards AL, Reyes FE, Héroux A, Batey RT. (2010) RNA. 16:2144-55.
26. Gilbert SD, Mediatore SJ, Batey RT. (2006) J Am Chem Soc. 128:14214-5.
27. Huang L, Serganov A, Patel DJ. (2010) Mol Cell. 40:774-86.
28. Serganov A, Huang L, Patel DJ. (2009) Nature 458:233-7.
29. Serganov A, Huang L, Patel DJ. (2008) Nature 455:1263-7.
30. Smith KD, Lipchock SV, Livingston AL, Shanahan CA, Strobel SA. (2010) Biochemistry 49:7351-9.
31. Serganov A, Polonskaia A, Phan AT, Breaker RR, Patel DJ. (2006) Nature 441:1167-71.
32. Thore S, Frick C, Ban N. (2008) J Am Chem Soc 130:8116-7.
33. Dunkle JA, Xiong L, Mankin AS, Cate JH. (2010) Proc Natl Acad Sci U S A. 107:17152-7.
34. Schmeing TM, Huang KS, Kitchen DE, Strobel SA, Steitz TA. (2005) Mol Cell 20:437-48.
35. Kuglstatter A, Oubridge C, Nagai K. (2002) Nat Struct Biol 9:740-4.
36. Hainzl T, Huang S, Sauer-Eriksson AE. (2002) Nature 417:767-71.
37. Wild K, Bange G, Bozkurt G, Segnitz B, Hendricks A, Sinning I. (2010) Acta Crystallogr D Biol Crystallogr 66:295-303.
38. Pfingsten JS, Costantino DA, Kieft JS. (2006) Science 314:1450-4.
39. Kieft JS, Zhou K, Grech A, Jubin R, Doudna JA. (2002) Nat Struct Biol. 9:370-4.
40. Bessho Y, Shibata R, Sekine S, Murayama K, Higashijima K, Hori-Takemoto C, Shirouzu M, Kuramitsu S, Yokoyama S. (2007) Proc Natl Acad Sci U S A 104:8293-8.
