# Supplementary material for: Structural Variation and Uniformity among Tetraloop-Receptor Interactions and Other Loop-Helix Interactions in RNA Crystal Structures
Source: PLoS One. 2012 Nov 9;7(11):e49225. doi: 10.1371/journal.pone.0049225 (PMC3494683; doi:10.1371/journal.pone.0049225)
Supplement: Table S2 — List of PDB substructures containing RNA loop-helix interactions (derived from Table S1). (DOC) [file pone.0049225.s011.doc]

**Table S2. List of PDB substructures containing RNA loop-helix interactions (derived from Supplementary Table 1).**

| PDB ID^1^ | Loop  Residues^2^ | Receptor  Residues^2^ | Macromolecule Name | Source | Length (nts) | Resolution (Å) |
| --- | --- | --- | --- | --- | --- | --- |
| RNase P | | | | | | |
| 3OK7 | 93-96 | 3-4, 340-341 | RNase P holoenzyme with tRNA (type A) | *Thermotoga maritima* | 347 | 3.8 |
|  | 285-288 | 75-76, 84-85 |  |  |  |  |
| 1U9S | 182-188 | 135-136, 162-163 | RNase P RNA specificity domain (type A) | *Thermus thermophilus* | 161 | 2.9 |
|  | 205-208 | 80-81, 93-94 |  |  |  |  |
| 2A64 | 98-107 | 55-56, 392-393 | RNase P RNA (type B) | *Bacillus stearothermophilus* | 417 | 3.3 |
| 1NBS | 175-179 | 132, 234-235 | RNase P RNA specificity domain (type B) | *Bacillus subtilis* | 155 | 3.15 |
|  | 205-208 | 145-150, 159-163 |  |  |  |  |
| Group I introns | | | | | | |
| 2R8S | 150-153 | 222-227, 247-251 | P4-P6 ribozyme domain | *Tetrahymena thermophila* | 159 | 1.95 |
| 1X8W | 323-326 | 118-119, 202-203 | Group I ribozyme | *Tetrahymena thermophila* | 247 | 3.8 |
| 1U6B | 24-27 | 146-151, 160-164 | Group I ribozyme with both exons | *Azoarcus sp.* | 197 | 3.1 |
|  | 189-192 | 60-65, 80-84 |  |  |  |  |
| 1Y0Q | 22-25 | 170-171, 177-178 | Group I ribozyme | *Staphylococcus* phage Twort | 229 | 3.6 |
|  | 205-208 | 60-61, 78-79 |  |  |  |  |
| Group II introns | | | | | | |
| 3IGI | 90-93 | 272-273, 280-281 | Group IIC intron | *Oceanobacillus iheyensis* | 412 | 3.12 |
|  | 369-372 | 128-129, 234-238 |  |  |  |  |
| Small ribozymes | | | | | | |
| 2Z75 | 114-117 | 10-11, 30-31 | GlmS ribozyme RNA | *Thermoanaerobacter tengcongensis* | 125 | 1.7 |
| Riboswitches | | | | | | |
| 2QBZ | 100-106 | 21, 167-168 | M-Box riboswitch aptamer domain | *Bacillus subtilis* | 161 | 2.6 |
| 3DIL | 125-129 | 23-24, 68-69 | Lysine riboswitch bound to lysine | *Thermotoga maritima* | 174 | 1.9 |
| 3MXH | 32-35 | 59-60, 78-79 | c-di-GMP riboswitch | *Vibrio cholerae* | 92 | 2.3 |
| 2GDI | 67-72 | 21-22, 37-38 | TPP riboswitch | *Escherichia coli* | 80 | 2.05 |
| 3D2V | 55-59 | 13-14, 25-26 | TPP-specific riboswitch | *Arabidopsis thaliana* | 77 | 2 |
| SRP RNA | | | | | | |
| 1MFQ | 147-150 | 197-198, 201-202 | 7S RNA of SRP | *Homo sapiens* | 128 | 3.1 |
|  | 169-174 | 126-127, 223-224 |  |  |  |  |
| 1LNG | 163-166 | 208-209, 212-213 | SRP19-7S.S SRP RNA complex | *Methanocaldococcus jannaschii* | 97 | 2.3 |
| 3KTW | 164-167 | 209-210, 213-214 | SRP19/S-domain SRP RNA complex | *Sulfolobus solfataricus* | 96 | 3.2 |
| Ribosomes | | | | | | |
| 3OFO | 159-162 | 341-342, 347-348 | 16S rRNA | *Escherichia coli* | 1533 | 3.1 |
|  | 461-470 | 202-203, 214-215 |  |  |  |  |
|  | 523-526 | 11-12, 22-23 |  |  |  |  |
|  | 898-901 | 769-770, 809-810 |  |  |  |  |
|  | 1013-1016 | 987-988, 1217-1218 |  |  |  |  |
|  | 1077-1080 | 16-17, 918-919 |  |  |  |  |
|  | 1166-1170 | 1088-1089, 1096-1097 |  |  |  |  |
|  | 1266-1269 | 1311-1312, 1325-1326 |  |  |  |  |
|  | 1516-1519 | 1404-1405, 1496-1497 |  |  |  |  |
| 3OFR | 124-127 | 54-55, 115-116 | 23S rRNA | *Escherichia coli* | 2904 | 3.1 |
|  | 159-167 | 2206-2207, 2217-2218 |  |  |  |  |
|  | 226-229 | 409-410, 417-418 |  |  |  |  |
|  | 630-633 | 2401-2403, 2414-2415 |  |  |  |  |
|  | 642-646 | 2348-2349, 2368-2369 |  |  |  |  |
|  | 956-961 | 2456-2457, 2494-2495 |  |  |  |  |
|  | 1364-1367 | 186-187, 209-210 |  |  |  |  |
|  | 1493-1497 | 1418-1421, 1577-1580 |  |  |  |  |
|  | 1728-1732 | 1516 |  |  |  |  |
|  | 1807-1810 | 1362-1363, 1368-1369 |  |  |  |  |
|  | 2210-2214 | 1359-1360, 1371-1372 |  |  |  |  |
|  | 2552-2556 | 2507, 2581-2582 |  |  |  |  |
|  | 2857-2860 | 1708-1709, 1749-1750 |  |  |  |  |
| 1VQO | 119-121 | 50-51, 110-111 | 23S rRNA | *Haloarcula marismortui* | 2922 | 2.2 |
|  | 196-200 | 415-416, 424-425 |  |  |  |  |
|  | 218-222 | 164-165, 170-171 |  |  |  |  |
|  | 391-398 | 2441-2442, 2450-2451 |  |  |  |  |
|  | 469-472 | 773-774, 887-888 |  |  |  |  |
|  | 577-580 | 1110-1111, 1252-1253 |  |  |  |  |
|  | 671-675 | 36, 446 |  |  |  |  |
|  | 691-694 | 2439-2440, 2452-2453 |  |  |  |  |
|  | 734-737 | 2382-2383, 2405-2406 |  |  |  |  |
|  | 838-845 | 1369-1371, 2054-2055 |  |  |  |  |
|  | 873-877 | 1832, 1844 |  |  |  |  |
|  | 1055-1059 | 2491-2492, 2529-2530 |  |  |  |  |
|  | 1077-1082 | 2067-2068, 2077-2078 |  |  |  |  |
|  | 1327-1330 | 905-906, 1299-1300 |  |  |  |  |
|  | 1469-1473 | 156-157, 179-180 |  |  |  |  |
|  | 1499-1506 | 1420-1421, 1443-1444 |  |  |  |  |
|  | 1595-1599 | 1537-1538, 1647-1648 |  |  |  |  |
|  | 1629-1632 | 1553-1554, 1567-1568 |  |  |  |  |
|  | 1706-1712 | 790-791, 823-824 |  |  |  |  |
|  | 1770-1773 | 1829, 1885, 2017-2018 |  |  |  |  |
|  | 1834-1842 | 2621-2622, 2642-2643 |  |  |  |  |
|  | 1863-1866 | 1467-1468, 1474-1475 |  |  |  |  |
|  | 1917-1922 | 418-419, 2448-2449 |  |  |  |  |
|  | 1991-1997 | 2583-2584, 2594-2595 |  |  |  |  |
|  | 2069-2076 | 2490, 2531 |  |  |  |  |
|  | 2301-2306 | 952, 1014-1015 |  |  |  |  |
|  | 2390-2398 | 915-916, 927-928 |  |  |  |  |
|  | 2564-2569 | 2695-2696, 2699-2670 |  |  |  |  |
|  | 2630-2633 | 2114-2115, 2470-2471 |  |  |  |  |
|  | 2784-2788 | 1153, 1213 |  |  |  |  |
|  | 2837-2843 | 2087-2088, 2656-2657 |  |  |  |  |

1. See Table S1 for individual PDB references.
2. Numbering according to PDB file residue numbering.
