# Supplementary material for: Structural Variation and Uniformity among Tetraloop-Receptor Interactions and Other Loop-Helix Interactions in RNA Crystal Structures
Source: PLoS One. 2012 Nov 9;7(11):e49225. doi: 10.1371/journal.pone.0049225 (PMC3494683; doi:10.1371/journal.pone.0049225)
Supplement: Table S3 — Sequences of tetraloops and receptors arranged according to structural subclasses. (DOC) [file pone.0049225.s012.doc]

**Table S3. Sequences of tetraloops and receptors arranged according to structural subclasses.**

| A. GAAA tetraloop/11-nt receptor | | | | | | |
| --- | --- | --- | --- | --- | --- | --- |
| Class | Subclass^1^ | PDB ID^2^ | Loop nts | Receptor nts | Loop sequence^3^ | Receptor sequence^4^ |
| I |  | 1NBS | 205-208 | 145-150,159-163 | GAAA | UAUGG/CCUACG |
| I |  | 1U6B | 24-27 | 146-151, 160-164 | GAAA | UAUGG/CCUAAG |
| I |  | 1U6B | 189-192 | 60-65, 80-84 | GAAA | UAUGG/CCUAAG |
| I |  | 2R8S | 150-153 | 222-227, 247-251 | GAAA | UAUGG/CCUAAG |
| B. GNRA tetraloop/minor groove receptor (and GNRA-like structures having similar interactions) | | | | | | |
| II | 1.1.1 | 2Z75 | 114-117 | 10-11, 30-31 | GCGA | AG/CU |
| II | 1.1.1 | 3IGI | 90-93 | 272-273, 280-281 | GCGA | AG/CU |
| II | 1.1.1 | 3OK7 | 93-96 | 3-4, 340-341 | GUGA | AG/CU |
| II | 1.1.1 | 3OK7 | 285-288 | 75-76, 84-85 | GUAA | GG/CC |
| II | 1.1.1 | 3OFO | 1077-1080 | 16-17, 918-919 | GUGA | AA/AU |
| II | 1.1.1 | 3OFO | 1266-1269 | 1311-1312, 1325-1326 | GCGA | AG/CU |
| II | 1.1.1 | 3OFR | 2857-2860 | 1708-1709, 1749-1750 | GCGA | AG/CU |
| II | 1.1.2 | 1U9S | 205-208 | 80-81, 93-94 | GCAA | GG/CC |
| II | 1.1.2 | 3MXH | 32-35 | 59-60, 78-79 | GAAA | AG/CU |
| II | 1.1.2 | 1VQO | 1629-1632 | 1553-1554, 1567-1568 | GAAA | AG/CU |
| II | 1.1.2 | 1VQO | 1863-1866 | 1467-1468, 1474-1475 | GCAA | CG/CG |
| II | 1.1 (Indiv) | 1Y0Q | 22-25 | 170-171, 177-178 | GUAA | GG/CC |
| II | 1.1 (Indiv) | 1Y0Q | 205-208 | 60-61, 78-79 | GAAA | AG/CU |
| II | 1.1 (Indiv) | 1VQO | 469-472^5^ | 773-774, 887-888^5^ | GUGA | GU/AC |
| II | 1.1 (Indiv) | 1VQO | 577-580 | 1110-1111, 1252-1253 | GUGA | AC/GU |
| II | 1.1 (Indiv) | 1VQO | 1327-1330^5^ | 905-906, 1299-1300^5^ | GAAA | GG/CC |
| II | 1.1 (Indiv) | 1VQO | 2630-2633^5^ | 2114-2115, 2470-2471^5^ | GUGA | AG/CU |
| II | 1.1 (Indiv) | 3OFO | 1013-1016 | 987-988, 1217-1218 | GAGA | GG/CC |
| II | 1.1 (Indiv) | 3OFR | 1807-1810 | 1362-1363, 1368-1369 | GAAA | GG/CC |
| II | 1 (Indiv) | 1X8W | 323-326 | 118-119, 202-203 | GAAA | UC/GG |
| II | 1 (Indiv) | 1VQO | 734-737 | 2382-2383, 2405-2406 | UCAA | AG/CU |
| II | 1 (Indiv) | 3OFO | 898-901 | 769-770, 809-810 | GCAA | GC/GC |
| II | 1 (Indiv) | 3OFO | 1516-1519 | 1404-1405, 1496-1497 | GGAA | CG/CG |
| II | 1 (NTL) | 1MFQ | 169-174 | 126-127, 223-224 | [GCCU]--AA[GG] | CA/UG |
| II | 1 (NTL) | 1NBS | 175-179 | 132, 234-235 | [UG]--AA[A] | G/C |
| II | 1 (NTL) | 1U9S | 182-188 | 135-136, 162-163 | [GU]--AA[GAG] | GG/CC |
| II | 1 (NTL) | 2GDI | 67-72 | 21-22, 37-38 | [AU]--AA[UG] | GC/GC |
| II | 1 (NTL) | 3D2V | 55-59 | 13-14, 25-26 | [GUA]--AU[G] | GC/GC |
| II | 1 (NTL) | 1VQO | 119-121 | 50-51, 110-111 | [GAA]---U[C] | G/C |
| II | 1 (NTL) | 1VQO | 873-877^5^ | 1832, 1844^5^ | [G]---A[AAG] | G/C |
| II | 1 (NTL) | 1VQO | 1055-1059 | 2491-2492, 2529-2530 | GUAA[G] | GC/GU |
| II | 1 (NTL) | 1VQO | 1077-1082^5^ | 2067-2068, 2077-2078^5^ | [GG]--AA[CAA] | AG/CU |
| II | 1 (NTL) | 1VQO | 1499-1506^5^ | 1420-1421, 1443-1444^5^ | [U]--AA[U] | GG/CC |
| II | 1 (NTL) | 1VQO | 1991-1997^5^ | 2583-2584, 2594-2595^5^ | [AU]--CA[GUA] | AG/CU |
| II | 1 (NTL) | 3OFO | 1166-1170 | 1088-1089, 1096-1097 | [GAU]--AA | GG/CC |
| II | 1 (NTL) | 3OFR | 956-961 | 2456-2457, 2494-2495 | [GCU]--AA[C] | GG/CU |
| II | 2 | 1VQO | 691-694 | 2439-2440, 2452-2453 | GAAA | CC/GG |
| II | 2 | 3OFR | 630-633 | 2401-2403, 2414-2415 | GAAA | U[U]C/GG |
| II | 2 | 3OFR | 1364-1367 | 186-187, 209-210 | GAAA | CC/GG |
| II | 2 (NTL) | 1VQO | 1469-1473 | 156-157, 179-180 | CAAC[U] | CG/CG |
| II | 2 (NTL) | 1VQO | 2390-2398 | 915-916, 927-928 | [UCCC]-A--[ACGA] | C/G |
| II | 3 | 1LNG | 163-166 | 208-209, 212-213 | GUAG | AG/CG |
| II | 3 | 1MFQ | 147-150 | 197-198, 201-202 | GGAG | AC/GG |
| II | 3 | 3KTW | 164-167 | 209-210, 213-214 | GGAG | AG/CG |
| II | 3(NTL) | 1VQO | 2564-2569^5^ | 2695-2696, 2699-2670^5^ | [GC]--AG[AA] | AG/CG |
| II | 4 | 3OFO | 159-162 | 341-342, 347-348 | GAAA | GG/CC |
| II | 4 (NTL) | 1VQO | 1595-1599 | 1537-1538, 1647-1648 | [G]UAAU | GG/CC |
| II | 5^2^ | 3IGI | 369-372 | 128-129, 234-238 | GAAC | -/G |
| C. Tetraloop-like structures interacting with a helix (nonstandard) | | | | | | |
| III | 1 (NTL) | 1VQO | 2837-2843^5^ | 2087-2088, 2656-2657^5^ | [UAC]--AA[GA] | GG/CC |
| III | 1 (NTL) | 3OFR | 642-646 | 2348-2349, 2368-2369 | UAAC[U] | UG/CA |
| III | Indiv (NTL) | 3DIL | 125-129 | 23-24, 68-69 | GAA[U]A | GG/AU |
| III | Indiv (NTL) | 1VQO | 218-222^5^ | 164-165, 170-171^5^ | CG[C]GA | UC/GA |
| III | Indiv (NTL) | 1VQO | 1706-1712^5^ | 790-791, 823-824^5^ | GCG[A]A | UG/AA |
| D. Non-GNRA structures interacting with a helix | | | | | | |
| IV | 1 (NTL) | 2A64 | 98-107 | 55-56, 392-393 | [AAUACCU]AA[G] | AU/AU |
| IV | 1 | 3OFR | 124-127 | 54-55, 115-116 | [GA]AA | GG/CC |
| E. Single base inserted into receptor helix | | | | | | |
| IV | Indiv (NTL) | 2QBZ | 100-106 | 21, 167-168 | [G]ACA[UAA] | C-/UG |
| IV | Indiv (NTL) | 1VQO | 2301-2306^5^ | 952, 1014-1015^5^ | [A]AA[GAU] | G-/AC |
| F. Surface formed by splayed nts | | | | | | |
| IV | Indiv (NTL) | 1VQO | 2069-2076^5^ | 2490, 2531^5^ | [UGC]G[GAGU] | U/A |
| IV | Indiv (NTL) | 3OFR | 2210-2214 | 1359-1360, 1371-1372 | [UA]A[U]C | AG/CU |
| IV | Indiv (NTL) | 3OFR | 1493-1497 | 1418-1421, 1577-1580 | [C]A[AAU] | A[A]G/CU |
| G. Other unique interactions | | | | | | |
| IV | Indiv (NTL) | 1VQO | 196-200 | 415-416, 424-425 | [UA]A[C]G | CU/AG |
| IV | Indiv | 1VQO | 1770-1773^5^ | 1829, 1885, 2017-2018^5^ | [U]U[CG] | UA/AA |
| IV | Indiv (NTL) | 1VQO | 1834-1842^5^ | 2621-2622, 2642-2643^5^ | [CUAG]UA[ACA] | UA/G- |
| IV | Indiv (NTL) | 1VQO | 1917-1922 | 418-419, 2448-2449 | [GUA]CA[A] | UG/CA |
| IV | Indiv (NTL) | 3OFO | 461-470 | 202-203, 214-215 | [AGUU]A[AU]A[CC] | CC/GG |
| IV | Indiv | 3OFO | 523-526 | 11-12, 22-23 | [A]G[CC] | GU/GC |
| IV | Indiv (NTL) | 3OFR | 159-167 | 2206-2207, 2217-2218 | [AUACCU]A[AG] | CC/GG |
| IV | Indiv | 3OFR | 226-229 | 409-410, 417-418 | [C]CAA | CC/GG |
| IV | Indiv (NTL) | 3OFR | 2552-2556 | 2507, 2581-2582 | [U]G[UUC] | C/G |
| H. Single base makes almost all of the interaction, not a large interaction surface | | | | | | |
| IV | Indiv (NTL) | 1VQO | 391-398 | 2441-2442, 2450-2451 | [UUGG]AUA[U] | UGC/-CG |
| IV | Indiv (NTL) | 1VQO | 671-675 | 36, 446 | [AGU]A[U] | UC/GA |
| IV | Indiv (NTL) | 1VQO | 838-845 | 1369-1371, 2054-2055 | [CC]U[ACAAU] | A/A |
| IV | Indiv (NTL) | 1VQO | 2784-2788^5^ | 1153, 1213^5^ | [AC]G[CA] | CA/C- |
| IV | Indiv (NTL) | 3OFR | 1728-1732 | 1516 | [CUCG]C | G/G |

1. “Indiv” indicates an “individual” structure that does not form an additional level of subgrouping based on superposition. “NTL” indicates a structure that does not have the GNRA tetraloop geometry.
2. See Table S1 for individual PDB references.
3. Nucleotides in square brackets indicate loop residues that do not conform to the GNRA tetraloop geometry in their interactions with the receptor (used only for classes I, II and III, in which the loops have a GNRA or GNRA-like structure). Dashes indicate “missing” tetraloop positions. For example, [UG]--AA[A] means that AA has the structure of T3 and T4 in a GNRA tetraloop, while the bracketed UG and A have positions that don’t correspond to the GNRA structure. Underlines indicate the loop nucleotides that interact with the receptor for class IV structures, which have irregular loop geometries not readily comparable with the GNRA geometry.
4. Receptor sequences are written as “left-strand sequence/right-strand sequence” according to the secondary structures in Supporting Figure 3. “Dashes” indicate a missing base in a receptor basepair. “Square brackets” indicates extra nucleotides in the receptor sequence
5. *Haloarcula* structural portions that are found in the *E. coli* ribosome with essentially identical structures. The E. coli structures are omitted from the table.
